# Supplementary material for: Which ethical values underpin England’s National Health Service reset of paediatric and maternity services following COVID-19: a rapid review
Source: BMJ Open. 2021 Jun 8;11(6):e049214. doi: 10.1136/bmjopen-2021-049214 (PMC8189755; doi:10.1136/bmjopen-2021-049214)
Supplement: Supplementary data [file bmjopen-2021-049214supp001.pdf]

Which ethical values underpin England's National Health Service reset of paediatric and maternity services following Covid-19: a rapid review.

SUPPLIMENTARY FILES

FILE 1: RAPID REVIEW PROTOCOL

Background and review rationale:

The response to Covid 19 (C19) will have far-reaching consequences for the NHS. The *Everyday and pandemic ethics* project will explore how the ethical issues created by this response have been approached by providers of non-C19 services. Notably we will explore how decisions on service prioritisation and reconfiguration have been made in the "reset" phase that has followed the first acute phase of the C19 pandemic. We define this "reset" phase as commencing from April 29th 2020, as NHS services were instructed on that date to prepare to recommence the delivery of non-covid surgical services (https://www.england.nhs.uk/coronavirus/wp-content/uploads/sites/52/2020/04/second-phase-of-nhs-response-to-covid-19-letter-to-chief-execs-29-april-2020.pdf). The "resetting" of NHS services encompasses the following:

- The resumption of service delivery incorporating revised procedures and practices to control the spread of C19 (e.g. the wearing of face coverings);
- Preparation for, and management of, second "waves" or recurrent spikes of C19, at both the national and local levels;
- The opportunities to reconfigure health services, for example accelerating the use of tele-medicine.

The focus on the reset phase emphasises the unique factors affecting ethical decision-making as services are re-established following the acute phase of the C19 pandemic.

We will focus on ethical decision-making in two non-C19 areas: maternity and paediatrics. We have chosen these areas because they have been significantly affected by the C19 response due to resource allocation away from these areas, with professional and patient organisations highlighting problematic effects on both areas (Association of Paediatric Anaestetists of Great Britain and Ireland, 2020; First 1001 Days Movement, 2020; McDonald et al., 2020). Specifically, the review will focus on "maternity services" (pre-natal, intrapartum, and post-partum care); and the resumption of paediatric surgery (encompassing critical / intensive care admissions, surgery, hospital discharge, and aftercare, referred to as "paediatric critical care and surgery services") during the C19 reset phase.

The objective of this review is to provide an initial understanding of the ethical values explicitly or implicitly engaged to inform decision-making about maternity services, and the resumption of paediatric critical care and surgery during the reset phases following the C19 pandemic in England. We adopt a pragmatic approach in order to make the best available use of existing evidence relating to this topic. The evidence will include diverse sources such as Government and Hospital trust policies, statements and decision support tools; reports and statements from professional bodies and charitable organisations; and evidence reviews and commentaries in academic journals. The approach aims to be broad and inclusive by combining searches of bibliographic databases with grey literature, hand searching, snowballing references of included sources, and engaging key topic stakeholders in an effort to verify completeness of sources. These approaches aim to ensure flexibility in identifying relevant sources both systematically and in the most efficient and pragmatic manner.

We will report key characteristics of all sources, and will appraise sources against a coding framework adapted from the Ethical Framework embedded in the Government's Pandemic Flu policy (UK Government, 2017). This framework is intended to guide all UK NHS decision-making during the rapid

readjustment of services due to a pandemic. Recognising that the reset phase requires different decision-making to the acute phase, we have adapted the framework by drawing upon two interlinked national documents (a letter on "Third phase of NHS response to Covid", 31st July 2020 (Stevens & Pritchard, 2020); and the National Voices "Five principles for the next phase of the Covid-19 response", published June 2020 (National Voices, 2020)). These adaptations aim to reflect the particular ethical considerations relevant to the "reset" phase. We recognise that this adaptation creates a tension between the rapid review methodology and findings, which we discuss alongside the revised framework below. In our analysis we will draw upon the systematic review of reasons approach (Strech & Sofaer, 2012) to facilitate explicit consideration of ethical values being applied to inform decision-making in non-C19 maternity services, and paediatric critical care and surgery services during the C19 reset phases in England.

This rapid evidence review forms the first stage of a larger project, providing a snapshot of ethical decision-making in maternity and paediatric care to inform subsequent stages of the *Everyday and Pandemic Ethics* study. Review findings will be available as immediate recommendations for ethical best practice – for example by examining the transparency of written policies against standards in the 2016 Pandemic Flu Policy - for paediatric and maternity services delivery during the C19 reset phases.

Objective

The objective of this review is to answer the question: what ethical values guide decision-making in non-C19 paediatric critical care and surgery and maternity services during the C19 reset phases in England? Achieving this objective will entail exploring a range of decision-making factors, such how are involved in decision-making, what decisions have been made, and how decisions are justified, identifying implicit and explicit ethical values.

Methodology

To ensure a rigorous review methodology, we have drawn upon the ENTREQ guidelines for qualitative research synthesis (Tong, Flemming, McInnes, Oliver, & Craig, 2012) and the systematic review of reasons approach developed for normative review questions (Strech & Sofaer, 2012). Integrating these approaches address the critique that literature reviews exploring normative considerations often fail to clearly report the methodological approach taken (Mertz, Strech, & Kahrass, 2017).

Inclusion and exclusion criteria

Inclusion criteria

This review will consider sources developed to guide non-C19 paediatric critical care and surgery services and maternity services during the reset phases of C19; or that discuss the application of ethical values to paediatric critical care and surgery services and maternity services during the reset phases of C19.

The review will include sources relating to England, including national policies (that include England), and policies from Trusts and individual hospitals across England, including our case study sites (in North West England and the Midlands). We will be restricted to sources written in the English language, and published after 29th April 2020.

Exclusion criteria

Sources published prior 29th April 2020, that discuss healthcare delivery broadly; or that discuss maternity or paediatric critical care or surgery services during the acute phase of the C19 pandemic in England (defined as the start of lockdown on 23rd March until the 29th April 2020) will be excluded.

Data sources

The review will include the following data sources:

- National policies guiding the implementation of non-C19 maternity services, and/or paediatric critical care and surgery services; and/or providing an ethical framework or decision-making tools for healthcare reorganisation of these services during the C19 reset phases;
- Local trust and hospital policies guiding the implementation of non-C19 maternity and paediatric critical care and surgery services; and/or providing an ethical framework or decision-making tools for healthcare reorganisation in these services during the C19 reset phases;
- Guidelines and statements from Royal Medical Colleges relating to the implementation of non-C19 maternity and paediatric critical care and surgery services and/or providing an ethical framework or decision-making tools for healthcare reorganisation in these services during the C19 reset phases;
- Working papers and committee reports discussing the re-orientation of non-C19 maternity and paediatric critical care and surgery services during the C19 reset phases;
- Evidence reviews and primary qualitative and quantitative research on the re-orientation of non-C19 maternity and paediatric critical care and surgery services during the C19 reset phases;
- Peer-reviewed commentaries and grey-literature discussing experiences of non-C19 maternity, and paediatric critical care and surgery services during the C19 reset phases.

All sources will be obtained from online platforms, or via e-mail for Freedom of Information requests and stakeholder contributions.

Electronic search strategy

We will conduct searches in September 2020, with an additional search prior to the publication of the review to check for sources published in the interim. We will search the following academic bibliographic databases: PubMed and PubMeds Covid-19 database LitCOVID (https://www.ncbi.nlm.nih.gov/research/coronavirus/). We will also search clearing houses of C19 related research including the EPPI Centre living map of Covid-19 evidence (http://eppi.ioe.ac.uk/cms/Projects/DepartmentofHealthandSocialCare/Publishedreviews/COVID-19Livingsystematicmapoftheevidence/tabid/3765/Default.aspx), COVID END (https://www.mcmasterforum.org/networks/covid-end), evidence aid (https://evidenceaid.org/evidence/coronavirus-covid-19/ - which includes reviews being conducted by the Campbell Collaboration), and the Cochrane Collaboration.

For academic bibliographic databases we will search using the following terms:

1. (Covid OR Covid-19 OR coronavirus* OR SARS-CoV-2 OR Severe Acute Respiratory Syndrome OR pandemic) AND
2. (Matern*) OR (pre-natal OR inter-partum OR post-natal OR perinatal) OR (labour OR pregnan*) OR (obstetrics) OR (birth*) OR (Midwife*) AND
3. (paediatric OR pediatric) AND (critical OR intensive OR acute) OR (operati* OR theatre*) OR (child*) OR (surg*) AND
4. (doctor) OR (nurs*) AND
5. (service*) OR (design OR deliver*) OR (allocat* OR priorit*) OR (care) OR (policy OR guideline*)

Searchers will be conducted step-wise, first conducting searches relating to Maternity service combining rows 1,2, 4 and 5 above; and secondly for Paediatric critical care and surgery, combining rows 1,3, 4 and 5 above.

To complement academic databases, and recognising the scope of the research question, we will also search grey literature sources including the websites of NHS Trusts (including our case study sites),

the UK Government (gov.uk), and websites of professional bodies (e.g. Academy of Royal Colleagues and the Royal College of Paediatrics / Midwifery and NICE). We will also search clearinghouses of C19 related grey literature such as policy documents, for example the Health Foundation C19 Policy Tracker (https://www.health.org.uk/news-and-comment/charts-and-infographics/covid-19-policy-tracker).

Study screening methods

We will review all identified sources and any duplicates removed. Two members of the research team (AC, PB, CR, SF and LF) will double screen all identified results. Screening will be based on title and abstract / summary (where available). Where these are not available or no definitive decision can be made about whether a source meets the review inclusion criteria based on title and abstract/summary screening, additional full text review will be undertaken. To operationalise the inclusion criteria we applied the following scoring system:

0. Not included
1. Included: Identifies the approach taken to decision making (e.g. discusses a decision-making tool or framework)
2. Included: Identifies what decision has been made
3. Included: Identifies a justification for the decision taken

Where a source meets more than one of the inclusion criteria, all will be identified. Disagreements in double screening will be resolved through discussion with a third member of the review team (HD) not involved in initial screening to reach a consensus decision about inclusion or exclusion.

We will document all searches and screening assessments in a flow chart, with an accompanying narrative explanation, including explicit reasons for study exclusion.

Using the Freedom of Information Act 2000

The Freedom of Information Act 2000 (FOI) imposes two main duties on public authorities: one to proactively provide information, and the other to respond to requests for information. A model ‘publication scheme’ has been produced which public authorities are obliged to follow in making relevant information available. The model publication scheme sets out various classes of information, which are tailored to different authorities by a ‘definition document’ for each type of organisation. The classes of information are as follows:

- Who we are and what we do
- What we spend and how we spend it
- What our priorities are and how we are doing
- How we make decisions
- Our policies and procedures
- Lists and registers
- The services we offer

To aid access to NHS Trust information we will review Trusts’ Freedom of Information Act Publication schemes and submit freedom of information (FOI) requests. Our publication scheme reviews and FOI requests will target our case study sites, as well as additional NHS Trusts with Clinical Ethics Committees as listed on the UKs Clinical Ethics Network. Both the reviews and the FOI requests will explicitly focus on sources (e.g. meeting minutes, policies, or decision-making tools) guiding maternity services and paediatric critical care and surgery services developed for the reset period. FOI requests will be submitted to individual hospitals and NHS Trusts, as well as at regional and national decision-making levels. To mirror database searches, we will repeat the publication scheme reviews and the FOI requests prior to publication of the review for the inclusion of additional sources.

After the initial searches, publication scheme reviews and results from FOI requests, we will share results with Trust and project stakeholders to conduct a completeness check and request additional missing sources be identified for screening and potential inclusion. We will furthermore search citations of included sources for snowball sampling.

Appraisal of sources

Given the reviews focus on normative values, we will apply the PROGRESS Plus tool[1] to identify the extent to which sources consider characteristics recognised to affect health equity (https://methods.cochrane.org/equity/projects/evidence-equity/progress-plus). This tool covers factors including place of residence, race/ethnicity/culture/language, occupation, gender/sex, religion, education, socioeconomic status, and social capital (O'Neill et al., 2014); as well as "plus" factors such as age and disability, relational features (such as single parent household), and time-dependent relationships (e.g. receiving in-patient care). Assessing sources against these will identify the extent to which sources are systematically considering various aspects of health equity.

In addition, for peer reviewed literature we will apply the relevant CASP checklist[2] (https://casp-uk.net/casp-tools-checklists/), and for policy sources the AGREE-II tool developed for assessing healthcare practice guidelines (Brouwers et al., 2010).

Data extraction and management

We will report the following characteristics of included sources:

- Publication type (e.g. policy, report, professional body guideline, peer reviewed article, commentary piece, decision-support tool, etc);
- Month and year of publication;
- Population (maternity or paediatric services);
- Source scope (national, regional, trust, hospital, etc);
- Where relevant for primary research we will also report: the primary research question, methodology, number of participants, and analysis approach.

Sources will be analysed against a coding framework. This coding framework has been developed by modifying the Ethical Framework embedded in the Government's Pandemic Flu policy (UK Government, 2017). The Ethical Framework in the Pandemic Flu Policy is guided by the fundamental principle of equal concern and respect, accompanied by 8 embedded principles designed to be applied as a checklist to help ensure that the full-range of ethical issues are considered in decision-making processes. It is the only framework explicitly intended to guide all UK NHS decision-making during the rapid readjustment of services due to a pandemic. However, recognising that the reset phase requires a different decision-making to the acute phase, we adapted the framework by drawing upon two interlinked national documents: (1) a letter from the NHS Chief Executive and Chief Operating Officer on "Third phase of NHS response to Covid", dated 31st July 2020 (Stevens & Pritchard, 2020), and (2) the National Voices "Five principles for the next phase of the Covid-19 response" published in June 2020 (National Voices, 2020). Our coding framework retains the Pandemic Flu 8 embedded principles, but adjusts their specification according to how they are operationalised in these two documents. We recognise this adaptation creates a methodological tension in our review as our coding framework is based upon a Framework adapted according to ethical documents relevant to the review scope and purpose. We believe this approach is justifiable given the lack of an overarching framework tailored

---

[1] This aspect of the review was not conducted due to time constraints.

[2] No peer reviewed studies reporting original data were included in the review, therefore this tool was not applied.

to the reset phase, and the need for a coding framework for the review that reflects the ethical specificities of this phase.

Extracting information from sources in relation to each of these adapted principles will identify whether the source engages with the normative values identified as important when making decisions during the C19 reset phase. The principles (retained from the national pandemic flu policy) and adapted sub-domains are as follows:

Ethical principle (from Pandemic Flu Ethical Framework)	Adapted sub-domain (based on NHS letter and National Voices Five Principles)
**Respect**	Involvement (i.e. right to express views on matters affecting them, engaging those affected by decisions)
	Respecting choices about personalised care (best interests of person as a whole)
	Collaborative working / engagement (organisational coordination; NHS volunteer scheme, clinical teams, CCGs, local authorities; co-production with voluntary sector, patient orgs etc)
**Recognising harms & balancing against benefits (physical, psychological, social & economic) - proportionality**	Recover operation of healthcare (inc. addressing backlog of care needs, resuming home visits for vulnerable / shielding where appropriate)
	Safety of NHS staff (physical, psychological, systemic inequalities, flexible working)
	Embrace new ways of working (e.g. telemedicine, home visits etc)
	Enhance crisis responsiveness (second wave)
	Accelerate preventative programmes (obesity reduction, seasonal flu, outreach to marginalised groups)
	Responsiveness (adapt plans to new circumstances / information)
**Reciprocity**	Concept of mutual exchange: take responsibility for own behaviour, reduce others expose others to risks
	Protect those at risk of C19 (physically, socially, BAME etc)
**Fairness**	Inclusivity in service recovery (e.g. barriers or access needs, support those with unequal access to care)
	Patient prioritisation (to address backlog i.e. clinical urgent / longest waiting etc)
	Reduce health inequalities (social inequalities & social determinants of health)
	Everyone matters equally & weighted equally in policies & any disproportionate impact on one particular group is accounted for
**Accountability**	Transparency (i.e. document decisions, clarity of who is responsible for decisions, governance arrangements, assess against milestones, sharing information to help others)

Recognising that the reset phase may incorporate responding to second waves of C19 infections, for example through localised lockdowns (as provided for in the UK Governments Covid-19 Contain framework: https://www.gov.uk/government/publications/containing-and-managing-local-coronavirus-covid-19-outbreaks/covid-19-contain-framework-a-guide-for-local-decision-makers), the principles and sub-domains within this assessment framework may be inductively revised on the basis of the sources reviewed. We will report any development of the framework as an outcome of the rapid review.

We will apply a scoring system to assess the inclusion and application of each principle domain. This will entail a 2-stage process, first answering "yes/no" to its inclusion and, secondly, rating application of each domain on a scale of 1-3, where:

1. ethical principle(s) inferred or mentioned but not clearly applied;
2. ethical principle(s) identified and its application described; and
3. ethical principle(s) application is discussed in-depth, including balancing against other principles.

Data synthesis

To further explore the data, we will conduct further analysis of sources from our case study sites (North West England and the Midlands) to conduct a thematic synthesis (Thomas & Harden, 2008)[3]. This approach will draw upon the review of reasons where the data is explored to identify reasons for adopting particular normative positions, and the consistency of these reasons across sources and settings (maternity or paediatrics). This will help to surface the range of reasons informing decision-making processes, and experiences of these decisions by those affected.

Data synthesis will be led by AC and PB, with regular review and discussion with the wider research team to ensure rigor of the approach to analysis.

Reporting

We will report this rapid review as brief reports summarising the approach to paediatric critical care and surgery services, and maternity services, during the reset phase of the C19 pandemic. This will identify the ethical values informing paediatric critical care and surgery services, and maternity services, during the reset phase of the C19 pandemic, and highlighting case study examples that explore the reasons for adopting a particular normative position. The report will be disseminated in the form of a short brief, shared with our stakeholder group comprised of representatives of National bodies, case study Trusts and Hospitals, and other relevant parties. We will also disseminate the review findings via social media (e.g. Twitter) and our project website (https://www.liverpool.ac.uk/population-health-sciences/departments/health-services-research/key-projects/resetethics/).

We will also develop a rapid review publication reporting the full results. It will go into more depth than the brief report about the methodology, and will offer an in-depth description of the response to planning for the reset phase of maternity services and paediatric critical care and surgery services in England. We will explore examples of good practice – such as where specific sources have engaged with the full breadth of ethical considerations, or where there is transparency in descriptions of ethical engagement and decision-making processes. From this, we will make recommendations for addressing areas where the normative basis of adopting specific approaches to service planning and delivery are unclear.

REFERENCES

Association of Paediatric Anaestetists of Great Britain and Ireland, A. (2020). APAGBI position statement regarding the delivery and recovery of Children's surgery during the coronavirus pandemic [Press release]. Retrieved from https://www.apagbi.org.uk/sites/default/files/inline-files/APA%20statement%2015.05.20%20Final.pdf

Brouwers, M., Kho, M. E., Browman, G. P., Burgers, J. S., Cluzeau, F., Feder, G., . . . Consortium, T. A. N. S. (2010). AGREE II: Advancing guideline development, reporting and evaluation in

[3] This aspect of the review is ongoing and is based primarily upon the Publication Scheme review data. In our paper we report initial findings from this.

healthcare. *Canadian Medical Association Journal, 182*(18), E839-E842. doi:10.1503/cmaj.090449

First 1001 Days Movement. (2020). Our call on Government to keep babies safe [Press release]. Retrieved from https://parentinfantfoundation.org.uk/our-call-on-government-to-keep-babies-safe/

McDonald, H. I., Tessier, E., White, J. M., Woodruff, M., Knowles, C., Bates, C., . . . Edelstein, M. (2020). Early impact of the coronavirus disease (COVID-19) pandemic and physical distancing measures on routine childhood vaccinations in England, January to April 2020. *Eurosurveillance, 25*(19), 2000848. doi:https://doi.org/10.2807/1560-7917.ES.2020.25.19.2000848

Mertz, M., Strech, D., & Kahrass, H. (2017). What methods do reviews of normative ethics literature use for search, selection, analysis, and synthesis? In-depth results from a systematic review of reviews. *Systematic Reviews, 6*(1), 261. doi:10.1186/s13643-017-0661-x

National Voices. (2020). *Five principles for the next phase of the Covid-19 response*. Retrieved from https://www.nationalvoices.org.uk/sites/default/files/public/publications/5_principles_statement_091020.pdf

O'Neill, J., Tabish, H., Welch, V., Petticrew, M., Pottie, K., Clarke, M., . . . Tugwell, P. (2014). Applying an equity lens to interventions: using PROGRESS ensures consideration of socially stratifying factors to illuminate inequities in health. *Journal of Clinical Epidemiology, 67*(1), 56-64. doi:10.1016/j.jclinepi.2013.08.005

Stevens, S., & Pritchard, A. (2020). *Third Phase of NHS Response to Covid-19*. Retrieved from https://www.england.nhs.uk/coronavirus/wp-content/uploads/sites/52/2020/07/20200731-Phase-3-letter-final-1.pdf.

Strech, D., & Sofaer, N. (2012). How to write a systematic review of reasons. *Journal of Medical Ethics, 38*(2), 121-126. doi:10.1136/medethics-2011-100096

Thomas, J., & Harden, A. (2008). Methods for the thematic synthesis of qualitative research in systematic reviews. *BMC Medical Research Methodology, 8*, 1-10. doi:10.1186/1471-2288-8-45

Tong, A., Flemming, K., McInnes, E., Oliver, S., & Craig, J. (2012). Enhancing transparency in reporting the synthesis of qualitative research: ENTREQ. *BMC Medical Research Methodology, 12*(1), 181-181. doi:10.1186/1471-2288-12-181

UK Government. (2017). *Pandemic Flu*. London, UK: UK Government Retrieved from https://www.gov.uk/guidance/pandemic-flu.

FILE 2: PUBMED SEARCH STRATEGY

Columns 1 and 2 describe the conceptual structure of the search input into PubMed. Column 3 provides an indicative example of how PubMed translated the natural language terms for each query by generating MeSH terms and using the natural language for all fields in the PubMed record. In addition to the below, in PubMed the date filter of "last 1 year", and language filter "English" were applied.

	**Natural language search terms (with wildcard truncation where relevant)**	**Search query in PubMed**
1	Covid	"sars cov 2"[MeSH Terms] OR "sars cov 2"[All Fields] OR "covid"[All Fields] OR "covid 19"[MeSH Terms] OR "covid 19"[All Fields] OR ("covid 19"[All Fields] OR "covid 19"[MeSH Terms] OR "covid 19 vaccines"[All Fields] OR "covid 19 vaccines"[MeSH Terms] OR "covid 19 serotherapy"[All Fields] OR "covid 19 serotherapy"[Supplementary Concept] OR "covid 19 nucleic acid testing"[All Fields] OR "covid 19 nucleic acid testing"[MeSH Terms] OR "covid 19 serological testing"[All Fields] OR "covid 19 serological testing"[MeSH Terms] OR "covid 19 testing"[All Fields] OR "covid 19 testing"[MeSH Terms] OR "sars cov 2"[All Fields] OR "sars cov 2"[MeSH Terms] OR "severe acute respiratory syndrome coronavirus 2"[All Fields] OR "ncov"[All Fields] OR "2019 ncov"[All Fields] OR (("coronavirus"[MeSH Terms] OR "coronavirus"[All Fields] OR "cov"[All Fields]) AND 2019/11/01:3000/12/31[Date - Publication])) OR ("coronavirus"[MeSH Terms] OR "coronavirus"[All Fields] OR "coronaviruses"[All Fields]) OR ("sars cov 2"[MeSH Terms] OR "sars cov 2"[All Fields] OR "sars cov 2"[All Fields]) OR ("severe acute respiratory syndrome"[MeSH Terms] OR ("severe"[All Fields] AND "acute"[All Fields] AND "respiratory"[All Fields] AND "syndrome"[All Fields]) OR "severe acute respiratory syndrome"[All Fields]) OR ("pandemic s"[All Fields] OR "pandemically"[All Fields] OR "pandemicity"[All Fields] OR "pandemics"[MeSH Terms] OR "pandemics"[All Fields] OR "pandemic"[All Fields])
2	Covid-19	
3	coronavirus*	
4	SARS-CoV-2	
5	Severe Acute Respiratory Syndrome	
6	Pandemic	
7	or/1-7	
8	Matern*	"matern*"[All Fields] OR ("pre-natal"[All Fields] OR "inter-partum"[All Fields] OR "post-natal"[All Fields] OR ("perinatal"[All Fields] OR "perinatally"[All Fields] OR "perinatals"[All Fields])) OR ("labor s"[All Fields] OR "labored"[All Fields] OR "laborer"[All Fields] OR "laborer s"[All Fields] OR "laborers"[All Fields] OR "laboring"[All Fields] OR "labors"[All Fields] OR "labour"[All Fields] OR "work"[MeSH Terms] OR "work"[All Fields] OR "labor"[All Fields] OR "labor, obstetric"[MeSH Terms] OR ("labor"[All Fields] AND "obstetric"[All Fields]) OR "obstetric labor"[All Fields] OR "laboured"[All Fields] OR "labourer"[All Fields] OR "labourers"[All Fields] OR "labouring"[All Fields] OR "labours"[All Fields] OR "pregnan*"[All Fields]) OR ("obstetric"[All Fields] OR "obstetrically"[All Fields] OR "obstetrics"[MeSH Terms] OR "obstetrics"[All Fields] OR
9	pre-natal OR inter-partum OR post-natal OR perinatal	
10	labour OR pregnan*	
11	Obstetrics	
12	birth*	
13	Midwife*	
14	or/8-13	

		"obstetrical"[All Fields]) OR "birth*"[All Fields] OR "midwife*"[All Fields]
15	paediatric OR pediatric	"paediatrics"[All Fields] OR "pediatrics"[MeSH Terms] OR "pediatrics"[All Fields] OR "paediatric"[All Fields] OR "pediatric"[All Fields] OR "paediatrics"[All Fields] OR "pediatrics"[MeSH Terms] OR "pediatrics"[All Fields] OR "paediatric"[All Fields] OR "pediatric"[All Fields] OR "critical"[All Fields] OR "critically"[All Fields] OR "intensive"[All Fields] OR "intensives"[All Fields] OR "acute"[All Fields] OR "acutely"[All Fields] OR "acutes"[All Fields] OR "operati*"[All Fields] OR "theatre*"[All Fields] OR "child*"[All Fields] OR "surg*"[All Fields]
16	critical OR intensive OR acute	
17	operati* OR theatre*	
18	child*	
19	surg*	
20	or/15-19	
21	Doctor	"doctor s"[All Fields] OR "doctoral"[All Fields] OR "doctorally"[All Fields] OR "doctorate"[All Fields] OR "doctorates"[All Fields] OR "doctoring"[All Fields] OR "physicians"[MeSH Terms] OR "physicians"[All Fields] OR "doctor"[All Fields] OR "doctors"[All Fields] OR "nurs*"[All Fields]
22	nurs*	
23	or/21-22	
24	service*	"service*"[All Fields] OR "design"[All Fields] OR "design s"[All Fields] OR "designabilities"[All Fields] OR "designability"[All Fields] OR "designable"[All Fields] OR "designed"[All Fields] OR "designer"[All Fields] OR "designer s"[All Fields] OR "designers"[All Fields] OR "designing"[All Fields] OR "designs"[All Fields] OR "deliver*"[All Fields] OR "allocat*"[All Fields] OR "priorit*"[All Fields] OR "care"[All Fields] OR "policy"[MeSH Terms] OR "policy"[All Fields] OR "policies"[All Fields] OR "policy s"[All Fields] OR "guideline*"[All Fields]
25	design OR deliver*	
26	allocat* OR priorit*	
27	Care	
28	policy OR guideline*	
29	or/24-28	
30	7 and 14 and 23 and 29	
31	7 and 20 and 23 and 29	

FILE 3: PUBLICATION SCHEME SEARCH STRATEGY

The publication scheme search focused on case study hospital Trusts. The focus of the search was the '*How we make decisions'* and *'Our policies and procedures'* sections of the Trust's Publication Scheme. As with the review, sources listed in the publication scheme were excluded if either:

a. they were dated before April 29th, 2020; or
b. their focus and content was on a period prior to April 29th, 2020 (for example an annual report for a financial year to 31st March);

For sources included, a high-level review was then carried out to identify any references to policies or other documents of interest (for example supporting documents or reports prepared for board meetings). The high-level review of included documents was carried out by CR by searching sources for reference to the following terms:

- Covid, Covid-19, coronavirus, SARS-CoV-2, Severe Acute Respiratory Syndrome or pandemic; AND
- Service or care design or delivery, allocation or priority policy, guideline, guidance or framework; OR
    - For paediatric services: Paediatric/pediatric, child/children, critical care, intensive care, acute care, surgery, operation, operating theatre.
    - For maternity services: Maternity, pre-natal, inter-partum, post-natal, perinatal, labour, pregnancy, obstetrics, birth or midwife.

For any sources not accessible through the Trust's publication scheme, Freedom of Information requests were submitted.

FILE 4: THEMATIC ANALYSIS OF PUBLICATION SCHEME CASE STUDY

Publication scheme class	Type of document	Date	Title of document	Themes identified	Sub themes identified
How we make decisions	Board meeting: supporting paper	June 2020	Covid-19 Pandemic – Trust Infection Prevention & Control Response	Respect	collaborative and agile working, patient involvement - eg re-considering place of birth preferences in the context of pressure on emergency ambulance transfer
				Recognising harms and balancing against benefits (physical, psychological, social and economic) – proportionality	staff, patient and visitor safety; testing procedures, agile working, telemedicine, responsiveness - nb availability of abortion medicines at home (no context to this but refs statutory change)
				Reciprocity	Staff expected to take care of their own health
				Accountability	Clear presentation of decisions, rationale, longer term changes to SOP etc.
How we make decisions	Board meeting: supporting paper	June 2020	Update on Covid-19 related Equality Issues	Respect	Involvement - staff and patients to engage in commms around their care and any specific vulnerabilities identified; collaborative working with staff reps, patient groups etc
				Recognising harms etc.	Safety of staff, safety of patients (physical, social, mental wellbeing; specific disadvantages considered - eg non-english speakers; forward planning to mitigate against widening of inequalities
				Reciprocity	Mutual exchange, consideration of social, physical and BAME risk factors

				Fairness<br><br>Accountability	Reducing health inequalities, equality impact assessments (EIAs) on all decisions,<br><br>specific governance decisions, implementation detail (eg EIAs), sharing information and clarity of lines of responsibility.
How we make decisions	Board meeting: supporting paper	June 2020	Safeguarding Service Provisions during COVID: Practice-focused document setting out safeguarding practice during Covid - specific to maternity services	Respect<br><br>Recognising harms etc<br><br>Reciprocity<br><br>Fairness<br><br>Accountability	Organised around creating safe spaces for disclosures - eg routine question added during a scan when partner is not present;<br><br>changing ways of working to ensure awareness of abuse is highlighted in practice, focus on patient safety, collaborative working (other agencies - medical and legal),<br><br>Focus is reduction of patient risk<br><br>Everyone matters equally, reduction of social inequalities, disporportionate impact of Covid on this at risk group (NB impact of domestic abuse on staff is also noted<br><br>Built into reporting and governance procedures
