# Supplementary material for: Which ethical values underpin England’s National Health Service reset of paediatric and maternity services following COVID-19: a rapid review
Source: BMJ Open. 2021 Jun 8;11(6):e049214. doi: 10.1136/bmjopen-2021-049214 (PMC8189755; doi:10.1136/bmjopen-2021-049214)
Supplement: Author's manuscript [file bmjopen-2021-049214.draft_revisions.pdf]

BMJ Open

BMJ Open is committed to open peer review. As part of this commitment we make the peer review history of every article we publish publicly available.

When an article is published we post the peer reviewers' comments and the authors' responses online. We also post the versions of the paper that were used during peer review. These are the versions that the peer review comments apply to.

The versions of the paper that follow are the versions that were submitted during the peer review process. They are not the versions of record or the final published versions. They should not be cited or distributed as the published version of this manuscript.

BMJ Open is an open access journal and the full, final, typeset and author-corrected version of record of the manuscript is available on our site with no access controls, subscription charges or pay-per-view fees (http://bmjopen.bmj.com).

If you have any questions on BMJ Open's open peer review process please email info.bmjopen@bmj.com

BMJ Open

Which ethical values underpin England's National Health Service reset of paediatric and maternity services following Covid-19: a rapid review.

Journal:	*BMJ Open*
Manuscript ID	bmjopen-2021-049214
Article Type:	Original research
Date Submitted by the Author:	19-Jan-2021
Complete List of Authors:	Chiumento, Anna; University of Liverpool Faculty of Health and Life Sciences, Institute of Population Health Sciences<br>Baines, Paul; University of Warwick, Warwick Medical School<br>Redhead, Caroline; University of Liverpool Faculty of Health and Life Sciences, Institute of Population Health Sciences<br>Fovargue, Sara; Lancaster University, Law School<br>Draper, Heather; University of Warwick, Warwick Medical School<br>Frith, Lucy; University of Liverpool, Institute of Population Health Sciences
Keywords:	MEDICAL ETHICS, COVID-19, Health policy < HEALTH SERVICES ADMINISTRATION & MANAGEMENT, Protocols & guidelines < HEALTH SERVICES ADMINISTRATION & MANAGEMENT, QUALITATIVE RESEARCH

SCHOLARONE™
Manuscripts

I, the Submitting Author has the right to grant and does grant on behalf of all authors of the Work (as defined in the below author licence), an exclusive licence and/or a non-exclusive licence for contributions from authors who are: i) UK Crown employees; ii) where BMJ has agreed a CC-BY licence shall apply, and/or iii) in accordance with the terms applicable for US Federal Government officers or employees acting as part of their official duties; on a worldwide, perpetual, irrevocable, royalty-free basis to BMJ Publishing Group Ltd ("BMJ") its licensees and where the relevant Journal is co-owned by BMJ to the co-owners of the Journal, to publish the Work in this journal and any other BMJ products and to exploit all rights, as set out in our licence.

The Submitting Author accepts and understands that any supply made under these terms is made by BMJ to the Submitting Author unless you are acting as an employee on behalf of your employer or a postgraduate student of an affiliated institution which is paying any applicable article publishing charge ("APC") for Open Access articles. Where the Submitting Author wishes to make the Work available on an Open Access basis (and intends to pay the relevant APC), the terms of reuse of such Open Access shall be governed by a Creative Commons licence – details of these licences and which Creative Commons licence will apply to this Work are set out in our licence referred to above.

Other than as permitted in any relevant BMJ Author's Self Archiving Policies, I confirm this Work has not been accepted for publication elsewhere, is not being considered for publication elsewhere and does not duplicate material already published. I confirm all authors consent to publication of this Work and authorise the granting of this licence.

For peer review only

Which ethical values underpin England's National Health Service reset of paediatric and maternity services following Covid-19: a rapid review.

Authors:

- Anna Chiumento (corresponding author): Institute of Population Health Sciences, University of Liverpool, Liverpool L69 3BX, United Kingdom; ORCID ID: 0000-0002-0526-0173; Anna.Chiumento@liverpool.ac.uk
- Paul Baines: Warwick Medical School, University of Warwick, Coventry CV4 7AL United Kingdom; ORCID ID: 0000-0001-9045-4054; Paul.Baines@warwick.ac.uk
- Caroline Redhead: Institute of Population Health Sciences, University of Liverpool, Liverpool L69 3BX, United Kingdom; C.A.B.Redhead@liverpool.ac.uk
- Sara Fovargue: Law School, Lancaster University, Lancaster, United Kingdom LA1 4YW; s.fovargue@lancaster.ac.uk
- Heather Draper: Warwick Medical School, University of Warwick, Coventry CV4 7AL United Kingdom; ORCID ID: 0000-0002-0020-4252; H.Draper@warwick.ac.uk
- Lucy Frith: Institute of Population Health Sciences, University of Liverpool, Liverpool L69 3BX, United Kingdom; ORCID ID: 0000-0002-8506-0699; L.J.Frith@liverpool.ac.uk

ABSTRACT (300 words)

Objective: To identify ethical values guiding decision-making in restarting non-Covid-19 paediatric surgery and maternity services in the National Health Service (NHS).

Design: A rapid review of academic and grey-literature sources from 29th April 2020 to *[final date]*, covering the resumption of non-urgent, non-Covid-19 healthcare. Sources were thematically synthesised against an adapted version of the UK Government's Pandemic Flu Ethical Framework to identify underpinning ethical principles. The strength of normative engagement and the quality of the sources were also assessed.

Setting: NHS maternity and paediatric surgery services in England.

Results: Searches conducted 8th September - 12th October 2020 identified 37 sources meeting inclusion criteria. Themes that arose include: staff safety; collaborative working – including mutual dependencies across the healthcare system; reciprocity; and inclusivity in service recovery, for example by addressing inequalities in service access. Embedded in the theme of staff and patient safety is embracing of new ways of working, such as the rapid roll out of telemedicine. On assessment, many sources did not explicitly consider how ethical principles might be applied or balanced against one-another. Weaknesses in the policy sources included a lack of public and user involvement, and the absence of criteria for monitoring and evaluation.

Conclusions: Our findings suggest that relationality is a prominent ethical principle informing resetting NHS non-Covid-19 paediatric surgery and maternity services. This is explicit in sources highlighting the ethical importance of seeking to minimise disruption to caring and dependent relationships, whilst simultaneously attending to public safety. Engagement with ethical principles was *ethics-lite,* with sources mentioning principles in passing rather than explicitly applying them. This leaves decision-makers and healthcare practitioners without an operationalisable ethical framework to apply to difficult reset decisions, and risks inconsistencies. We recommend further research to confirm or refine the usefulness of the initial reset phase ethical framework developed in our analysis.

ARTICLE SUMMARY

Strengths and limitations of this study:

For peer review only - http://bmjopen.bmj.com/site/about/guidelines.xhtml

- This is the first review to identify the ethical principles guiding decision-making in maternity and paediatric services as England's NHS recommences non-urgent, non-covid-19 healthcare during the pandemic.
- We conducted a rigorous rapid review of sources from policy, academic and grey literature databases.
- Our approach to qualitative synthesis and appraisal of sources against the AGREE-II tool identified areas where ethical guidelines and policies lack clarity and fail to implement patient and public involvement.
- Methodological tensions are present in the use of our coding framework that is based on the 2017 UK Government Pandemic Flu Ethical Framework, and adapted according to two policy sources that met our inclusion criteria.
- An initial Reset Phase Ethical Framework has arisen out of our inductive qualitative synthesis of sources for others to apply and refine.

For peer review only

INTRODUCTION

The Covid-19 pandemic is causing far-reaching consequences for health systems worldwide. In England, the response to the sudden demand for critical care services was to reorient clinical capacity. Many non-urgent services were suspended, and staff and resources redeployed to acute care (1, 2). The pandemic's impact upon routine healthcare has been severe. For example, in England a backlog in areas such as cancer diagnosis and elective surgeries accumulated during the first quarter of 2020 (3, 4). In April 2020, the UK Government declared that non-Covid-19 clinical services **must** resume alongside the capacity for subsequent waves of Covid-19 (5). This created a unique 'reset' context in which it is critical to consider which ethical values *should* underpin decisions about how to reset health services (6). To inform this, we conducted a rapid review of policy, practice and academic sources to identify which ethical values are underpinning reset decision-making in maternity care and paediatric surgery in England.

Our review asked: which ethical values (explicitly or implicitly) guided decision-making in non-Covid-19 paediatric surgery (critical/intensive care admissions, surgery, hospital discharge, and aftercare) and maternity services (pre-natal, intrapartum, and post-partum care) during the initial NHS reset in England? We focussed on maternity and paediatric services because professional and patient organisations have highlighted adverse impacts on these areas due to measures to respond to Covid-19 infections (7-10), presenting clear ethical challenges. Maternity services cannot be suspended, and restrictions on accompanying family and carers may have profound effects. We focussed on restarting paediatric surgery because of clear ethical conflicts in the suspension of elective paediatric services even though children are, on the whole, relatively unscathed by Covid-19; and because the secondary effects of the pandemic may have a greater impact on children (11).

The pandemic, with emerging evidence and uncertain outcomes, rapid adjustments to healthcare policies and practices – both for the acute and now the reset phase - and uncertainties around personal risk, has created a particularly challenging decision-making context. The ethical values guiding the resumption of non-Covid health services are likely to differ from the everyday ethical frameworks relied upon prior to the pandemic. The acute phase of the UK's Covid-19 response has been guided by the Pandemic Flu Ethical framework (12), which reorients decision-making from an individualised to a more public health ethics orientated approach (13, 14). This ethical framing recognises the relational context of decision-making (15), emphasising mutual dependencies. Notably, the Covid-19 pandemic has disproportionately affected certain social groups (16), including vulnerable older people (17), those with disabilities (18) and Black, Asian and minority ethnic (BAME)

communities (19); thus, spotlighting structural inequalities and intersectionalities. It has been proposed that making decisions about healthcare delivery in this context should foreground ethical values such as solidarity (20, 21), reciprocity, and fairness. We aim to identify which ethical values have underpinned decisions about how to reset health services in England (6). This is an important first step in providing a framework for clinicians and healthcare decision-makers specific to the reset period (22).

METHODOLOGY

We adopted a rapid review methodology appropriate to addressing urgent demands for synthesised evidence (23), conducting a qualitative thematic synthesis (24) following the ENTREQ guidelines (25 - see Appendix X for completed ENTREQ checklist). The protocol guided a comprehensive yet pragmatic approach to the searches, screening, analysis, and appraisal of sources (see supplementary file 1).

Inclusion and exclusion criteria

We included sources: (a) developed to guide non-Covid-19 paediatric surgery and maternity services, or (b) that discussed the application of ethical values to paediatric surgery and maternity services in England during the reset phase. The reset phase commenced on April 29th 2020, the day NHS services were instructed to prepare delivery of non-Covid-19 surgical services (5), and remains ongoing. Broadly, the reset requires that NHS Trusts:

- resume all non-urgent services incorporating revised Covid-19 infection prevention and control measures;
- prepare for, and manage, second waves or recurrent waves of Covid-19 infections;
- embrace opportunities to reconfigure health services (e.g. accelerating tele-medicine).

Hence, non-covid-19 services are experiencing a 'reset', rather than simply restarting.

Accordingly, our inclusion criteria were: sources published after 29th April 2020, relating to non-Covid-19 paediatric and maternity services in the NHS in England, discussing decision-making with implicit or explicit reference to ethics, and written in English. We took an inclusive approach to data sources which met the inclusion criteria if they were national (UK-wide and applicable to England), NHS Trust, or local policies and directives; guidelines or statements from professional bodies; working papers or committee reports; evidence reviews; primary qualitative or quantitative research; peer-reviewed commentaries; or grey literature discussing experiences of paediatric or maternity services in England during the reset phase.

Electronic search strategy

Searches were conducted between 8th September and 12th October 2020 by AC and PB *[For reviewers: dates and results to be updated following additional searches immediately prior to publication]*. For academic sources, we searched the bibliographic databases PubMed and PubMed LitCOVID, and clearing houses of Covid-19 related research, including the EPPI Centre Living Map of Covid-19 evidence (26) and Evidence Aid. Recognising the broad scope of our review question, we also searched grey literature sources including websites of UK professional medical bodies (e.g. the Academy of Royal Colleges, and NICE) and clearing houses of Covid-19 sources, such as the Health Foundation Covid-19 Policy Tracker (27). Additional grey literature and academic websites identified during the search dates were included in an effort to achieve completeness (e.g. 28 Covid-19 resources).

We developed MeSH terms that were piloted and refined on PubMed (see supplementary file 1). Where search engines did not facilitate MeSH terms, we selected keywords from the list of terms: for example, "paediatric", "maternity", or "covid-19". For websites where searching was not possible (e.g. 29), a manual review of relevant website sections was undertaken. All grey literature search

For peer review only

results were documented in excel spreadsheets or word documents, and bibliographic database searches in EndNote.

Publication scheme and Freedom of Information requests

To complement the electronic searches, we used the Freedom of Information Act 2000 (FOIA (30)) with NHS England Trusts, including those with Clinical Ethics Committees. FOIA imposes two main duties on public authorities: to proactively publish information in a 'publication scheme' (31), and to respond to requests for information. We focused on sources such as policies, decision-making tools, Trust board papers and minutes that detailed approaches to ethical decision-making guiding maternity and paediatric services during the reset period. The publication scheme review addressed two classes of information: *'How we make decisions'* and *'Our policies and procedures'.* Included documents were read in full and coded against the coding framework by CR (see supplementary file 2). This paper briefly reports a case study example of the publication scheme review.

Screening

Sources were reviewed and duplicates removed before combining results. All were double screened based on title and abstract, where available. Where unavailable, or when undecided, full text review was undertaken. AC, PB, LF, CR and SF screened sources, with HD resolving conflicts in double screening decisions. Papers were categorised against a 0-3 scale, where: 0: not included; 1: included - identifies approach to decision-making; 2: included - identifies what decision has been made; and 3: included – provides justification for decision(s) taken. Where a source met multiple screening categories, all were identified. This categorisation approach sought to provide an initial sense of the depth of sources to inform full-text analysis. Grey literature screening was conducted in a shared excel spread sheet, and for academic sources using Rayyan software (32).

Data analysis

In order to conduct a thematic synthesis of sources, we developed a coding framework for the reset phase. This was based on the Pandemic Flu Ethical Framework (12) adapted according to two interlinked guidance documents: *"Third phase of the NHS response to Covid",* a letter issued by the NHS Chief Executive and Chief Operating Officer to all NHS Trusts (33), and *"Five Principles for the next phase of the Covid-19 response"*, developed by a coalition of UK health and social care charities (34). The 2017 framework provides a checklist to encourage consideration of the full range of ethical principles in decision-making processes, to guide decisions during a pandemic. We adapted the 2017 framework because it was clear that the reset phase may require a different approach to the acute phase. As part of this adaptation, we reduced the Pandemic Flu Ethical Framework (e.g. removing the principle of "flexibility", which was viewed as a sub-domain of "minimising harms and balancing against benefits"), and adjusted sub-domains according to how they were operationalised in these two guidance documents (see table 1 for the reset phase coding framework). This adaptation reduced the overlap between principles and sub-domains for application as a coding framework. The resulting framework was iteratively refined through data analysis, as described in the results. Inductive coding involved reading each document and coding against the ethical principles and sub-domains in the coding framework, alongside a 3-5 line summary of the key points from each document and, where relevant, identifying quotes.

We acknowledge that our approach raises a methodological tension as our coding framework draws on two sources relevant to the review, but which were excluded from it. It was, however, justified given the lack of an overarching ethical framework tailored to the reset phase, and the need for a coding framework that reflects the ethical specificities of this phase. We consider this further in the discussion.

For peer review only

TABLE 1: Reset phase coding framework (adapted from the Ethical Framework in the UK Government's Pandemic Flu Policy (12)):

Ethical principle (from Pandemic Flu Ethical Framework)	Adapted sub-domain (based on NHS letter and National Voices Five Principles)
**Respect**	Involvement (i.e. right to express views on matters affecting them, engaging those affected by decisions)
	Respecting choices about personalised care (best interests of person as a whole)
	Collaborative working / engagement (organisational coordination; NHS volunteer scheme, clinical teams, CCGs, local authorities; co-production with voluntary sector, patient orgs etc)
**Recognising harms & balancing against benefits (physical, psychological, social & economic) - proportionality**	Recover operation of healthcare (inc. addressing backlog of care needs, resuming home visits for vulnerable / shielding where appropriate)
	Safety of NHS staff (physical, psychological, systemic inequalities, flexible working)
	Embrace new ways of working (e.g. telemedicine, home visits etc)
	Enhance crisis responsiveness (second wave)
	Accelerate preventative programmes (obesity reduction, seasonal flu, outreach to marginalised groups)
	Responsiveness (adapt plans to new circumstances / information)
**Reciprocity**	Concept of mutual exchange: take responsibility for own behaviour, reduce others expose others to risks
	Protect those at risk of C19 (physically, socially, BAME etc)
**Fairness**	Inclusivity in service recovery (e.g. barriers or access needs, support those with unequal access to care)
	Patient prioritisation (to address backlog i.e. clinical urgent / longest waiting etc)
	Reduce health inequalities (social inequalities & social determinants of health)
	Everyone matters equally & weighted equally in policies & any disproportionate impact on one particular group is accounted for
**Accountability**	Transparency (i.e. document decisions, clarity of who is responsible for decisions, governance arrangements, assess against milestones, sharing information to help others)

Alongside our thematic synthesis, we assessed the extent to which ethical principles were identified, operationalised, and balanced against one another using a 1-3 scale where: (1) ethical principle(s) inferred or mentioned but not clearly applied; (2) ethical principle(s) identified and application described; and (3) ethical principle(s) operationalised, i.e. discussed in-depth, including balancing against other principles. This scoring system was an adaptation of our protocol: we had intended to apply the 'review of reasons' approach, but the non-normative nature of the majority of sources rendered this approach unsuitable. Data analysis was led by AC, with PB, CR, SF and LF double coding and scoring 16 sources. Following double coding, the team shared analysis, providing a coding check and discussing emerging findings.

Policy sources (including professional guidelines) were appraised for quality using an adapted version of the AGREE-II instrument (35) reduced to 7 core questions (see table 3). In selecting the quality appraisal questions, we considered the standards that could be anticipated in guidelines for which an

For peer review only - http://bmjopen.bmj.com/site/about/guidelines.xhtml

evidence-base was emerging, and where rapid policy and practice decisions were required (36). Appraisal was conducted independently by AC, PB, SF and CR, drawing upon the criteria defined in the AGREE-II Users Manual (37), which includes scoring of 1-7, where: 7: strongly agree (the full criteria are met); 2-6: reporting does not meet the full criteria (i.e. lacks completeness or quality of reporting); and 1: strongly disagree (no information, poor reporting of the criteria, or the authors state that criteria were not met).

RESULTS

We present the results of searches, screening, the characteristics of included sources, and the data analysis. We also separately present a case study example of the publication scheme review from one NHS Trust. To date, no FOI responses providing relevant materials have been received.

Academic and grey-literature searches identified 12,307 sources (6,401 and 5,906 respectively). After removing duplicates, 11,876 results were screened, with 11,571 excluded as not relevant. 305 sources were assessed for eligibility by title and abstract or, where necessary, full-text screening. Of these, 199 were excluded as being outside the review scope, and upon full text review a further 27 sources were excluded. Therefore, searches identified 37 sources for analysis (see Figure 1).

FIGURE 1: PRISMA flow diagram of searches

Table 2 presents key characteristics of the 37 included sources, which include professional guidelines (n=27), Government policy statements/letters (n=3), academic papers (n=3), a report of patient engagement and of implementing professional guidelines, a briefing paper, and a blog post (n=1 of each). Fifteen sources covered all areas of clinical care, 15 focused on maternity services, 6 on paediatric services, and 1 on consent for surgery. The sources covered England or the UK, with some containing Trust-specific case studies. Finally, some sources cross-referenced one another; for example, the Academy of Medical Royal Colleges (38) has accompanying sources focussing on specific areas, such as staff support (39).

For peer review only

TABLE 2: Key characteristics of sources

Title	Reference	Publication type (policy, report, press release, briefing, professional guideline, peer reviewed article, commentary, decision-support tool / framework, blog)	Date of publication (DD/MM/YY or MM/YY)	Population (Maternity, Paediatrics, or all clinical specialities)	Source scope (national, regional, trust, hospital)
**Grey literature sources**					
Principles for reintroducing health services - COVID-19	(38)	Professional guideline	May-20	All	National
Covid-19. Effects on health from non-Covid-19 conditions and moving forward to deliver healthcare for all	(40)	Professional guideline	May-20	All	National
Preparing for COVID-19 surges and winter	(41)	Professional guideline	Jul-20	All	National
Reset, restore and recovery: staff support	(39)	Professional guideline	Jun-20	All	National
Health Protection: Public and professional responsibilities	(42)	Professional guideline	11/07/2020	All	National
Reset, restore and recovery: medical education and training	(43)	Professional guideline	Jun-20	All	National
Reset, restore and recovery: equality	(44)	Professional guideline	Jun-20	All	National
Second phase of NHS response to COVID-19	(45)	Policy (letter)	29/04/2020	All	National
Operating framework for urgent and planned services within hospitals: all emergency patients to be tested on admission and elective patients to isolate for 14 days prior to admission	(46)	Policy	05/20 ?	All	National
Second phase of NHS response to COVID-19 for cancer services	(45)	Policy (letter)	08/07/2020	All	National
WRES briefing for board and COVID-19 emergency preparedness, resilience and response (EPRR) membership in the NHS	(47)	Briefing	24/06/2020	All	National
COVID-19: Guidance for the remobilisation of services within health and care settings, infection prevention and control recommendations	(48)	Public Health England Guidance	20/08/2020	All	National

For peer review only - http://bmjopen.bmj.com/site/about/guidelines.xhtml

Delivering a paediatric elective surgery service during the COVID-19 pandemic	(49)	Implementation of NICE guidance	27/07/2020	All	National
COVID-19: guidance for planning paediatric staffing and rotas	(50)	Professional guidance	10/07/2020	Paediatrics	National
COVID-19 & Us: views from RCPCH & Us	(51)	RCPCH Engagement	04/11/2020	Paediatrics	National
Ethics framework for use in acute paediatric settings during COVID-19 pandemic	(52)	Professional guidance	01/09/2020	Paediatrics	National
National guidance for the recovery of elective surgery in children	(53)	Professional guidance	09/11/2020	Paediatrics	National
Reset, Restore, Recover - RCPCH principles for recovery	(54)	Professional guidance	19/05/2020	Paediatrics	National
It is right to restart services, but we must do so in a safe way	(55)	Blog	07/06/2020	All	National
Antenatal Care for women without suspected or confirmed COVID-19 and living in a symptom free household	(56)	Professional guidance	14/08/2020	Maternity	National
RCM Briefing on Re-introduction of visitors to Maternity Units across the UK during the COVID-19 pandemic	(57)	Professional guidance	15/07/2020	Maternity	National
RCM Clinical Briefing Sheet: guidance for midwifery services on ‘freebirth’ or ‘unassisted childbirth’ during the COVID-19 pandemic	(58)	Professional guidance	30/04/2020	Maternity	National
Guidance for the provision of midwife-led settings and home birth in the evolving coronavirus (COVID-19) pandemic	(59)	Professional guidance	22/05/2020	Maternity	National
Equality essentials: Appropriate risk assessment during the current pandemic	(60)	Professional guidance	May-20	Maternity	National
COVID-19 impact on Black, Asian and Minority ethnic (BAME) women	(61)	Professional guidance	15/07/2020	Maternity	National
Principles for the testing and triage of women seeking maternity care in hospital settings during the Covid-19 pandemic: a supplementary framework for maternity healthcare professionals	(62)	Professional guidance	10/08/2020	Maternity	National
Guidance for antenatal and postnatal services in the evolving coronavirus (COVID-19) pandemic	(63)	Professional guidance	19/06/2020	Maternity	National

For peer review only

Antenatal care for women with current suspected or confirmed COVID-19 or with a member of their household with suspected or confirmed COVID-19	(64)	Professional guidance	24/07/2020	Maternity	National
Domestic Abuse	(65)	Professional guidance	13/05/2020	Maternity	National
Bereavement Care in Maternity Services During COVID-19 pandemic	(66)	Professional guidance	14/07/2020	Maternity	National
Postnatal Care for women with suspected or confirmed COVID-19	(67)	Professional guidance	14/08/2020	Maternity	National
Virtual Consultations	(68)	Professional guidance	24/07/2020	Maternity	National
Restarting planned surgery in the context of the COVID-19 pandemic	(69)	Professional guidance	01/05/2020	All	National
Delivering midwifery intrapartum care where local COVID-19 escalation protocols are required to be enacted	(70)	NICE guidance	20/07/2020	All	National
**Academic sources**					
Implications for the future of obstetrics and Gynaecology following the COVID-19 pandemic: a commentary	(71)	Commentary		Maternity	National
Sustaining quality midwifery care in a pandemic and beyond	(72)	Review article	25/05/2020	Maternity	National
How should surgeons obtain consent during the Covid-19 pandemic?	(73)	BMJ Views and Reviews	30/06/2020	All surgery	National

For peer review only

Table 3 summarises the assessment of 33 policy sources/professional guidelines against the AGREE-II tool. Sources scored highest for clarity of the guideline objective (15 scored seven, and nine scored six) and easily identifiable key recommendations (15 scored seven). Favourable scores were achieved for the involvement of professional groups (seven scored seven, and 13 between four and five). Conversely, on seeking views of the target population, 18 sources scored one, with two scoring seven; and on whether the guideline presented monitoring and/or auditing criteria, 20 sources scored one. When assessing whether there was an explicit link between the recommendations and supporting evidence, 18 scored one, two scored seven and one scored six. Finally, all sources scored one for whether the competing interests of members of the guideline development group had been recorded and addressed.

For peer review only

TABLE 3: AGREE-II assessment of 33 policy guideline sources

Title	Reference	AGREE-II Questions (domain in brackets)						
		The guideline objective is specifically described (D1)	The guideline development group includes individuals from all relevant professional groups (D2)	The views & preferences of the target population have been sought (D2)	There is an explicit link between the recommendations and the supporting evidence (D3)	Key recommendations are easily identifiable (D4)	The guideline presents monitoring and/or auditing criteria (D5)	Competing interests of the guideline development group members have been recorded & addressed (D6)
Principles for reintroducing health services - COVID-19	(38)	7	5	1	4	7	1	1
Covid-19. Effects on health from non-Covid-19 conditions and moving forward to deliver healthcare for all	(40)	6	4	3	3	7	1	1
Preparing for COVID-19 surges and winter	(41)	7	4	3	3	7	1	1
Reset, restore and recovery: staff support	(39)	7	4	1	1	7	1	1
Health Protection: Public and professional responsibilities	(42)	7	4	1	1	7	1	1
Reset, restore and recovery: medical education and training	(43)	7	4	3	2	7	1	1
Reset, restore and recovery: equality	(44)	7	4	1	2	7	1	1
Second phase of NHS response to COVID-19'	(5)	7	5	1	3	7	4	1
Operating framework for urgent and planned services	(46)	2	1	1	1	7	2	1

For peer review only

within hospitals: all emergency patients to be tested on admission and elective patients to isolate for 14 days prior to admission								
Second phase of NHS response to COVID-19 for cancer services	(45)	1	3	1	2	5	1	1
WRES briefing for board and COVID-19 emergency preparedness, resilience and response (EPRR) membership in the NHS	(47)	4	1	1	3	5	1	1
COVID-19: Guidance for the remobilisation of services within health and care settings, infection prevention and control recommendations	(48)	5	7	1	4	5	1	1
Delivering a paediatric elective surgery service during the COVID-19 pandemic	(49)	7	7	7	7	5	3	1
COVID-19: guidance for planning paediatric staffing and rotas	(50)	7	1	1	1	6	1	1
COVID-19 & Us: views from RCPCH & Us	(51)	7	5	7	7	7	1	1
Ethics framework for use in acute paediatric settings during COVID-19 pandemic	(52)	7	7	1	5	7	3	1
National guidance for the recovery of elective surgery in children	(53)	7	7	5	7	7	4	1

For peer review only

Reset, Restore, Recover - RCPCH principles for recovery	(54)	7	1	1	3	7	1	1
Antenatal Care for women without suspected or confirmed COVID-19 and living in a symptom free household	(56)	5	1	1	5	7	1	1
RCM Briefing on Re-introduction of visitors to Maternity Units across the UK during the COVID-19 pandemic	(57)	4	1	1	3	3	1	1
RCM Clinical Briefing Sheet: guidance for midwifery services on 'freebirth' or 'unassisted childbirth' during the COVID-19 pandemic	(58)	5	1	1	4	3	1	1
Guidance for the provision of midwife-led settings and home birth in the evolving coronavirus (COVID-19) pandemic	(59)	6	6	1	5	3	1	1
Equality essentials: Appropriate risk assessment during the current pandemic	(60)	5	3	3	3	5	2	2
COVID-19 impact on Black, Asian and Minority ethnic (BAME) women	(61)	6	4	2	5	4	2	1
Principles for the testing and triage of women seeking maternity care in hospital settings during the Covid-19 pandemic: a supplementary	(62)	6	3	2	5	5	3	1

For peer review only

framework for maternity healthcare professionals								
Guidance for antenatal and postnatal services in the evolving coronavirus (COVID-19) pandemic	(63)	6	7	2	5	5	3	1
Antenatal care for women with current suspected or confirmed COVID-19 or with a member of their household with suspected or confirmed COVID-19	(64)	6	5	2	6	6	2	1
Domestic abuse	(65)	6	3	3	4	4	2	1
Bereavement Care in Maternity Services During COVID-19 pandemic	(66)	6	4	6	7	3	1	1
Postnatal Care for women with suspected or confirmed COVID-19	(67)	5	7	5	6	4	1	1
Virtual Consultations	(68)	7	5	5	7	6	4	1
Restarting planned surgery in the context of the COVID-19 pandemic	(69)	6	7	1	1	7	1	1
Delivering midwifery intrapartum care where local COVID-19 escalation protocols are required to be enacted	(70)	7	5	1	6	5	1	1

For peer review only

Table 4 summarises the qualitative thematic synthesis of all 37 sources, highlighting the frequency of coding to each sub-domain, and scores for the operationalisation of ethical principles.

For peer review only

TABLE 4: Thematic analysis of sources

**THEMATIC ANALYSIS**		
**Principles**	**Sub-domains**	**References**
**Respect**	Involvement	(38, 40, 41, 43, 46, 47, 51-54, 57-59, 61-64, 66-68, 71, 73, 74)
	Respecting choices about personalised care	(38, 52-54, 58, 59, 61, 63, 64, 66, 68, 73)
	Collaborative working / engagement	(5, 38, 40, 41, 46-50, 53, 54, 56-59, 63, 65-67, 69-71, 74)
**Recognising harms & balancing against benefits (physical, psychological, social & economic) - proportionality**	Recover operation of healthcare	(5, 38-41, 44, 46, 49-52, 54-57, 59, 62-67, 69, 73, 74)
	Safety of NHS staff	(5, 38-40, 42-44, 46-50, 53-55, 57, 59-64, 67-72, 74)
	Embrace new ways of working	(5, 38, 41, 47-49, 51, 53, 54, 56, 58, 59, 61-64, 66, 68, 71, 72)
	Enhance crisis responsiveness	(5, 38, 39, 41, 52, 53, 55)
	Accelerate preventative programmes	(5, 40, 41, 61, 71)
	Responsiveness	(47, 50-54, 56, 58, 59, 62-64, 66, 67, 69, 70)
	Patient safety	(40, 42, 46, 49, 52, 53, 55, 56, 58, 59, 61-65, 72, 73)
**Reciprocity**	Mutual exchange	(41, 42, 48, 50, 53, 57, 61, 64)
	Protect those at risk of Covid-19	(5, 38, 40-42, 44, 46-51, 53, 56, 57, 59, 60, 62-64, 67, 69, 72)
**Fairness**	Inclusivity in service recovery	(38, 40, 44, 51, 56, 59, 61, 63, 65-69, 71, 74)
	Patient prioritisation	(5, 38, 40, 44, 46, 52, 53, 55, 61, 69)
	Reduce health inequalities	(44, 47, 51, 54, 56, 60, 61, 63, 65, 67, 68, 71)
	Everyone matters equally	(47, 52, 53, 55, 57, 58, 61, 62, 69, 71)
**Accountability**	Transparency	(5, 40, 41, 44, 46-48, 51-54, 57-59, 68)
	Finance	(5, 71)
**JUSTIFICATION OF PRINCIPLES**		
**1**	Principle(s) inferred or mentioned, but not clearly applied	(5, 38-42, 44, 46, 51, 53, 54, 60, 61, 64, 67, 70, 74)
**2**	Application of principle(s) described	(43, 47-50, 55-58, 62, 63, 65, 66, 68, 71, 73)
**3**	Application of principle(s) discussed in-depth, including balancing against other principle(s)	(52, 59, 69, 72)

For peer review only

All sources explicitly referenced or applied the principle of recognising harms and balancing these against possible benefits. The sub-domain of *safety of NHS staff* was most frequently coded, with *recovering the operation of healthcare* and *embracing new ways of working* explicitly identified slightly less frequently. Staff safety was understood broadly, encompassing PPE, testing and isolation protocols, the importance of staff wellbeing (including leave to recover from the first wave of Covid-19), and the importance of ongoing staff training (40, 43, 50, 55). Examples of new ways of working frequently identified telemedicine, an approach that has been effective in remote community maternity care prior to the pandemic (68). Integrating telemedicine was recommended in the context of trusting relationships built through in-person care (63) that involved individualised assessments of patients' characteristics and life circumstances (68), such as the need for interpretation services (56), and confidentiality concerns (51). In the resetting of health services, sources anticipated that routine care would resume in a non-linear way (69); therefore, continuing adaptation to the evolving situation would be required (56). To support this, a number of sources proposed risk management tools and service level models that accounted for impacts upon key areas, such as human resources (53, 56), or sample risk assessments with recommended phases; for example, for reintroducing visitors and sample visiting guidelines (57). One source cautioned against resuming planned healthcare too quickly, citing the time and effort required to reorient people and equipment to routine roles, and the additional demands of patient safety and infection control, citing concerns about PPE and drug supply chains (55).

Respect was another principle frequently explicitly considered, encompassing keeping people informed and respecting personal decisions about care, including acknowledging patients' right to express views on matters affecting them. One source implemented involvement by using patients' experiences of Covid-19 societal lockdown to inform plans for maintaining routine care alongside managing Covid-19 (51). The use of active public health messaging or outreach to involve patients was identified (40, 53, 71) and was added to the coding framework as a sub-domain of respect. Collaborative working was also explicitly referenced, recognising the co-dependency of elements of the health service*: "turning on the tap at one end will not necessarily release the flow at the other — there are multiple taps which need to be released in a sequential fashion"* (40). Sources called for embedding collaboration across hospitals and Trusts through local, regional and national coordination, the redeployment of staff across specialities, accelerated qualification of students, and return of retired staff who had supported human resource capacity during the first wave of Covid-19 (5, 40).

Inclusivity in service delivery was emphasised under the principle of fairness. Barriers to maternity care such as English language abilities, immigration status, and individualised factors - including risk of domestic abuse or history of human trafficking - were identified (61, 65). This sub-domain was frequently considered alongside explicit recognition that everyone matters and should be considered equally in policies. For example: *"…it is important to consider the needs of surgical patients on an equal footing with those receiving care for COVID-19 and other medical diseases"* (69). Some sources also stressed conducting Equality Impact Assessments to ensure rapid adjustments of policies and procedures to address inequalities and meet public duties (5, 57).

Under the principle of reciprocity, the sub-domain of everyone taking actions to protect healthcare workers and patients was explicitly emphasised. Notably, this recognised the increased risks and burdens faced by healthcare staff and those at increased risk of Covid-19 infection and poor outcomes, such as members of BAME communities (41, 44, 47). Finally, accountability was implicitly reflected in the sub-domain of transparency, with explicit reference to documenting decisions (52, 58, 68) and engaging in monitoring, evaluation (53), and research (5, 41). Some sources also underscored

For peer review only

transparency in governance structures and decision-making processes (3), thereby ensuring adherence to the UK Equalities Act 2010.

The analysis led to iterative inductive evolution of the coding framework, adding sub-categories identified in italics in table 5.

For peer review only

TABLE 5: Reset phase coding framework inductively developed through the review (adapted from the UK Government's Pandemic Flu Policy Ethical Framework (12))

Ethical principle (from Pandemic Flu Ethical Framework)	Sub-domain
**Respect**	Involvement (i.e. right to express views on matters affecting them, engaging those affected by decisions, *active communication / outreach including public health messaging*)
	Respecting choices about personalised care (best interests of person as a whole *including decisions in best interests of children and young people*)
	Collaborative working / engagement (organisational coordination; NHS volunteer scheme, clinical teams, CCGs, local authorities, *Nightingale & independent hospitals*; co-production with voluntary sector, patient orgs, etc.)
**Recognising harms & balancing against benefits (physical, psychological, social & economic) - proportionality**	Recover operation of healthcare (including addressing backlog of care needs, resuming home visits for vulnerable / shielding where appropriate; *resources (staffing & spaces / equipment etc.*)
	Safety of NHS staff (physical, psychological, systemic inequalities, flexible working, *meeting staff training needs*)
	Embrace new ways of working (e.g. telemedicine, home visits etc.)
	Enhance crisis responsiveness (second wave)
	Accelerate preventative programmes (obesity reduction, seasonal flu, outreach to marginalised groups)
	Responsiveness (adapt plans to new circumstances / information)
	Patient safety (individualised risk protocols)
**Reciprocity**	Concept of mutual exchange: take responsibility for own behaviour, reduce others expose others to risks
	Protect those at risk of C19 (physically, socially, BAME etc)
**Fairness**	Inclusivity in service recovery (e.g. barriers or access needs, support those with unequal access to care)
	Patient prioritisation (to address backlog i.e. clinical urgent / longest waiting etc)
	Reduce health inequalities (social inequalities & social determinants of health)
	Everyone matters equally & weighted equally in policies & any disproportionate impact on one particular group is accounted for
**Accountability**	Transparency (i.e. document decisions, clarity of who is responsible for decisions, governance arrangements, assess against milestones, sharing information to help others)
	Finance

Scoring sources for their practical usefulness to clinicians highlights that nearly half explicitly identified key ethical principles but failed to offer advice about how they might support decision-making (17 scored one). These sources often made broad statements about core principles, such as patient respect and minimising harms, which were frequently mentioned in relation to infection prevention and control. 16 sources scored two for clearly identifying ethical principles and suggesting how they might be applied; for example, by identifying decision-making support tools (e.g. The Royal College of Midwives (57)). Four sources scored three for their focused, practical suggestions regarding the

For peer review only

application of the identified ethical principles, often balancing them against one another. For example, in the ethical framework for acute paediatric settings, Wilkinson (52) balanced treatment prioritisation against resource constraints, identified decision-making tools, and engaged with case scenarios to illustrate ethical tensions, such as the disruptions to care pathways for children with complex needs.

Publication scheme case study

We present initial findings from one NHS Trust publication scheme review (see supplementary file 3). As with the wider review findings, the Trust board's focus was on patient, staff, and visitor safety, including broad concern with the effects of the Trust's decision-making on service delivery during the reset period. An example from a maternity service was the creation of a safe space for disclosure of domestic violence by making a small, but important, adjustment to Trust Standard Operating Procedures by adding questions to ask when a pregnant person's partner was not present. This example reflects an awareness of patients' increased exposure to domestic violence as a result of lockdown, demonstrating the benefit of paying attention to ethical considerations including inequality and patient safety in a specific decision-making context.

DISCUSSION

Our pragmatic rapid review has identified the ethical principles referenced in published academic and grey decision-making guidance informing the resetting of NHS paediatric surgery and maternity services. A key review outcome is the reset phase ethical framework inductively developed based upon the sources reviewed (Table 5). In this discussion, we focus on two areas of ethical distinctiveness: the ways that relationality was invoked, and the emphasis on equity. We also consider the practical usefulness of the included sources for practitioners applying to concrete situations (75), and outline how the reset ethical framework developed through this review might be operationalised.

Relationality was reflected in numerous ways, anchored in the individual and organisational mutual dependencies and responsibilities that have been starkly highlighted by the Covid-19 pandemic. The ethical importance of attending to the adverse impact of Covid-19 on caring and dependent relationships, seeking to minimise disruption to these as much as possible to meet the needs of patients and family or carers, whilst simultaneously attending to public safety is one example. In our review, the relational context of decision-making was prominent, reflecting family and caring relationships inherent to our areas of focus: birthing partners in maternity care, and parents or carers in paediatric services (52, 66). Explicit steps to minimise harms and maximise staff and patient safety were grounded in risk assessment and infection prevention and control protocols that relied upon reciprocal responsibilities. Reciprocity was also explicitly identified in the additional protections for those at risk of adverse outcomes from Covid-19 due to systematic inequalities and intersectionalities (16). Sources explicitly recognised the importance of balancing infection prevention and control actions to reduce Covid-19 transmission with other risks to healthcare; notably acknowledging the potential emotional impacts for patients attending appointments or giving birth alone. Psychological safety was reflected in explicit calls to attend to the emotional impacts of delivering care in a Covid-19 context and to minimise the risk of staff burnout. Finally, relationality was implicit in inter-organisational collaboration locally, regionally and nationally to coordinate continuity of care, emphasising co-dependencies of different areas of the health service (76). A distinctive focus on health equity was explicit in sources balancing the needs of those with Covid-19 with those requiring routine healthcare. Health equity was also implicitly reflected in calls for pro-active outreach to overcome health inequalities and ensure care was accessed when needed, including public health measures such as immunisation campaigns attending to potential inequalities of access.

For peer review only - http://bmjopen.bmj.com/site/about/guidelines.xhtml

Our assessment of the level of engagement with ethical principles found them to be ‘ethics-lite’. Whilst key principles were referenced, sometimes only in passing, many sources failed to operationalise them. We define operationalisation as applying ethical principles to specific situations, considering how ethical dilemmas that could be predicted to arise might be managed, or offering suggestions as to how, in practice, ethical principles might be balanced against one another. In recognising this, we do not call for prescriptive guidance for every circumstance, but note that guidelines should inform, and constrain, the judgements of those applying them (75), and to achieve this *how* they ought to be operationalised needs to be clear. Guidelines lacking this dimension leave healthcare decision-makers and clinicians without a coherent ethical framework to support decision-making (22), which can result in moral distress (77). Moreover, as Kasaven, Saso, Barcroft*et al.* (71) note: *“Research in psychology has demonstrated that when people are working in stressful situations under pressure of time, with access to extensive yet conflicting information from multiple sources, and when outcomes are uncertain, they tend to make more decisions based on intuition, gut feelings, or heuristics (rules of thumb) rather than on rational thinking (Kahneman, 2011)*" (p.2). The Covid-19 context, with emerging evidence and uncertain outcomes, rapid adjustments to healthcare policies and practices – both for the acute and the reset phase - and uncertainties around personal risk, perfectly reflect the context Kahneman describes. In such situations, it can be difficult for decision-makers and clinicians to interpret and apply broad-brush ethical guidelines to practice, and to do so consistently. A clear ethical framework to underpin decision-making is therefore required (75, 78).

Our reset ethical framework, inductively developed through this review, offers a useful starting point on which to build. Additional research to confirm or further refine its congruence to the decision-making processes of individual Trusts and healthcare providers - embedded within their regional and systemic relationships, and to areas of healthcare beyond paediatric surgery and maternity services, are required. This forms part of our ongoing project activities. Recognising the importance of our review finding that ethical frameworks should be operationalisable, we briefly explain how our reseat ethical framework could be applied in practice. The Pandemic Flu Ethical Framework emphasises *equal concern and respect* as the underpinning principle (79), which is echoed in our review where *fairness*, chiefly that *everyone matters equally and is weighted equally*, has emerged as the underpinning principle. However, our review demonstrates the NHS operational context in the reset phase is ethically distinct. The underpinning principle of fairness must be balanced across considerations such as the impact of delayed care; constraints of infection prevention and control measures; broad mutual inter-dependencies between healthcare providers, patients and the public; and uncertain Covid-19 risks – exacerbated by inequalities and intersectionalities - for healthcare providers and patients. These considerations foreground complex configurations of layers of interdependencies and relationships embedded within healthcare provision in the reset phase. Ethical frameworks may assist decision-makers to navigate this challenging decision-making context. Consequently, in contrast to the UK Chief Medical Officers advice not to produce updated ethical guidance for the Covid-19 pandemic (80), our review indicates that the ethically distinctive Covid-19 healthcare operational context urgently requires a tailored approach (81). We agree with the Scottish Government (82) that the framework should be operationalised to support organisational and individual-level decision-making at national, regional and local levels; for example, through Trust specification (see e.g. 83) and with the pragmatic advice and consultation of Clinical Ethics Committees, and, where relevant, patient involvement groups.

Appraising sources against the AGREE-II tool identified a lack of monitoring and auditing systems for rapidly adjusted policies and practice guidelines, which is concerning given the reported impacts on some areas of patient care. It also showed a lack of public involvement beyond, at best, patient representatives (67), and a lack of transparency around potential competing interests in guideline

For peer review only

development. The Governments' Phase two letter provided Trusts the short timeline of 21 weeks to design their service reset (5). Engagement processes, already time consuming, had to be adapted to online formats. It is, therefore, not surprising that public involvement was lacking. However, in March 2020 NHS England restated the statutory, and ethical, duty to maintain public involvement in decisions about service provision (84), suggesting that this should have taken place. Public involvement is fundamental to public trust in the collective actions of the NHS, and the standards of professional ethical practice of individual health care providers (85-87). This is essential to meeting the NHS Constitution's guiding principle, that "*the NHS is accountable to the public, communities and patients that it serves*" (88). As such, public and patient involvement provides an important moral foundation for difficult ethical decisions in the reset phase.

Our review attended to the maintenance of rigour by including a systematic search strategy where possible and double screening and double coding 25% of sources. Team discussions to develop the coding framework and reflect on emerging findings were also ongoing throughout. We adopted an inclusive approach to grey literature and academic sources, ensuring the relevance of our review to healthcare policy and practice. This was complemented by the publication scheme review, which indicated the application of guidelines to situated Trust-level decision-making. The review rapidity necessarily limited its scope and depth (36), and may not have identified all relevant sources. Time constraints prevented a multiple appraisal of policy sources as recommended for the AGREE-II tool (37), and meant that only CR analysed the publication scheme data. We also faced methodological challenges, notably the tension in developing the coding framework from two sources that met the review inclusion criteria. We believe this tension is acceptable given the inductive and iterative thematic synthesis approach, which led to the inductive development of a revised framework that reflects the distinctive considerations facing decision-makers and clinicians during the reset phase. Finally, the breadth of our review question made the adoption of approaches designed for normative reviews challenging, and resulted in the use of a scoring system that accommodated our review scope.

This review has sought to render explicit the decision-making factors specific to the reset phase, yielding important learning for healthcare policy makers and Trust decision-makers. Our findings suggest that some key ethical and legal duties – such as involvement – have been immediate casualties of the time-pressured decision-making context. We accept there may be significant logistical barriers to achieving meaningful engagement, and that compromises during a crisis may be required (12). However, we recommend that guidance documents are transparent about any lack of involvement and the reasons for this, whilst seeking to re-establish meaningful engagement as quickly as possible. We also recommend that those developing policy and practice guidelines pay attention to their practical application. This will ensure that any normative decision-making is operationalisable in the context in which decision-makers and practitioners are working.

CONCLUSION

This review adds to the rapidly evolving evidence on England's health systems' response to the Covid-19 pandemic, focussing on the normative foundations underpinning the resetting of NHS health services in maternity and paediatric surgery services, alongside a continuing response to the demands of Covid-19. It is important that the government and professional bodies continue to engage with the difficult ethical decisions this requires, and we recommend increased public involvement in this process to build solidarity in supporting the required responses. Our review has found that to date, guidelines and statements developed for this period are ethics-lite and fail to provide an operationalisable ethical framework for decision-makers and healthcare professionals to apply. Addressing this is an important priority as the NHS in England moves further into the reset period, where difficult ethical decisions about how the health services resets will continue to be necessary.

For peer review only

We intend to support this process by publishing our proposed reset ethics framework here. This has been inductively developed based upon the sources included in this review. We continue to refine this framework through our ongoing empirical and conceptual research.

AUTHORS CONTRIBUTIONS STATEMENT

LF, HD, AC, SF and PB designed the rapid review concept and question. All authors contributed to the development of the review protocol, and were involved in various stages of conducting the review, as specified in the paper. All authors were involved in regular team meetings to discuss and reflect upon review conduct and emerging findings. AC led the writing of the paper, with all authors providing review and feedback, and approving the final version for publication.

COMPETING INTERESTS STATEMENT

The authors declare they have no competing interests.

FUNDING STATEMENT

This rapid review is the first phase of the *Everyday and Pandemic Ethics* project (https://www.liverpool.ac.uk/population-health-sciences/departments/health-services-research/key-projects/resetethics/) funded by the UKRI AHRC Covid-19 rapid response call.

DATA SHARING STATEMENT

Additional data available upon reasonable request to the corresponding author.

ACKNOWLEDGEMENTS

The authors thank Dr Rui Hill, University of Liverpool for his helpful guidance on early stages of developing the review protocol and suggestions for review software. We also thank Dr Diego Silva, Melbourne University for his feedback on an earlier draft of this paper as discussant at an Australasian Association of Bioethics in Health and Law workshop in December 2020; and the helpful reflections of other members of the workshop session.

MAIN MANSUCRIPT WORD COUNT: 4,912 words.

REFERENCES

1. Academy of Medical Royal Colleges. NHS Health and Social Care Committee: Delivering core NHS and care services during the pandemic. London, UK: Academy of Medical Royal Colleges; 2020. https://www.aomrc.org.uk/wp-content/uploads/2020/07/200630_NHS_health_social_care_committee_submission.pdf. (Access date: 03.11.2020).
2. ICM Anaesthesia COVID-19. Anaesthesia and critical care: guidance for Clinical Directors on preparation for a possible second surge in COVID-19. London, UK; 2020. https://icmanaesthesiacovid-19.org/anaesthesia-and-critical-care-second-surge-guidance. (Access date: 05.11.2020).
3. National Health Service. Implementing phase 3 of the NHS response to the Covid-19 pandemic. 2020. https://www.england.nhs.uk/wp-content/uploads/2020/08/C0716_Implementing-phase-3-v1.1.pdf. (Access date: 29.09.2020).
4. Gardner T, Fraser C, Peytrignet S. Elective care in England: Assessing the impact of COVID-19 and where next. The Health Foundation; 2020. https://www.health.org.uk/publications/long-reads/elective-care-in-england-assessing-the-impact-of-covid-19-and-where-next. (Access date: 25.11.2020).
5. Stevens S, Pritchard A. Second phase of NHS response to COVID-19. 2020. https://www.england.nhs.uk/coronavirus/wp-content/uploads/sites/52/2020/04/second-phase-of-nhs-response-to-covid-19-letter-to-chief-execs-29-april-2020.pdf. (Access date:

For peer review only

6. NHS Confederation. Health and Social Care Select Committee inquiry on delivering core NHS and care services during the pandemic and beyond: written evidence from the NHS Confederation. 2020. https://www.nhsconfed.org/-/media/Confederation/Files/Public-Affairs/NHS-Confederation-submission-to-inquiry-on-Delivering-Core-NHS-and-Care-Services-during-the-Pandemic.pdf. (Access date: 03.11.2020).
7. McDonald HI, Tessier E, White JM, *et al.* Early impact of the coronavirus disease (COVID-19) pandemic and physical distancing measures on routine childhood vaccinations in England, January to April 2020. Eurosurveillance. 2020;25(19):2000848. https://doi.org/10.2807/1560-7917.ES.2020.25.19.2000848.
8. First 1001 Days Movement. Our call on Government to keep babies safe [press release]. 2020. https://parentinfantfoundation.org.uk/our-call-on-government-to-keep-babies-safe/. (Access date: 10.09.2020).
9. Anonymous. Reflections on Covid restrictions from a pregnant midwife. Make Births Better2020. [25.11.2020]. Available from: https://www.makebirthbetter.org/blog/reflections-on-covid-restrictions-from-a-pregnant-midwife. (Access date: 25.11.2020).
10. Association of Paediatric Anaesthetists of Great Britain and Ireland A. APAGBI position statement regarding the delivery and recovery of Children's surgery during the coronavirus pandemic [press release]. 2020. https://www.apagbi.org.uk/sites/default/files/inline-files/APA%20statement%2015.05.20%20Final.pdf. (Access date: 25.11.2020).
11. UNICEF. COVID-19 and children 2020 [Available from: https://data.unicef.org/covid-19-and-children/. (Access date: 09.01.2021).
12. UK Government. Pandemic Flu. 2017. London, UK. UK Government. https://www.gov.uk/guidance/pandemic-flu. (Access date: 24.06.2020).
13. Baines P, Draper H, Chiumento A, *et al.* COVID-19 and beyond: the ethical challenges of resetting health services during and after public health emergencies. Journal of Medical Ethics. 2020;46(11):715-6. 10.1136/medethics-2020-106965.
14. Berlinger N, Wynia M, Powell T, *et al.* Ethical Framework for Health Care Institutions Responding to Novel Coronavirus SARS-CoV-2 (COVID-19): Guidelines for Institutional Ethics Services Responding to COVID-19. The Hastings Centre; 2020. https://www.thehastingscenter.org/wp-content/uploads/HastingsCenterCovidFramework2020.pdf. (Access date: 25.11.2020).
15. Dove ES, Kelly SE, Lucivero F, *et al.* Beyond individualism: Is there a place for relational autonomy in clinical practice and research? Clinical Ethics. 2017;12(3):150-65. 10.1177/1477750917704156.
16. Horton R. Offline: COVID-19 is not a pandemic. The Lancet. 2020;396(10255):874. 10.1016/S0140-6736(20)32000-6.
17. Public Health England. Disparities in the risk and outcomes of COVID-19. London: Public Health England; 2020. https://assets.publishing.service.gov.uk/government/uploads/system/uploads/attachment_data/file/908434/Disparities_in_the_risk_and_outcomes_of_COVID_August_2020_update.pdf. (Access date: 17.12.2020).
18. Office for National Statistics. Coronavirus and the social impacts on disabled people in Great Britain: September 2020. London: UK Government, . 2020. https://www.ons.gov.uk/peoplepopulationandcommunity/healthandsocialcare/disability/articles/coronavirusandthesocialimpactsondisabledpeopleingreatbritain/september2020. (Access date: 18.01.2021).
19. Public Health England. Beyond the data: understanding the impact of COVID-19 on BAME groups. London: Public Health England; 2020. https://assets.publishing.service.gov.uk/government/uploads/system/uploads/attachment_data/file/892376/COVID_stakeholder_engagement_synthesis_beyond_the_data.pdf. (Access date: 17.12.2020).

For peer review only

20. West-Oram P. Solidarity is for other people: identifying derelictions of solidarity in responses to COVID-19. Journal of Medical Ethics. 2020. 10.1136/medethics-2020-106522.
21. Prainsack B, Buyx A. Solidarity: reflections on an emerging concept in bioethics. Nuffield Council on Bioethics; 2011. https://www.nuffieldbioethics.org/assets/pdfs/Solidarity-report.pdf. (Access date: 03.12.2020).
22. Coggon J, Regmi S. Covid-19: Government guidance on emergency rationing of critical care is needed to support professional decision making [Internet]. BMJ Opinion2020. [24.10.2020]. Available from: https://blogs.bmj.com/bmj/2020/04/24/covid-19-government-guidance-on-emergency-rationing-of-critical-care-is-needed-to-support-professional-decision-making/. (Access date: 24.10.2020).
23. Khangura S, Konnyu K, Cushman R, *et al.* Evidence summaries: the evolution of a rapid review approach. Systematic Reviews. 2012;1(1):10. 10.1186/2046-4053-1-10.
24. Thomas J, Harden A. Methods for the thematic synthesis of qualitative research in systematic reviews. BMC Medical Research Methodology. 2008;8:1-10. 10.1186/1471-2288-8-45.
25. Tong A, Flemming K, McInnes E, *et al.* Enhancing transparency in reporting the synthesis of qualitative research: ENTREQ. BMC Medical Research Methodology. 2012;12(1):181-. 10.1186/1471-2288-12-181.
26. EPPI-Centre. Covid-19: a living systematic map of the evidence UCL: Institute of Education; 2020 [updated 08.01.2021. Available from: http://eppi.ioe.ac.uk/cms/Projects/DepartmentofHealthandSocialCare/Publishedreviews/COVID-19Livingsystematicmapoftheevidence/tabid/3765/Default.aspx. (Access date: 07.10.2020).
27. The Health Foundation. COVID-19 policy tracker: The Health Foundation; 2020 [Available from: https://www.health.org.uk/news-and-comment/charts-and-infographics/covid-19-policy-tracker. (Access date: 08.09.2020).
28. The Centre for Informed Consent Integrity. Covid-19: Governance, ethics, evidence, policy practice: Global Foundation; 2020 [Available from: https://ge2p2global-centerforinformedconsentintegrity.org/category/covid-19/. (Access date: 12.10.2020).
29. NICE. Speciality guides: NICE; 2020 [Available from: https://www.nice.org.uk/covid-19/specialty-guides. (Access date: 02.10.2020).
30. UK Government. Freedom of Information Act. Sect. ss 19 and 20 (2000).
31. Information Commissioner's Office. Model publication scheme: Freedom of Information Act. 2015. https://ico.org.uk/media/for-organisations/documents/1153/model-publication-scheme.pdf. (Access date: 12.11.2020).
32. Ouzzani M, Hammady H, Fedorowicz Z, *et al.* Rayyan—a web and mobile app for systematic reviews. Systematic Reviews. 2016;5(1):210. 10.1186/s13643-016-0384-4.
33. Stevens S, Pritchard A. Third Phase of NHS Response to Covid-19. 2020. https://www.england.nhs.uk/coronavirus/wp-content/uploads/sites/52/2020/07/20200731-Phase-3-letter-final-1.pdf. (Access date: 15.09.2020).
34. National Voices. Five principles for the next phase of the Covid-19 response. 2020. https://www.nationalvoices.org.uk/sites/default/files/public/publications/5_principles_statement_091020.pdf. (Access date: 17.08.2020).
35. Brouwers M, Kho ME, Browman GP, *et al.* AGREE II: Advancing guideline development, reporting and evaluation in healthcare. Canadian Medical Association Journal. 2010;182(18):E839-E42. 10.1503/cmaj.090449.
36. Tricco AC, Garritty CM, Boulos L, *et al.* Rapid review methods more challenging during COVID-19: commentary with a focus on 8 knowledge synthesis steps. J Clin Epidemiol. 2020;126:177-83. 10.1016/j.jclinepi.2020.06.029.
37. The AGREE Next Steps Consortium. Appraisal of Guidelines for Research & Evaluation II: AGREE II Instrument. 2017. https://www.agreetrust.org/wp-content/uploads/2017/12/AGREE-II-Users-Manual-and-23-item-Instrument-2009-Update-2017.pdf. (Access date: 04.08.2020).

For peer review only

38. Academy of Medical Royal Colleges. Principles for reintroducing health services. COVID-19. 2020. https://www.aomrc.org.uk/wp-content/uploads/2020/05/COVID-19_Principles_for_reintroducing_healthcare_services_0520.pdf (Access date: 24.09.2020).

39. Academy of Medical Royal Colleges. Reset, restore and recovery: staff support. 2020. https://www.aomrc.org.uk/wp-content/uploads/2020/06/200622_RRR_-Staff_support.pdf (Access date: 24.09.2020).

40. Academy of Medical Royal Colleges. COVID-19. Effects on health from non-COVID-19 conditions and moving forward to deliver healthcare for all. 2020. https://www.aomrc.org.uk/wp-content/uploads/2020/05/200515_COVID-19_moving_forward_to_deliver_healthcare.pdf. (Access date: 24.09.2020).

41. Academy of Medical Royal Colleges. Preparing for COVID-19 surges and winter. 2020. https://www.aomrc.org.uk/wp-content/uploads/2020/07/200717_Preparing_for_covid-19_surges_winter.pdf. (Access date: 24.09.2020).

42. Academy of Medical Royal Colleges. Health protection: public and professional responsibilities. 2020. https://www.aomrc.org.uk/wp-content/uploads/2020/06/200611_Health_protection_public_professional_responsibilities.pdf (Access date:

43. Academy of Medical Royal Colleges. Reset, restore and recovery: medical education and training. 2020. https://www.aomrc.org.uk/wp-content/uploads/2020/06/200622_RRR_Medical_Education_Training.pdf. (Access date: 24.09.2020).

44. Academy of Medical Royal Colleges. Reset, restore and recovery: equalities. 2020. https://www.aomrc.org.uk/wp-content/uploads/2020/06/200622_RRR_Equalities.pdf. (Access date: 24.09.2020).

45. Palmer C, Johnson P. Second phase of NHS response to COVID-19 for cancer services. 2020. London, UK. https://www.england.nhs.uk/coronavirus/wp-content/uploads/sites/52/2020/06/C0511-second-phase-of-nhs-response-to-covid-19-for-cancer-services-letter.pdf. (Access date:

46. National Health Service. Operating framework for urgent and planned services in hospital settings during COVID-19. 2020. https://covidlawlab.org/wp-content/uploads/2020/06/Operating-framework-for-urgent-and-planned-services-within-hospitals.pdf. (Access date: 29.09.2020).

47. National Health Service. WRES briefing for board and COVID-19 emergency preparedness, resilience and response (EPRR) membership in the NHS. 2020. https://www.england.nhs.uk/coronavirus/publication/wres-briefing-for-board-and-covid-19-emergency-preparedness-resilience-and-response-eprr-membership-in-the-nhs/. (Access date: 29.09.2020).

48. Public Health England. COVID-19: Guidance for the remobilisation of services within health and care settings, infection prevention and control recommendations 2020. https://assets.publishing.service.gov.uk/government/uploads/system/uploads/attachment_data/file/910885/COVID-19_Infection_prevention_and_control_guidance_FINAL_PDF_20082020.pdf. (Access date:

49. National Institute for Health and Care Excellence. Delivering paediatric elective surgery service during the COVID-19 pandemic. 2020. [18.09.2020]. Available from: https://www.nice.org.uk/sharedlearning/delivering-a-paediatric-elective-surgery-service-during-the-covid-19-pandemic. (Access date: 18.09.2020).

50. Jay N. COVID-19 - guidance for planning paediatric staffing and rotas. 2020. https://www.rcpch.ac.uk/resources/covid-19-guidance-planning-paediatric-staffing-rotas. (Access date: 29.09.2020).

51. Children and Young People Engagement Team. COVID-19 & Us - views from RCPCH & Us. 2020. https://www.rcpch.ac.uk/resources/covid-19-us-views-rcpch-us. (Access date: 25.09.2020).

For peer review only

52. Wilkinson D, Linney, M. Ethics framework for use in acute paediatric settings during COVID-19 pandemic. 2020. https://www.rcpch.ac.uk/resources/ethics-framework-use-acute-paediatric-settings-during-covid-19-pandemic. (Access date:
53. Royal College of Paediatrics and Child Health. National guidance for the recovery of elective surgery in children. RCPCH; 2020. https://www.rcpch.ac.uk/resources/national-guidance-recovery-elective-surgery-children#summary-of-recommendations. (Access date: 25.09.2020).
54. Royal College of Paediatrics and Child Health. Reset, Restore, Recover - RCPCH principles for recovery. 2020. https://www.rcpch.ac.uk/resources/reset-restore-recover-rcpch-principles-recovery. (Access date: 25.09.2020).
55. Fabbrani E, ., Mahajan R, . It is right to restart services, but we must do so in a safe way. Royal College of Anaesthetists2020. [30.09.2020]. Available from: https://www.rcoa.ac.uk/blog/it-right-restart-services-we-must-do-so-safe-way. (Access date: 30.09.2020).
56. The Royal College of Midwives. Antenatal Care for women without suspected or confirmed COVID-19 and living in a symptom free household. 2020. https://www.rcm.org.uk/media/4180/clinical-guidance-briefing-one-antenatal-care-for-women-without-symptomsfinalv4-1.pdf. (Access date: 29.09.2020).
57. The Royal College of Midwives. RCM Briefing on Re-introduction of visitors to Maternity Units across the UK during the COVID-19 pandemic. 2020. https://www.rcm.org.uk/media/4161/rcm-briefing-on-reintroduction-of-visitors-to-maternity-units-in-the-covid-pandemic-003.pdf. (Access date: 29.09.2020).
58. The Royal College of Midwives. RCM Clinical Briefing Sheet: 'freebirth' or 'unassisted childbirth' during the COVID-19 pandemic. 2020. https://www.rcm.org.uk/media/3923/freebirth_draft_30-april-v2.pdf. (Access date: 30.09.2020).
59. Brigante L, Jokinen, M., Ross-Davie, M., Harlev-Lam, B., Morris, E., O'Brien, P., Jardine, J,. Relph, S., Powell, A. Guidance for provision of midwife-led settings and home birth in the evolving coronavirus (COVID-19) pandemic. 2020. https://www.rcm.org.uk/media/4099/2020-05-22-guidance-for-provision-of-midwife-led-settings-and-home-birth-in-the-evolving-coronavirus-covid-19-pandemic.pdf (Access date:
60. The Royal College of Midwives. Equality essentials: Appropriate risk assessment during the current pandemic. 2020. https://www.rcm.org.uk/media/3939/risk-assessment-wraparound-guidance-a3-may-2020.pdf (Access date: 30.09.2020).
61. The Royal College of Midwives. COVID-19 impact on Black, Asian and Minority ethnic (BAME) women. 2020. https://www.rcm.org.uk/media/4164/covid-19-impact-on-black-asian-and-minority-ethnic-bame-women.pdf. (Access date: 29.09.2020).
62. Royal College of Obstretricians and Gynaecologists. Principles for the testing and triage of women seeking maternity care in hospital settings during the COVID-19 pandemic: a supplimentary framework for maternity healthcare professionals. London, UK; 2020. https://www.rcog.org.uk/globalassets/documents/guidelines/2020-08-10-principles-for-the-testing-and-triage-of-women-seeking-maternity-care-in-hospital-settings-during-the-covid-19-pandemic.pdf. (Access date: 29.09.2020).
63. Ross-Davie M, Lambert, J., Brigante, L., Livingstone, C., Crowe, S., Pandya, P., Morris, E., O'Brien, P., Jardine, J., Relph, S., Goodyear, G. Guidance for antenatal and postnatal services in the evolving coronavirus (COVID-19) pandemic. 2020. https://www.rcm.org.uk/media/4132/2020-06-18-guidance-for-antenatal-and-postnatal-services-in-the-evolving-coronavirus-covid-19-pandemic.pdf. (Access date: 30.09.2020).
64. The Royal College of Midwives. Antenatal care for women with current suspected or confirmed COVID-19 or with a member of their household with suspected or confirmed COVID-19. 2020. https://www.rcm.org.uk/media/4172/professional-briefingpaper-two-antenatal-care-for-women-with-suspected-or-confirmed-covid19-v31-240720.pdf. (Access date: 29.09.2020).

For peer review only

65. The Royal College of Midwives. Domestic Abuse [press release]. 2020. https://www.rcm.org.uk/media/4067/identifying-caring-for-and-supporting-women-at-risk-of_victims-of-domestic-abuse-during-covid-19-v1__13052020final.pdf. (Access date: 29.09.2020).
66. The Royal College of Midwives. Bereavement Care in Maternity Services During COVID-19 pandemic. 2020. https://www.rcm.org.uk/media/4162/maternity-bereavement-care-during-covid-19-v5-150720.pdf. (Access date: 30.09.2020).
67. The Royal college of Midwives. Postnatal Care for women with suspected or confirmed COVID-19 2020. https://www.rcm.org.uk/media/4207/briefing-no-9-postnatal_clinical_advice_with-covid_140820.pdf. (Access date: 30.09.2020).
68. The Royal College of Midwives. Virtual Consultations. 2020. https://www.rcm.org.uk/media/4192/virtual-consultations-v20-24-july-2020-review-24-august-2020-1.pdf. (Access date: 30.09.2020).
69. Royal College of Anaethetists. Restarting planned surgery in the context of the COVID-19 pandemic. 2020. https://static1.squarespace.com/static/5e6613a1dc75b87df82b78e1/t/5eac2a173d65cd27933fca88/1588341272367/Restarting-Planned-Surgery.pdf. (Access date: 28.09.2020).
70. National Health Service. Delivering midwifery intrapartum care where local COVID-19 escalation protocols are required to be enacted. 2020. Report No.: 001559. https://www.nice.org.uk/Media/Default/About/COVID-19/Specialty-guides/Delivering-midwifery-intrapartum-care-where-local-COVID-19-escalation-protocols-are-required-to-be-enact.pdf. (Access date: 30.09.2020).
71. Kasaven LS, Saso S, Barcroft J*, et al.* Implications for the future of Obstetrics and Gynaecology following the COVID-19 pandemic: a commentary. BJOG : an international journal of obstetrics and gynaecology. 2020;127(11):1318-23. 10.1111/1471-0528.16431.
72. Renfrew MJ, Cheyne H, Craig J*, et al.* Sustaining quality midwifery care in a pandemic and beyond. Midwifery. 2020;88:102759. 10.1016/j.midw.2020.102759.
73. Sokol D, Dattani R. How should surgeons obtain consent during the covid-19 pandemic? BMJ. 2020;369:m2539. 10.1136/bmj.m2539.
74. National Institute for Health and Care Excellence. COVID-19 rapid guideline: arranging planned care in hospitals and diagnostic services 2020. Report No.: NG179. https://www.nice.org.uk/guidance/ng179/resources/covid19-rapid-guideline-arranging-planned-care-in-hospitals-and-diagnostic-services-pdf-66141969613765. (Access date: 18.09.2020).
75. Huxtable R. Bin it or pin it? Which professional ethical guidance on managing COVID-19 should I follow? BMC Medical Ethics. 2020;21(1):60. 10.1186/s12910-020-00491-5.
76. NHS Confederation. A road map to reset our shared future. 2020. https://www.nhsconfed.org/-/media/Confederation/Files/Publications/Documents/A-road-map-to-reset-our-shared-future.pdf. (Access date: 25.11.2020).
77. Viens AM, McGowan CR, Vass VM. Moral distress among healthcare workers: ethics support is a critical part of the puzzle. BMJ Blogs2020. [cited 22nd September 2020 16.11.2020]. Available from: https://blogs.bmj.com/bmj/2020/06/23/moral-distress-among-healthcare-workers-ethics-support-is-a-crucial-part-of-the-puzzle/. (Access date: 16.11.2020).
78. Fritz Z, Huxtable R, Ives J*, et al.* Ethical road map through the covid-19 pandemic. BMJ. 2020;369:m2033. 10.1136/bmj.m2033.
79. UK Government. Our plan to rebuild: The UK Government's COVID-19 recovery strategy. 2020. London, UK. https://www.gov.uk/government/publications/our-plan-to-rebuild-the-uk-governments-covid-19-recovery-strategy/our-plan-to-rebuild-the-uk-governments-covid-19-recovery-strategy#our-approach-a-phased-recovery. (Access date: 03.11.2020).
80. Department of Health and Social Care. Meeting note. 2020. London. UK Government. https://app.box.com/s/jnwfj507tzuho5fjx181hwgmeh868vgh/file/677938102935. (Access date: 12.01.2021).

For peer review only

81. Huxtable R. COVID-19: where is the national ethical guidance? BMC Medical Ethics. 2020;21(1):32. 10.1186/s12910-020-00478-2.
82. Scottish Government. COVID-19 guidance: ethical advice and support framework. 2020. 1-21. https://www.gov.scot/publications/coronavirus-covid-19-ethical-advice-and-support-framework/. (Access date: 12.01.2021).
83. Together for Devon. Devon Ethics Paper for Organisational Boards. Devon; 2020. https://www.togetherfordevon.uk/download/devon-ethics-paper-for-organisational-boards/. (Access date: 12.01.2021).
84. NHS England & NHS Improvement. Good practice for working with people and communities during the COVID-19 outbreak. 2020. https://www.england.nhs.uk/participation/news/. (Access date: 25.11.2020).
85. Bok S. Lying: moral choice in public and private life. New York: Pantheon.; 1978.
86. Kerasidou A. Trust me, I'm a researcher!: The role of trust in biomedical research. Medicine, Health Care & Philosophy. 2017;20(1):43-50. 10.1007/s11019-016-9721-6.
87. O'Neill O. Autonomy and trust in bioethics. Cambridge: Cambridge University Press [electronic book]; 2002. Available from: http://ebookcentral.proquest.com/lib/liverpool/detail.action?docID=202188.
88. UK Government. The NHS Constitution: the NHS belongs to us all. 2015. London, UK. https://assets.publishing.service.gov.uk/government/uploads/system/uploads/attachment_data/file/480482/NHS_Constitution_WEB.pdf. (Access date: 16.11.2020).

For peer review only

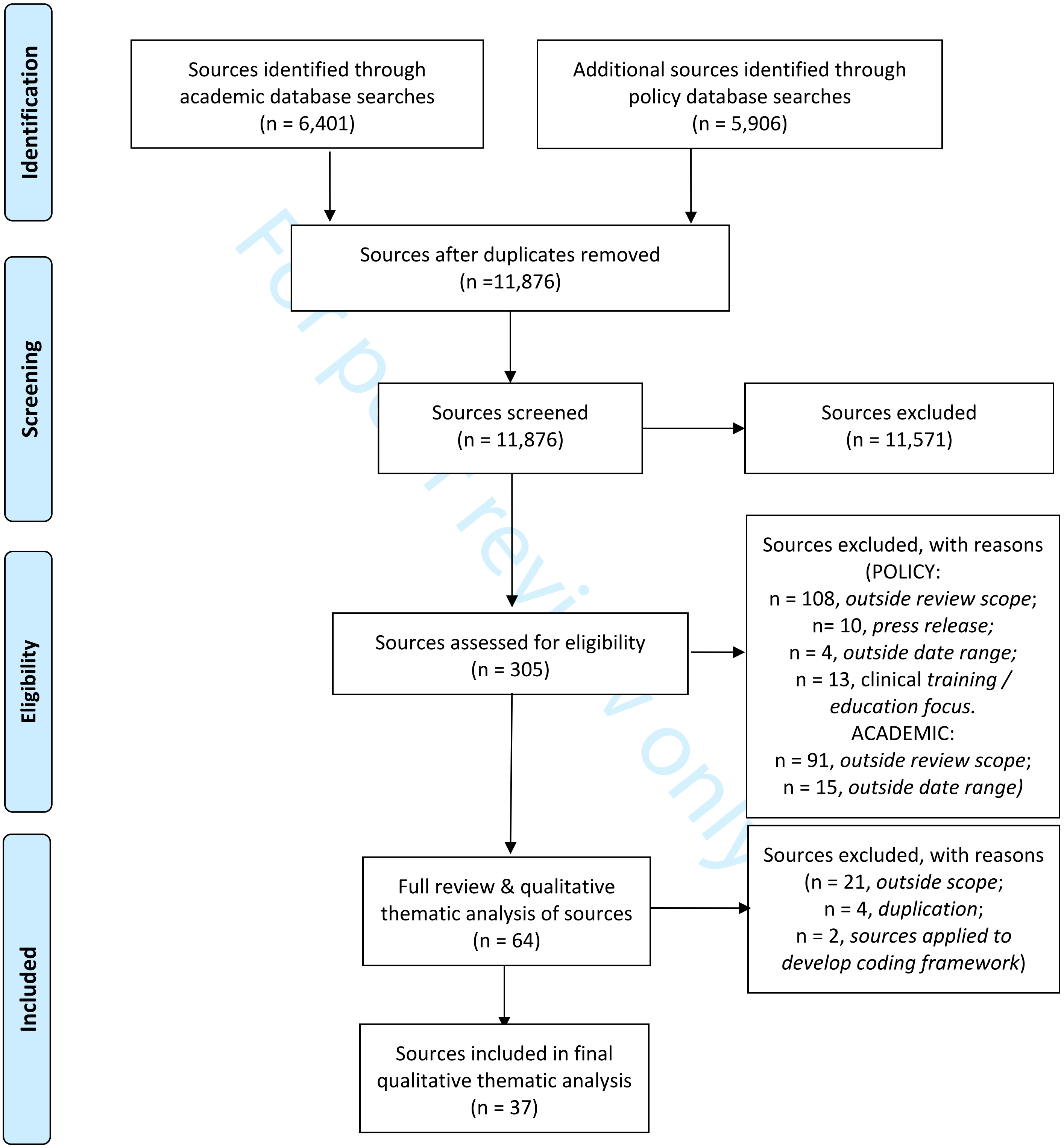

From: Moher D, Liberati A, Tetzlaff J, Altman DG, The PRISMA Group (2009). *P*referred *R*eporting *I*tems for *S*ystematic Reviews and *M*eta-*A*nalyses: The PRISMA Statement. PLoS Med 6(7): e1000097. doi:10.1371/journal.pmed1000097

For more information, visit www.prisma-statement.org.

For peer review only - http://bmjopen.bmj.com/site/about/guidelines.xhtml

Which ethical values underpin England's National Health Service reset of paediatric and maternity services following Covid-19: a rapid review.

SUPPLIMENTARY FILES

FILE 1: RAPID REVIEW PROTOCOL

Background and review rationale:
The response to Covid 19 (C19) will have far-reaching consequences for the NHS. The *Everyday and pandemic ethics* project will explore how the ethical issues created by this response have been approached by providers of non-C19 services. Notably we will explore how decisions on service prioritisation and reconfiguration have been made in the "reset" phase that has followed the first acute phase of the C19 pandemic. We define this "reset" phase as commencing from April 29th 2020, as NHS services were instructed on that date to prepare to recommence the delivery of non-covid surgical services (https://www.england.nhs.uk/coronavirus/wp-content/uploads/sites/52/2020/04/second-phase-of-nhs-response-to-covid-19-letter-to-chief-execs-29-april-2020.pdf). The "resetting" of NHS services encompasses the following:

- The resumption of service delivery incorporating revised procedures and practices to control the spread of C19 (e.g. the wearing of face coverings);
- Preparation for, and management of, second "waves" or recurrent spikes of C19, at both the national and local levels;
- The opportunities to reconfigure health services, for example accelerating the use of tele-medicine.

The focus on the reset phase emphasises the unique factors affecting ethical decision-making as services are re-established following the acute phase of the C19 pandemic.

We will focus on ethical decision-making in two non-C19 areas: maternity and paediatrics. We have chosen these areas because they have been significantly affected by the C19 response due to resource allocation away from these areas, with professional and patient organisations highlighting problematic effects on both areas (Association of Paediatric Anaestetists of Great Britain and Ireland, 2020; First 1001 Days Movement, 2020; McDonald et al., 2020). Specifically, the review will focus on "maternity services" (pre-natal, intrapartum, and post-partum care); and the resumption of paediatric surgery (encompassing critical / intensive care admissions, surgery, hospital discharge, and aftercare, referred to as "paediatric critical care and surgery services") during the C19 reset phase.

The objective of this review is to provide an initial understanding of the ethical values explicitly or implicitly engaged to inform decision-making about maternity services, and the resumption of paediatric critical care and surgery during the reset phases following the C19 pandemic in England. We adopt a pragmatic approach in order to make the best available use of existing evidence relating to this topic. The evidence will include diverse sources such as Government and Hospital trust policies, statements and decision support tools; reports and statements from professional bodies and charitable organisations; and evidence reviews and commentaries in academic journals. The approach aims to be broad and inclusive by combining searches of bibliographic databases with grey literature, hand searching, snowballing references of included sources, and engaging key topic stakeholders in an effort to verify completeness of sources. These approaches aim to ensure flexibility in identifying relevant sources both systematically and in the most efficient and pragmatic manner.

We will report key characteristics of all sources, and will appraise sources against a coding framework adapted from the Ethical Framework embedded in the Government's Pandemic Flu policy (UK Government, 2017). This framework is intended to guide all UK NHS decision-making during the rapid

For peer review only - http://bmjopen.bmj.com/site/about/guidelines.xhtml

readjustment of services due to a pandemic. Recognising that the reset phase requires different decision-making to the acute phase, we have adapted the framework by drawing upon two interlinked national documents (a letter on “Third phase of NHS response to Covid”, 31st July 2020 (Stevens & Pritchard, 2020); and the National Voices “Five principles for the next phase of the Covid-19 response”, published June 2020 (National Voices, 2020)). These adaptations aim to reflect the particular ethical considerations relevant to the “reset” phase. We recognise that this adaptation creates a tension between the rapid review methodology and findings, which we discuss alongside the revised framework below. In our analysis we will draw upon the systematic review of reasons approach (Strech & Sofaer, 2012) to facilitate explicit consideration of ethical values being applied to inform decision-making in non-C19 maternity services, and paediatric critical care and surgery services during the C19 reset phases in England.

This rapid evidence review forms the first stage of a larger project, providing a snapshot of ethical decision-making in maternity and paediatric care to inform subsequent stages of the *Everyday and Pandemic Ethics* study. Review findings will be available as immediate recommendations for ethical best practice – for example by examining the transparency of written policies against standards in the 2016 Pandemic Flu Policy - for paediatric and maternity services delivery during the C19 reset phases.

Objective

The objective of this review is to answer the question: what ethical values guide decision-making in non-C19 paediatric critical care and surgery and maternity services during the C19 reset phases in England? Achieving this objective will entail exploring a range of decision-making factors, such how are involved in decision-making, what decisions have been made, and how decisions are justified, identifying implicit and explicit ethical values.

Methodology

To ensure a rigorous review methodology, we have drawn upon the ENTREQ guidelines for qualitative research synthesis (Tong, Flemming, McInnes, Oliver, & Craig, 2012) and the systematic review of reasons approach developed for normative review questions (Strech & Sofaer, 2012). Integrating these approaches address the critique that literature reviews exploring normative considerations often fail to clearly report the methodological approach taken (Mertz, Strech, & Kahrass, 2017).

Inclusion and exclusion criteria

Inclusion criteria

This review will consider sources developed to guide non-C19 paediatric critical care and surgery services and maternity services during the reset phases of C19; or that discuss the application of ethical values to paediatric critical care and surgery services and maternity services during the reset phases of C19.

The review will include sources relating to England, including national policies (that include England), and policies from Trusts and individual hospitals across England, including our case study sites (in North West England and the Midlands). We will be restricted to sources written in the English language, and published after 29th April 2020.

Exclusion criteria

Sources published prior 29th April 2020, that discuss healthcare delivery broadly; or that discuss maternity or paediatric critical care or surgery services during the acute phase of the C19 pandemic in England (defined as the start of lockdown on 23rd March until the 29th April 2020) will be excluded.

Data sources

The review will include the following data sources:

For peer review only

- National policies guiding the implementation of non-C19 maternity services, and/or paediatric critical care and surgery services; and/or providing an ethical framework or decision-making tools for healthcare reorganisation of these services during the C19 reset phases;
- Local trust and hospital policies guiding the implementation of non-C19 maternity and paediatric critical care and surgery services; and/or providing an ethical framework or decision-making tools for healthcare reorganisation in these services during the C19 reset phases;
- Guidelines and statements from Royal Medical Colleges relating to the implementation of non-C19 maternity and paediatric critical care and surgery services and/or providing an ethical framework or decision-making tools for healthcare reorganisation in these services during the C19 reset phases;
- Working papers and committee reports discussing the re-orientation of non-C19 maternity and paediatric critical care and surgery services during the C19 reset phases;
- Evidence reviews and primary qualitative and quantitative research on the re-orientation of non-C19 maternity and paediatric critical care and surgery services during the C19 reset phases;
- Peer-reviewed commentaries and grey-literature discussing experiences of non-C19 maternity, and paediatric critical care and surgery services during the C19 reset phases.

All sources will be obtained from online platforms, or via e-mail for Freedom of Information requests and stakeholder contributions.

Electronic search strategy
We will conduct searches in September 2020, with an additional search prior to the publication of the review to check for sources published in the interim. We will search the following academic bibliographic databases: PubMed and PubMeds Covid-19 database LitCOVID (https://www.ncbi.nlm.nih.gov/research/coronavirus/). We will also search clearing houses of C19 related research including the EPPI Centre living map of Covid-19 evidence (http://eppi.ioe.ac.uk/cms/Projects/DepartmentofHealthandSocialCare/Publishedreviews/COVID-19Livingsystematicmapoftheevidence/tabid/3765/Default.aspx), COVID END (https://www.mcmasterforum.org/networks/covid-end), evidence aid (https://evidenceaid.org/evidence/coronavirus-covid-19/ - which includes reviews being conducted by the Campbell Collaboration), and the Cochrane Collaboration.

For academic bibliographic databases we will search using the following terms:
1. (Covid OR Covid-19 OR coronavirus* OR SARS-CoV-2 OR Severe Acute Respiratory Syndrome OR pandemic) AND
2. (Matern*) OR (pre-natal OR inter-partum OR post-natal OR perinatal) OR (labour OR pregnan*) OR (obstetrics) OR (birth*) OR (Midwife*) AND
3. (paediatric OR pediatric) AND (critical OR intensive OR acute) OR (operati* OR theatre*) OR (child*) OR (surg*) AND
4. (doctor) OR (nurs*) AND
5. (service*) OR (design OR deliver*) OR (allocat* OR priorit*) OR (care) OR (policy OR guideline*)

Searchers will be conducted step-wise, first conducting searches relating to Maternity service combining rows 1,2, 4 and 5 above; and secondly for Paediatric critical care and surgery, combining rows 1,3, 4 and 5 above.

To complement academic databases, and recognising the scope of the research question, we will also search grey literature sources including the websites of NHS Trusts (including our case study sites),

the UK Government (gov.uk), and websites of professional bodies (e.g. Academy of Royal Colleagues and the Royal College of Paediatrics / Midwifery and NICE). We will also search clearinghouses of C19 related grey literature such as policy documents, for example the Health Foundation C19 Policy Tracker (https://www.health.org.uk/news-and-comment/charts-and-infographics/covid-19-policy-tracker).

Study screening methods

We will review all identified sources and any duplicates removed. Two members of the research team (AC, PB, CR, SF and LF) will double screen all identified results. Screening will be based on title and abstract / summary (where available). Where these are not available or no definitive decision can be made about whether a source meets the review inclusion criteria based on title and abstract/summary screening, additional full text review will be undertaken. To operationalise the inclusion criteria we applied the following scoring system:

0. Not included
1. Included: Identifies the approach taken to decision making (e.g. discusses a decision-making tool or framework)
2. Included: Identifies what decision has been made
3. Included: Identifies a justification for the decision taken

Where a source meets more than one of the inclusion criteria, all will be identified. Disagreements in double screening will be resolved through discussion with a third member of the review team (HD) not involved in initial screening to reach a consensus decision about inclusion or exclusion.

We will document all searches and screening assessments in a flow chart, with an accompanying narrative explanation, including explicit reasons for study exclusion.

Using the Freedom of Information Act 2000

The Freedom of Information Act 2000 (FOI) imposes two main duties on public authorities: one to proactively provide information, and the other to respond to requests for information. A model 'publication scheme' has been produced which public authorities are obliged to follow in making relevant information available. The model publication scheme sets out various classes of information, which are tailored to different authorities by a 'definition document' for each type of organisation. The classes of information are as follows:

- Who we are and what we do
- What we spend and how we spend it
- What our priorities are and how we are doing
- How we make decisions
- Our policies and procedures
- Lists and registers
- The services we offer

To aid access to NHS Trust information we will review Trusts' Freedom of Information Act Publication schemes and submit freedom of information (FOI) requests. Our publication scheme reviews and FOI requests will target our case study sites, as well as additional NHS Trusts with Clinical Ethics Committees as listed on the UKs Clinical Ethics Network. Both the reviews and the FOI requests will explicitly focus on sources (e.g. meeting minutes, policies, or decision-making tools) guiding maternity services and paediatric critical care and surgery services developed for the reset period. FOI requests will be submitted to individual hospitals and NHS Trusts, as well as at regional and national decision-making levels. To mirror database searches, we will repeat the publication scheme reviews and the FOI requests prior to publication of the review for the inclusion of additional sources.

For peer review only

After the initial searches, publication scheme reviews and results from FOI requests, we will share results with Trust and project stakeholders to conduct a completeness check and request additional missing sources be identified for screening and potential inclusion. We will furthermore search citations of included sources for snowball sampling.

Appraisal of sources

Given the reviews focus on normative values, we will apply the PROGRESS Plus tool[1] to identify the extent to which sources consider characteristics recognised to affect health equity (https://methods.cochrane.org/equity/projects/evidence-equity/progress-plus). This tool covers factors including place of residence, race/ethnicity/culture/language, occupation, gender/sex, religion, education, socioeconomic status, and social capital (O'Neill et al., 2014); as well as "plus" factors such as age and disability, relational features (such as single parent household), and time-dependent relationships (e.g. receiving in-patient care). Assessing sources against these will identify the extent to which sources are systematically considering various aspects of health equity.

In addition, for peer reviewed literature we will apply the relevant CASP checklist[2] (https://casp-uk.net/casp-tools-checklists/), and for policy sources the AGREE-II tool developed for assessing healthcare practice guidelines (Brouwers et al., 2010).

Data extraction and management

We will report the following characteristics of included sources:

- Publication type (e.g. policy, report, professional body guideline, peer reviewed article, commentary piece, decision-support tool, etc);
- Month and year of publication;
- Population (maternity or paediatric services);
- Source scope (national, regional, trust, hospital, etc);
- Where relevant for primary research we will also report: the primary research question, methodology, number of participants, and analysis approach.

Sources will be analysed against a coding framework. This coding framework has been developed by modifying the Ethical Framework embedded in the Government's Pandemic Flu policy (UK Government, 2017). The Ethical Framework in the Pandemic Flu Policy is guided by the fundamental principle of equal concern and respect, accompanied by 8 embedded principles designed to be applied as a checklist to help ensure that the full-range of ethical issues are considered in decision-making processes. It is the only framework explicitly intended to guide all UK NHS decision-making during the rapid readjustment of services due to a pandemic. However, recognising that the reset phase requires a different decision-making to the acute phase, we adapted the framework by drawing upon two interlinked national documents: (1) a letter from the NHS Chief Executive and Chief Operating Officer on "Third phase of NHS response to Covid", dated 31st July 2020 (Stevens & Pritchard, 2020), and (2) the National Voices "Five principles for the next phase of the Covid-19 response" published in June 2020 (National Voices, 2020). Our coding framework retains the Pandemic Flu 8 embedded principles, but adjusts their specification according to how they are operationalised in these two documents. We recognise this adaptation creates a methodological tension in our review as our coding framework is based upon a Framework adapted according to ethical documents relevant to the review scope and purpose. We believe this approach is justifiable given the lack of an overarching framework tailored

[1] This aspect of the review was not conducted due to time constraints.

[2] No peer reviewed studies reporting original data were included in the review, therefore this tool was not applied.

to the reset phase, and the need for a coding framework for the review that reflects the ethical specificities of this phase.

Extracting information from sources in relation to each of these adapted principles will identify whether the source engages with the normative values identified as important when making decisions during the C19 reset phase. The principles (retained from the national pandemic flu policy) and adapted sub-domains are as follows:

Ethical principle (from Pandemic Flu Ethical Framework)	Adapted sub-domain (based on NHS letter and National Voices Five Principles)
**Respect**	Involvement (i.e. right to express views on matters affecting them, engaging those affected by decisions)
	Respecting choices about personalised care (best interests of person as a whole)
	Collaborative working / engagement (organisational coordination; NHS volunteer scheme, clinical teams, CCGs, local authorities; co-production with voluntary sector, patient orgs etc)
**Recognising harms & balancing against benefits (physical, psychological, social & economic) - proportionality**	Recover operation of healthcare (inc. addressing backlog of care needs, resuming home visits for vulnerable / shielding where appropriate)
	Safety of NHS staff (physical, psychological, systemic inequalities, flexible working)
	Embrace new ways of working (e.g. telemedicine, home visits etc)
	Enhance crisis responsiveness (second wave)
	Accelerate preventative programmes (obesity reduction, seasonal flu, outreach to marginalised groups)
	Responsiveness (adapt plans to new circumstances / information)
**Reciprocity**	Concept of mutual exchange: take responsibility for own behaviour, reduce others expose others to risks
	Protect those at risk of C19 (physically, socially, BAME etc)
**Fairness**	Inclusivity in service recovery (e.g. barriers or access needs, support those with unequal access to care)
	Patient prioritisation (to address backlog i.e. clinical urgent / longest waiting etc)
	Reduce health inequalities (social inequalities & social determinants of health)
	Everyone matters equally & weighted equally in policies & any disproportionate impact on one particular group is accounted for
**Accountability**	Transparency (i.e. document decisions, clarity of who is responsible for decisions, governance arrangements, assess against milestones, sharing information to help others)

Recognising that the reset phase may incorporate responding to second waves of C19 infections, for example through localised lockdowns (as provided for in the UK Governments Covid-19 Contain framework: https://www.gov.uk/government/publications/containing-and-managing-local-coronavirus-covid-19-outbreaks/covid-19-contain-framework-a-guide-for-local-decision-makers), the principles and sub-domains within this assessment framework may be inductively revised on the basis of the sources reviewed. We will report any development of the framework as an outcome of the rapid review.

For peer review only

We will apply a scoring system to assess the inclusion and application of each principle domain. This will entail a 2-stage process, first answering "yes/no" to its inclusion and, secondly, rating application of each domain on a scale of 1-3, where:

1. ethical principle(s) inferred or mentioned but not clearly applied;
2. ethical principle(s) identified and its application described; and
3. ethical principle(s) application is discussed in-depth, including balancing against other principles.

Data synthesis
To further explore the data, we will conduct further analysis of sources from our case study sites (North West England and the Midlands) to conduct a thematic synthesis (Thomas & Harden, 2008)[3]. This approach will draw upon the review of reasons where the data is explored to identify reasons for adopting particular normative positions, and the consistency of these reasons across sources and settings (maternity or paediatrics). This will help to surface the range of reasons informing decision-making processes, and experiences of these decisions by those affected.

Data synthesis will be led by AC and PB, with regular review and discussion with the wider research team to ensure rigor of the approach to analysis.

Reporting
We will report this rapid review as brief reports summarising the approach to paediatric critical care and surgery services, and maternity services, during the reset phase of the C19 pandemic. This will identify the ethical values informing paediatric critical care and surgery services, and maternity services, during the reset phase of the C19 pandemic, and highlighting case study examples that explore the reasons for adopting a particular normative position. The report will be disseminated in the form of a short brief, shared with our stakeholder group comprised of representatives of National bodies, case study Trusts and Hospitals, and other relevant parties. We will also disseminate the review findings via social media (e.g. Twitter) and our project website (https://www.liverpool.ac.uk/population-health-sciences/departments/health-services-research/key-projects/resetethics/).

We will also develop a rapid review publication reporting the full results. It will go into more depth than the brief report about the methodology, and will offer an in-depth description of the response to planning for the reset phase of maternity services and paediatric critical care and surgery services in England. We will explore examples of good practice – such as where specific sources have engaged with the full breadth of ethical considerations, or where there is transparency in descriptions of ethical engagement and decision-making processes. From this, we will make recommendations for addressing areas where the normative basis of adopting specific approaches to service planning and delivery are unclear.

REFERENCES
Association of Paediatric Anaestetists of Great Britain and Ireland, A. (2020). APAGBI position statement regarding the delivery and recovery of Children's surgery during the coronavirus pandemic [Press release]. Retrieved from https://www.apagbi.org.uk/sites/default/files/inline-files/APA%20statement%2015.05.20%20Final.pdf
Brouwers, M., Kho, M. E., Browman, G. P., Burgers, J. S., Cluzeau, F., Feder, G., . . . Consortium, T. A. N. S. (2010). AGREE II: Advancing guideline development, reporting and evaluation in

[3] This aspect of the review is ongoing and is based primarily upon the Publication Scheme review data. In our paper we report initial findings from this.

healthcare. *Canadian Medical Association Journal, 182*(18), E839-E842. doi:10.1503/cmaj.090449

First 1001 Days Movement. (2020). Our call on Government to keep babies safe [Press release]. Retrieved from https://parentinfantfoundation.org.uk/our-call-on-government-to-keep-babies-safe/

McDonald, H. I., Tessier, E., White, J. M., Woodruff, M., Knowles, C., Bates, C., . . . Edelstein, M. (2020). Early impact of the coronavirus disease (COVID-19) pandemic and physical distancing measures on routine childhood vaccinations in England, January to April 2020. *Eurosurveillance, 25*(19), 2000848. doi:https://doi.org/10.2807/1560-7917.ES.2020.25.19.2000848

Mertz, M., Strech, D., & Kahrass, H. (2017). What methods do reviews of normative ethics literature use for search, selection, analysis, and synthesis? In-depth results from a systematic review of reviews. *Systematic Reviews, 6*(1), 261. doi:10.1186/s13643-017-0661-x

National Voices. (2020). *Five principles for the next phase of the Covid-19 response*. Retrieved from https://www.nationalvoices.org.uk/sites/default/files/public/publications/5_principles_statement_091020.pdf

O'Neill, J., Tabish, H., Welch, V., Petticrew, M., Pottie, K., Clarke, M., . . . Tugwell, P. (2014). Applying an equity lens to interventions: using PROGRESS ensures consideration of socially stratifying factors to illuminate inequities in health. *Journal of Clinical Epidemiology, 67*(1), 56-64. doi:10.1016/j.jclinepi.2013.08.005

Stevens, S., & Pritchard, A. (2020). *Third Phase of NHS Response to Covid-19*. Retrieved from https://www.england.nhs.uk/coronavirus/wp-content/uploads/sites/52/2020/07/20200731-Phase-3-letter-final-1.pdf.

Strech, D., & Sofaer, N. (2012). How to write a systematic review of reasons. *Journal of Medical Ethics, 38*(2), 121-126. doi:10.1136/medethics-2011-100096

Thomas, J., & Harden, A. (2008). Methods for the thematic synthesis of qualitative research in systematic reviews. *BMC Medical Research Methodology, 8*, 1-10. doi:10.1186/1471-2288-8-45

Tong, A., Flemming, K., McInnes, E., Oliver, S., & Craig, J. (2012). Enhancing transparency in reporting the synthesis of qualitative research: ENTREQ. *BMC Medical Research Methodology, 12*(1), 181-181. doi:10.1186/1471-2288-12-181

UK Government. (2017). *Pandemic Flu*. London, UK: UK Government Retrieved from https://www.gov.uk/guidance/pandemic-flu.

For peer review only

FILE 2: PUBLICATION SCHEME SEARCH STRATEGY

The publication scheme search focused on case study hospital Trusts. The focus of the search was the '*How we make decisions'* and *'Our policies and procedures'* sections of the Trust's Publication Scheme. As with the review, sources listed in the publication scheme were excluded if either:

a. they were dated before April 29th, 2020; or
b. their focus and content was on a period prior to April 29th, 2020 (for example an annual report for a financial year to 31st March);

For sources included, a high-level review was then carried out to identify any references to policies or other documents of interest (for example supporting documents or reports prepared for board meetings). The high-level review of included documents was carried out by CR by searching sources for reference to the following terms:

- Covid, Covid-19, coronavirus, SARS-CoV-2, Severe Acute Respiratory Syndrome or pandemic; AND
- Service or care design or delivery, allocation or priority policy, guideline, guidance or framework; OR
  - For paediatric services: Paediatric/pediatric, child/children, critical care, intensive care, acute care, surgery, operation, operating theatre.
  - For maternity services: Maternity, pre-natal, inter-partum, post-natal, perinatal, labour, pregnancy, obstetrics, birth or midwife.

For any sources not accessible through the Trust's publication scheme, Freedom of Information requests were submitted.

For peer review only

FILE 3: THEMATIC ANALYSIS OF PUBLICATION SCHEME CASE STUDY

Publication scheme class	Type of document	Date	Title of document	Themes identified	Sub themes identified
How we make decisions	Board meeting: supporting paper	June 2020	Covid-19 Pandemic – Trust Infection Prevention & Control Response	Respect<br><br>Recognising harms and balancing against benefits (physical, psychological, social and economic) – proportionality<br><br>Reciprocity<br><br>Accountability	collaborative and agile working, patient involvement - eg re-considering place of birth preferences in the context of pressure on emergency ambulance transfer<br><br>staff, patient and visitor safety; testing procedures, agile working, telemedicine, responsiveness - nb availability of abortion medicines at home (no context to this but refs statutory change)<br><br>Staff expected to take care of their own health<br><br>Clear presentation of decisions, rationale, longer term changes to SOP etc.
How we make decisions	Board meeting: supporting paper	June 2020	Update on Covid-19 related Equality Issues	Respect<br><br>Recognising harms etc.<br><br>Reciprocity	Involvement - staff and patients to engage in commms around their care and any specific vulnerabilities identified; collaborative working with staff reps, patient groups etc<br><br>Safety of staff, safety of patients (physical, social, mental wellbeing; specific disadvantages considered - eg non-english speakers; forward planning to mitigate against widening of inequalities<br><br>Mutual exchange, consideration of social, physical and BAME risk factors

For peer review only

				Fairness<br><br>Accountability	Reducing health inequalities, equality impact assessments (EIAs) on all decisions,<br><br>specific governance decisions, implementation detail (eg EIAs), sharing information and clarity of lines of responsibility.
How we make decisions	Board meeting: supporting paper	June 2020	Safeguarding Service Provisions during COVID: Practice-focused document setting out safeguarding practice during Covid - specific to maternity services	Respect<br><br>Recognising harms etc<br><br>Reciprocity<br><br>Fairness<br><br>Accountability	Organised around creating safe spaces for disclosures - eg routine question added during a scan when partner is not present;<br><br>changing ways of working to ensure awareness of abuse is highlighted in practice, focus on patient safety, collaborative working (other agencies - medical and legal),<br><br>Focus is reduction of patient risk<br><br>Everyone matters equally, reduction of social inequalities, disporportionate impact of Covid on this at risk group (NB impact of domestic abuse on staff is also noted<br><br>Built into reporting and governance procedures

For peer review only

ENTREQ Checklist (Tong et al, 2012).

Item	Guide & description	Reported on (section & page no.)
Aim	State the research question the synthesis addresses	p.1, introduction
Synthesis methodology	Identify the synthesis methodology or theoretical framework which underpins the synthesis, and describe the rationale for choice of methodology (e.g. meta-ethnography, thematic synthesis, critical interpretive synthesis, grounded theory synthesis, realist synthesis, meta-aggregation, meta-study, framework synthesis).	p.1, methodology
Approach to searching	Indicate whether the search was pre-planned (comprehensive search strategies to seek all available studies) or iterative (to seek all available concepts until theoretical saturation is achieved).	p.1, methodology and supplementary file 1, rapid review protocol
Inclusion criteria	Specify the inclusion/exclusion criteria (e.g. in terms of population, language, year limits, type of publication, study type).	p.2, inclusion and exclusion criteria
Data sources	Describe the information sources used (e.g. electronic databases (MEDLINE, EMBASE, CINAHL, psychINFO, Econlit), grey literature databases (digital thesis, policy reports), relevant organisational websites, experts, information specialists, generic web searches (Google Scholar), hand searching, reference lists) and when the searches were conducted; provide the rationale for using the data sources.	p. 2, electronic search strategy
Electronic Search strategy	Describe the literature search (e.g. provide electronic search strategies with population terms, clinical or health topic terms, experiential or social phenomena related terms, filters for qualitative research and search limits).	p.2, electronic search strategy
Study screening methods	Describe the process of study screening and sifting (e.g. title, abstract and full text review, number of independent reviewers who screened studies	p.3, screening
Study characteristics	Present the characteristics of the included studies (e.g. year of publication, country, population, number of participants, data collection, methodology, analysis, research questions)	p.4, results, table 2: key characteristics of sources
Study selection results	Identify the number of studies screened and provide reasons for study exclusion (e.g.for comprehensive searching, provide numbers of studies screened and reasons for exclusion indicated in a figure/flowchart; for iterative searching describe reasons for study exclusion	p.4, results and PRISMA flow diagram

For peer review only

	and inclusion based on modifications t the research question and/or contribution to theory development).	
Rationale for appraisal	Describe the rationale and approach used to appraise the included studies or selected findings (e.g. assessment of conduct (validity and robustness), assessment of reporting (transparency), assessment of content and utility of the findings).	p. 3-4, data analysis
Appraisal items	State the tools, frameworks and criteria used to appraise the studies or selected findings (e.g. Existing tools: CASP, QARI, COREQ, Mays and Pope [25];reviewer developed tools; describe the domains assessed: research team, study design, data analysis and interpretations, reporting).	p. 3-4, data analysis
Appraisal process	Indicate whether the appraisal was conducted independently by more than one reviewer and if consensus was required.	p. 3-4, data analysis
Appraisal results	Present results of the quality assessment and indicate which articles, if any, were weighted/excluded based on the assessment and give the rationale.	p.4, results, Table 3: Agree-II assessment of 33 policy guideline sources
Data extraction	Indicate which sections of the primary studies were analysed and how were the data extracted from the primary studies? (e.g. all text under the headings "results /conclusions" were extracted electronically and entered into a computer software).	p. 3, data analysis and Table 1: reset phase coding framework
Software	State the computer software used, if any.	p. 1, electronic search strategy identifies use of EndNote software; and p.2, screening identifies use of Rayyan software
Number of reviewers	Identify who was involved in coding and analysis	p. 2-3, electronic search strategy, screening, and data analysis identify authors involved in each stage
Coding	Describe the process for coding of data (e.g. line by line coding to search for concepts).	p.3, data analysis
Study comparison	Describe how were comparisons made within and across studies (e.g. subsequent studies were	p.3, data analysis

For peer review only

	coded into pre-existing concepts, and new concepts were created when deemed necessary).	
Derivation of themes	Explain whether the process of deriving the themes or constructs was inductive or deductive.	p. 3, data analysis
Quotations	Provide quotations from the primary studies to illustrate themes/constructs, and identify whether the quotations were participant quotations or the author's interpretation.	p. 5-6, results
Synthesis output	Present rich, compelling and useful results that go beyond a summary of the primary studies (e.g. new interpretation, models of evidence, conceptual models, analytical framework, development of a new theory or construct).	p. 6-8, results (table 5, reset phase coding framework inductively developed through the rapid review), and discussion

For peer review only

Which ethical values underpin England's National Health Service reset of paediatric and maternity services following COVID-19: a rapid review.

Journal:	*BMJ Open*
Manuscript ID	bmjopen-2021-049214.R1
Article Type:	Original research
Date Submitted by the Author:	29-Apr-2021
Complete List of Authors:	Chiumento, Anna; University of Liverpool Faculty of Health and Life Sciences, Institute of Population Health Sciences<br>Baines, Paul; University of Warwick, Warwick Medical School<br>Redhead, Caroline; University of Liverpool Faculty of Health and Life Sciences, Institute of Population Health Sciences<br>Fovargue, Sara; Lancaster University, Law School<br>Draper, Heather; University of Warwick, Warwick Medical School<br>Frith, Lucy; University of Liverpool, Institute of Population Health Sciences
**Primary Subject Heading**:	Ethics
Secondary Subject Heading:	Health policy, Health services research, Paediatrics, Public health, Obstetrics and gynaecology
Keywords:	MEDICAL ETHICS, COVID-19, Health policy < HEALTH SERVICES ADMINISTRATION & MANAGEMENT, Protocols & guidelines < HEALTH SERVICES ADMINISTRATION & MANAGEMENT, QUALITATIVE RESEARCH

SCHOLARONE™ Manuscripts

I, the Submitting Author has the right to grant and does grant on behalf of all authors of the Work (as defined in the below author licence), an exclusive licence and/or a non-exclusive licence for contributions from authors who are: i) UK Crown employees; ii) where BMJ has agreed a CC-BY licence shall apply, and/or iii) in accordance with the terms applicable for US Federal Government officers or employees acting as part of their official duties; on a worldwide, perpetual, irrevocable, royalty-free basis to BMJ Publishing Group Ltd ("BMJ") its licensees and where the relevant Journal is co-owned by BMJ to the co-owners of the Journal, to publish the Work in this journal and any other BMJ products and to exploit all rights, as set out in our licence.

The Submitting Author accepts and understands that any supply made under these terms is made by BMJ to the Submitting Author unless you are acting as an employee on behalf of your employer or a postgraduate student of an affiliated institution which is paying any applicable article publishing charge ("APC") for Open Access articles. Where the Submitting Author wishes to make the Work available on an Open Access basis (and intends to pay the relevant APC), the terms of reuse of such Open Access shall be governed by a Creative Commons licence – details of these licences and which Creative Commons licence will apply to this Work are set out in our licence referred to above.

Other than as permitted in any relevant BMJ Author's Self Archiving Policies, I confirm this Work has not been accepted for publication elsewhere, is not being considered for publication elsewhere and does not duplicate material already published. I confirm all authors consent to publication of this Work and authorise the granting of this licence.

For peer review only

Which ethical values underpin England's National Health Service reset of paediatric and maternity services following COVID-19: a rapid review.

Authors:

- Anna Chiumento (corresponding author): Institute of Population Health Sciences, University of Liverpool, Liverpool L69 3BX, United Kingdom; ORCID ID: 0000-0002-0526-0173; Anna.Chiumento@liverpool.ac.uk
- Paul Baines: Warwick Medical School, University of Warwick, Coventry CV4 7AL United Kingdom; ORCID ID: 0000-0001-9045-4054; Paul.Baines@warwick.ac.uk
- Caroline Redhead: Institute of Population Health Sciences, University of Liverpool, Liverpool L69 3BX, United Kingdom; Redhead, Caroline; 0000-0002-7464-2853; C.A.B.Redhead@liverpool.ac.uk
- Sara Fovargue: Law School, Lancaster University, Lancaster, United Kingdom LA1 4YW; 0000-0003-2361-4219; s.fovargue@lancaster.ac.uk
- Heather Draper: Warwick Medical School, University of Warwick, Coventry CV4 7AL United Kingdom; ORCID ID: 0000-0002-0020-4252; H.Draper@warwick.ac.uk
- Lucy Frith: Institute of Population Health Sciences, University of Liverpool, Liverpool L69 3BX, United Kingdom; ORCID ID: 0000-0002-8506-0699; L.J.Frith@liverpool.ac.uk

ABSTRACT (300 words)

Objective: To identify ethical values guiding decision-making in resetting non-COVID-19 paediatric surgery and maternity services in the National Health Service (NHS).

Design: A rapid review of academic and grey-literature sources from 29th April to 31st December 2020, covering non-urgent, non-COVID-19 healthcare. Sources were thematically synthesised against an adapted version of the UK Government's Pandemic Flu Ethical Framework to identify underpinning ethical principles. The strength of normative engagement and the quality of the sources were also assessed.

Setting: NHS maternity and paediatric surgery services in England.

Results: Searches conducted September 8th – October 12th 2020, and updated in March 2021, identified 48 sources meeting the inclusion criteria. Themes that arose include: staff safety; collaborative working – including mutual dependencies across the healthcare system; reciprocity; and inclusivity in service recovery, for example by addressing inequalities in service access. Embedded in the theme of staff and patient safety is embracing new ways of working, such as the rapid roll out of telemedicine. On assessment, many sources did not explicitly consider how ethical principles might be applied or balanced against one-another. Weaknesses in the policy sources included a lack of public and user involvement, and the absence of monitoring and evaluation criteria.

Conclusions: Our findings suggest that relationality is a prominent ethical principle informing resetting NHS non-COVID-19 paediatric surgery and maternity services. Sources explicitly highlight the ethical importance of seeking to minimise disruption to caring and dependent relationships, whilst simultaneously attending to public safety. Engagement with ethical principles was *ethics-lite,* with sources mentioning principles in passing rather than explicitly applying them. This leaves decision-makers and healthcare professionals without an operationalisable ethical framework to apply to difficult reset decisions, and risks inconsistencies in decision-making. We recommend further research to confirm or refine the usefulness of the reset phase ethical framework developed through our analysis.

ARTICLE SUMMARY

Strengths and limitations of this study:

For peer review only - http://bmjopen.bmj.com/site/about/guidelines.xhtml

- The first review to identify the ethical principles guiding decision-making in maternity and paediatric services as England's NHS delivers non-urgent, non-covid-19 healthcare during the pandemic.
- We conducted a rigorous rapid review of sources from policy, academic and grey literature databases.
- Our approach to qualitative synthesis and appraisal of sources against the AGREE-II tool identified areas where ethical guidance and policies lack clarity and fail to implement patient and public involvement.
- Our coding framework is based on the 2017 UK Government Pandemic Flu Ethical Framework, adapted according to two policy sources that met our inclusion criteria, presenting possible methodological tensions.
- An initial Reset Phase Ethical Framework has arisen out of our inductive qualitative synthesis of sources for others to apply and refine.

INTRODUCTION

The coronavirus (COVID-19) pandemic is causing far-reaching consequences for health systems worldwide. In England, the response to the sudden demand for critical care services was to reorient clinical capacity. Many non-urgent services were suspended, and staff and resources redeployed to acute care (1, 2). The pandemic's impact upon routine healthcare has been severe. For example, in England a backlog in areas such as cancer diagnosis and elective surgeries accumulated during the first quarter of 2020 (3, 4). In April 2020, the UK Government declared that non-COVID-19 clinical services **must** resume alongside the capacity for subsequent waves of COVID-19 (5). This 'reset' of NHS services encapsulates all the implications of providing routine care alongside the demands of the coronavirus, including for example the impacts upon caring relationships due to infection prevention and control measures. In this unique 'reset' context it is unclear which ethical values were underpinning decisions about how to reset health services (6). Identifying these acknowledge the role of values in policy-making (7), and recognises that decisions that may appear to be based upon science, resources, or risk are underpinned by value-based judgements (8-10). To identify which ethical values are underpinning reset decision-making in maternity care and paediatric surgery in England we conducted a rapid review of policy, practice and academic sources.

Our review asked: which ethical values (explicitly or implicitly) guided decision-making in non-COVID-19 paediatric surgery (critical/intensive care admissions, surgery, hospital discharge, and aftercare) and maternity services (pre-natal, intrapartum, and post-partum care) during the initial NHS reset in England? We focussed on maternity and paediatric services because professional and patient organisations have highlighted adverse impacts on these areas due to measures to respond to COVID-19 infections (11-14), presenting clear ethical challenges. Maternity services cannot be suspended, and restrictions on accompanying family and carers may have profound effects. We focussed on restarting paediatric surgery because of clear ethical conflicts in the suspension of elective paediatric services even though children are, on the whole, relatively unscathed by COVID-19, and because the secondary effects of the pandemic may have a greater impact on children (15, 16).

The pandemic, with emerging evidence and uncertain outcomes, rapid adjustments to healthcare policies and practices – both for the acute and now the reset phase - and uncertainties around personal risk, has created a particularly challenging decision-making context. The ethical values guiding the resumption of non-COVID-19 health services are likely to differ from the everyday ethical frameworks relied upon prior to the pandemic. The acute phase of the UK's response to the pandemic has been guided by the Pandemic Flu Ethical framework (17), which reorients decision-making from

For peer review only - http://bmjopen.bmj.com/site/about/guidelines.xhtml

an individualised to a more public health ethics orientated approach (18, 19). This ethical framing recognises the relational context of decision-making (20), emphasising mutual dependencies. Notably, the pandemic has disproportionately affected certain social groups (21), including vulnerable older people (22), those with disabilities (23) and Black, Asian and minority ethnic (BAME) communities (24); thus, spotlighting structural inequalities and intersectionalities. It has been proposed that making decisions about healthcare delivery in this context should foreground ethical values such as solidarity (25, 26), reciprocity, and fairness. We aimed to identify which ethical values underpinned decisions about how to reset health services in England (6). This is an important first step in providing an ethical framework for healthcare professionals and decision-makers specific to the reset period (27), and potentially to future pandemics.

METHODOLOGY

We adopted a rapid review methodology appropriate to addressing urgent demands for synthesised evidence (28), conducting a qualitative thematic synthesis (29) following the ENTREQ guidelines (30 - see completed ENTREQ checklist). The protocol guided a comprehensive yet pragmatic approach to the searches, screening, analysis, and appraisal of sources (see supplementary file 1).

Inclusion and exclusion criteria

We included sources that: (a) were developed to guide non-COVID-19 paediatric surgery and maternity services, or (b) discussed the application of ethical values to paediatric surgery and maternity services in England during the reset phase. The reset phase commenced on April 29th 2020, the day NHS services were instructed to prepare delivery of non-COVID-19 surgical services (5), and remains ongoing. Broadly, the reset requires that NHS Trusts:

- resume all non-urgent services incorporating revised COVID-19 infection prevention and control measures;
- prepare for, and manage, second or recurrent waves of COVID-19 infections;
- embrace opportunities to reconfigure health services (e.g. accelerating tele-medicine).

Accordingly, our inclusion criteria were: sources published after 29th April 2020, relating to non-COVID-19 paediatric and maternity services in the NHS in England, discussing decision-making with implicit or explicit reference to ethics, and written in English. A cut-off date of December 31st 2020 was introduced when conducting the updated searches in March 2021, as this is when the Health Foundation COVID-19 policy tracker ended. We took an inclusive approach to data sources which met the inclusion criteria if they were national (UK-wide and applicable to England), NHS Trust, or local policies and directives; guidance or statements from professional bodies; working papers or committee reports; evidence reviews; primary qualitative or quantitative research; peer-reviewed commentaries; or grey literature discussing experiences of paediatric or maternity services in England during the reset phase.

Electronic search strategy

Searches were conducted between 8th September and 12th October 2020 by AC and PB, and updated between 10-21st March 2021 by AC. For academic sources, we searched the bibliographic databases PubMed and PubMed LitCOVID, and clearing houses of COVID-19 related research, including the EPPI Centre Living Map of COVID-19 evidence (31) and Evidence Aid. Recognising the broad scope of our review question, we also searched grey literature sources including websites of UK professional medical bodies (e.g. the Academy of Medical Royal Colleges) and clearing houses of COVID-19 sources, such as the Health Foundation COVID-19 Policy Tracker (32). Additional grey literature and academic websites identified during the search dates were included in an effort to achieve completeness (e.g. 33).

For peer review only - http://bmjopen.bmj.com/site/about/guidelines.xhtml

We developed a search strategy (see supplementary file 1), which was piloted and refined on PubMed (see supplementary file 2). Where search engines did not facilitate MeSH terms, we selected keywords from the list of terms: for example, “paediatric”, “maternity”, or “COVID-19”. For websites where searching was not possible (e.g. 34), a manual review of relevant website sections was undertaken. All grey literature search results were documented in excel spreadsheets or word documents, and bibliographic database searches in EndNote.

Publication scheme and Freedom of Information requests

To complement the electronic searches, we used the Freedom of Information Act 2000 (FOIA (35)) with NHS England Trusts, including those with Clinical Ethics Committees. FOIA imposes two main duties on public authorities: to proactively publish information in a ‘publication scheme’ (36), and to respond to requests for information. We focused on sources such as policies, decision-making tools, Trust board papers and minutes that detailed approaches to ethical decision-making guiding maternity and paediatric services during the reset period. The publication scheme review addressed two classes of information: *‘How we make decisions’* and *‘Our policies and procedures’.* Included documents were read in full and coded against the coding framework by CR (see supplementary file 3). This paper briefly reports a case study example of the publication scheme review.

Screening

Sources were reviewed and duplicates removed before combining results. All were double screened based on title and abstract, where available. Where unavailable, or when undecided, full text review was undertaken. AC, PB, LF, CR, CG and SF screened sources, with HD resolving conflicts in double screening decisions. Papers were categorised against a 0-3 scale, where: 0: not included; 1: included - identifies approach to decision-making; 2: included - identifies what decision has been made; and 3: included – provides justification for decision(s) taken. Where a source met multiple screening categories, all were identified. This categorisation approach sought to provide an initial sense of the depth of sources to inform full-text analysis. Grey literature screening was conducted in a shared excel spread sheet, and for academic sources using Rayyan software (37).

Data analysis

In order to conduct a thematic synthesis of sources, we developed a coding framework for the reset phase. This was based on the Pandemic Flu Ethical Framework (17) adapted according to two interlinked guidance documents: *“Third phase of the NHS response to Covid”,* a letter issued by the NHS Chief Executive and Chief Operating Officer to all NHS Trusts (38), and *“Five Principles for the next phase of the Covid-19 response”*, developed by a coalition of UK health and social care charities (39). The 2017 framework provides a checklist to encourage consideration of the full range of ethical principles in decision-making processes, to guide decisions during a pandemic. We adapted the 2017 framework because it was clear that the reset phase may require a different approach to the acute phase. As part of this adaptation, we reduced the Pandemic Flu Ethical Framework (e.g. removing the principle of “flexibility”, which was viewed as a sub-domain of “minimising harms and balancing against benefits”), and adjusted sub-domains according to how they were operationalised in these two guidance documents (see table 1 for the reset phase coding framework). This adaptation reduced the overlap between principles and sub-domains for application as a coding framework. The resulting framework was iteratively refined through data analysis, as described in the results. Inductive coding involved reading each document and coding against the ethical principles and sub-domains in the coding framework, alongside a 3-5 line summary of the key points from each document and, where relevant, identifying quotes.

Our approach raises a methodological tension as our coding framework draws on two sources relevant to the review, but which were excluded from it. It was, however, justified given the lack of an

For peer review only - http://bmjopen.bmj.com/site/about/guidelines.xhtml

overarching ethical framework tailored to the reset phase, and the need for a coding framework that reflects the ethical specificities of this phase. We will consider this further in the discussion.

TABLE 1: Reset phase coding framework (adapted from the Ethical Framework in the UK Government's Pandemic Flu Policy (17)):

Ethical principle (from Pandemic Flu Ethical Framework)	Adapted sub-domain (based on NHS letter and National Voices Five Principles)
**Respect**	Involvement (i.e. right to express views on matters affecting them, engaging those affected by decisions)
	Respecting choices about personalised care (best interests of person as a whole)
	Collaborative working / engagement (organisational coordination; NHS volunteer scheme, clinical teams, CCGs, local authorities; co-production with voluntary sector, patient orgs etc)
**Recognising harms & balancing against benefits (physical, psychological, social & economic) - proportionality**	Recover operation of healthcare (inc. addressing backlog of care needs, resuming home visits for vulnerable / shielding where appropriate)
	Safety of NHS staff (physical, psychological, systemic inequalities, flexible working)
	Embrace new ways of working (e.g. telemedicine, home visits etc)
	Enhance crisis responsiveness (second wave)
	Accelerate preventative programmes (obesity reduction, seasonal flu, outreach to marginalised groups)
	Responsiveness (adapt plans to new circumstances / information)
**Reciprocity**	Concept of mutual exchange: take responsibility for own behaviour, reduce others expose others to risks
	Protect those at risk of C19 (physically, socially, BAME etc)
**Fairness**	Inclusivity in service recovery (e.g. barriers or access needs, support those with unequal access to care)
	Patient prioritisation (to address backlog i.e. clinical urgent / longest waiting etc)
	Reduce health inequalities (social inequalities & social determinants of health)
	Everyone matters equally & weighted equally in policies & any disproportionate impact on one particular group is accounted for
**Accountability**	Transparency (i.e. document decisions, clarity of who is responsible for decisions, governance arrangements, assess against milestones, sharing information to help others)

Alongside our thematic synthesis, we assessed the extent to which ethical principles were identified, operationalised, and balanced against one another using a 1-3 scale where: (1) ethical principle(s) inferred or mentioned but not clearly applied; (2) ethical principle(s) identified and application described; and (3) ethical principle(s) operationalised, i.e. discussed in-depth, including balancing against other principles. This scoring system was an adaptation of our protocol: we had intended to apply the 'review of reasons' approach (40), but the non-normative nature of the majority of sources rendered this approach unsuitable. Data analysis was led by AC, with PB, CR, SF, LF and CG double coding and scoring 28 sources. Following double coding, the team shared analysis, providing a coding check and discussing emerging findings.

For peer review only - http://bmjopen.bmj.com/site/about/guidelines.xhtml

For peer review only

Policy sources (including professional guidance) were appraised for quality using an adapted version of the AGREE-II instrument (41) reduced to 7 core questions (see table 3). In selecting the quality appraisal questions, we considered the standards that could be anticipated in guideance for which an evidence-base was emerging, and where rapid policy and practice decisions were required (42). Appraisal was conducted independently by AC, PB, SF, CR and CG, drawing upon the criteria defined in the AGREE-II Users Manual (43). This includes scoring of 1-7, where 7: strongly agree (the full criteria are met); 2-6: reporting does not meet the full criteria (lacks completeness or quality of reporting); and 1: strongly disagree (no information, poor reporting of the criteria, or the authors state that criteria were not met).

Patient and public involvement

As this was a rapid review, there was no patient or public involvement.

RESULTS

We present the results of searches, screening, the characteristics of included sources, and the data analysis. We also separately present a case study example of the publication scheme review from one NHS Trust. No FOI responses providing relevant materials were received.

Academic and grey-literature searches identified 19,405 sources (10,505 and 8,900 respectively). After removing duplicates, 18,766 results were screened, with 18,316 excluded as not relevant. 450 sources were assessed for eligibility by title and abstract or, where necessary, full-text screening. Of these, 360 were excluded as being outside the review scope, and upon full text review a further 39 sources were excluded. Therefore, searches identified 48 sources for analysis (see Figure 1).

FIGURE 1: PRISMA flow diagram of searches

Table 2 presents key characteristics of the 48 sources, which include professional guidance (n=30) and statements (n=2), Government policy statements/letters (n=5), academic papers (n=5), reports of patient engagement (n=2) and of implementing professional guidance (n=1), briefing papers (n=2), and a blog post (n=1). Eighteen sources covered all areas of clinical care, 21 focused on maternity services, 8 on paediatric services, and 1 on consent for surgery. The sources covered England or the UK, with some containing Trust-specific case studies. Finally, some sources cross-referenced one another; for example, the Academy of Medical Royal Colleges (44) has accompanying sources focussing on specific areas, such as staff support (45).

For peer review only - http://bmjopen.bmj.com/site/about/guidelines.xhtml

TABLE 2: Key characteristics of sources

Title	Reference	Publication type (policy, report, press release, briefing, statement, professional guidance, peer reviewed article, commentary, decision-support tool / framework, blog)	Date of publication (DD/MM/YY or MM/YY)	Population (Maternity, Paediatrics, or all clinical specialities)	Source scope (international, national, regional, trust, hospital)
**Grey literature sources**					
Principles for reintroducing health services - COVID-19	(44)	Professional guidance	May-20	All	National
Covid-19. Effects on health from non-Covid-19 conditions and moving forward to deliver healthcare for all	(46)	Professional guidance	May-20	All	National
Preparing for COVID-19 surges and winter	(47)	Professional guidance	Jul-20	All	National
Reset, restore and recovery: staff support	(45)	Professional guidance	Jun-20	All	National
Health Protection: Public and professional responsibilities	(48)	Professional guidance	11/07/2020	All	National
Reset, restore and recovery: medical education and training	(49)	Professional guidance	Jun-20	All	National
Reset, restore and recovery: equality	(50)	Professional guidance	Jun-20	All	National
Second phase of NHS response to COVID-19	(51)	Policy (letter)	29/04/2020	All	National
Operating framework for urgent and planned services within hospitals: all emergency patients to be tested on admission and elective patients to isolate for 14 days prior to admission	(52)	Policy	14/05/2020	All	National
Second phase of NHS response to COVID-19 for cancer services	(51)	Policy (letter)	08/07/2020	All	National
WRES briefing for board and COVID-19 emergency preparedness, resilience and response (EPRR) membership in the NHS	(53)	Briefing	24/06/2020	All	National
COVID-19: Guidance for the remobilisation of services within health and care settings, infection prevention and control recommendations	(54)	Public Health England Guidance	20/08/2020	All	National

For peer review only - http://bmjopen.bmj.com/site/about/guidelines.xhtml

Delivering a paediatric elective surgery service during the COVID-19 pandemic	(55)	Implementation of NICE guidance	27/07/2020	All	National
COVID-19: guidance for planning paediatric staffing and rotas	(56)	Professional guidance	10/07/2020	Paediatrics	National
COVID-19 & Us: views from RCPCH & Us	(57)	RCPCH Engagement	04/11/2020	Paediatrics	National
Ethics framework for use in acute paediatric settings during COVID-19 pandemic	(58)	Professional guidance	01/09/2020	Paediatrics	National
National guidance for the recovery of elective surgery in children	(59)	Professional guidance	09/11/2020	Paediatrics	National
Reset, Restore, Recover - RCPCH principles for recovery	(60)	Professional guidance	19/05/2020	Paediatrics	National
It is right to restart services, but we must do so in a safe way	(61)	Blog	07/06/2020	All	National
Antenatal Care for women without suspected or confirmed COVID-19 and living in a symptom free household	(62)	Professional guidance	14/08/2020	Maternity	National
RCM Briefing on Re-introduction of visitors to Maternity Units across the UK during the COVID-19 pandemic	(63)	Professional guidance	15/07/2020	Maternity	National
RCM Clinical Briefing Sheet: guidance for midwifery services on 'freebirth' or 'unassisted childbirth' during the COVID-19 pandemic	(64)	Professional guidance	30/04/2020	Maternity	National
Guidance for the provision of midwife-led settings and home birth in the evolving coronavirus (COVID-19) pandemic	(65)	Professional guidance	21/10/2020	Maternity	National
Equality essentials: Appropriate risk assessment during the current pandemic	(66)	Professional guidance	May-20	Maternity	National
COVID-19 impact on Black, Asian and Minority ethnic (BAME) women	(67)	Professional guidance	15/07/2020	Maternity	National
Principles for the testing and triage of women seeking maternity care in hospital settings during the COVID-19 pandemic: a supplementary framework for maternity healthcare professionals	(68)	Professional guidance	10/08/2020	Maternity	National
Guidance for antenatal and postnatal services in the evolving coronavirus (COVID-19) pandemic	(69)	Professional guidance	19/06/2020	Maternity	National

For peer review only - http://bmjopen.bmj.com/site/about/guidelines.xhtml

Antenatal care for women with current suspected or confirmed COVID-19 or with a member of their household with suspected or confirmed COVID-19	(70)	Professional guidance	24/07/2020	Maternity	National
Domestic Abuse: identifying, caring for and supporting women at risk of/victims of domestic abuse during Covid-19	(71)	Professional guidance	13/11/2020	Maternity	National
Bereavement Care in Maternity Services During COVID-19 pandemic	(72)	Professional guidance	14/07/2020	Maternity	National
Postnatal Care for women with suspected or confirmed COVID-19	(73)	Professional guidance	14/08/2020	Maternity	National
Virtual Consultations	(74)	Professional guidance	24/07/2020	Maternity	National
Restarting planned surgery in the context of the COVID-19 pandemic	(75)	Professional guidance	01/05/2020	All	National
Delivering midwifery intrapartum care where local COVID-19 escalation protocols are required to be enacted	(76)	NICE guidance	20/07/2020	All	National
Supporting pregnant women using maternity services during the coronavirus pandemic: Actions for NHS providers	(77)	Briefing	14/12/2020	Maternity	National
Important – for action – Operational priorities for winter and 2021/22	(78)	Policy (letter)	23/12/2020	All	National
National Clinical Prioritisation Programme (Including Evidence Based Interventions): Frequently asked questions	(79)	Policy	23/09/2020	All	National
Digital by default or digital divide? Virtual healthcare consultations with young people 10-25 years	(80)	Report	Sept 2020	Paediatrics	National
Restoring children's health services, COVID-19 and winter planning - position statement	(81)	Statement	09/10/2020	Paediatrics	National
Anaesthesia and critical care: Guidance for clinical directors on preparation for a possible second surge in COVID-19	(2)	Professional guidance	07/10/2020	All	National
Coronavirus (COVID-19) in pregnancy: information for healthcare professionals	(82)	Professional guidance	14/10/2020	Maternity	National
Joint RCOG & RCM statement: planning for winter 2020/21 - reducing the impact of COVID-19 on maternity services in the UK	(83)	Professional guidance	08/10/2020	Maternity	National

For peer review only - http://bmjopen.bmj.com/site/about/guidelines.xhtml

Midwives call for common sense on maternity visiting guidance	(84)	Statement	15/12/2020	Maternity	National
**Academic sources**					
Implications for the future of obstetrics and Gynaecology following the COVID-19 pandemic: a commentary	(85)	Commentary		Maternity	National
Sustaining quality midwifery care in a pandemic and beyond	(86)	Review article	25/05/2020	Maternity	National
How should surgeons obtain consent during the COVID-19 pandemic?	(87)	BMJ Views and Reviews	30/06/2020	All surgery	National
Professionally responsible advocacy for women and children first during the COVID-19 pandemic: guidance from World Association of Perinatal Medicine and International Academy of Perinatal Medicine	(88)	Peer reviewed article	26/11/2020	Maternity	International
Respectful maternity care in the context of COVID-19: A human rights perspective	(89)	Peer reviewed article	26/11/2020	Maternity	International

For peer review only

Table 3 summarises the assessment of 42 policy /professional guidance against the AGREE-II tool. Sources scored highest for clarity of the guideline objective (19 scored seven, and 10 scored six) and easily identifiable key recommendations (19 scored seven). Favourable scores were achieved for the involvement of professional groups (nine scored seven, and 15 between four and six). Conversely, low scores were common on seeking views of the target population where 24 sources scored one, with three scoring seven; and on whether the guideline presented monitoring and/or auditing criteria, where 25 sources scored one. When assessing whether there was an explicit link between the recommendations and supporting evidence, 21 scored one, with only four scoring seven and one six indicating a clear link. Finally, all sources scored one or two for whether the competing interests of members of the guideline development group had been recorded and addressed.

For peer review only

For peer review only - http://bmjopen.bmj.com/site/about/guidelines.xhtml

TABLE 3: AGREE-II assessment of 42 policy guideline sources

Title	Reference	AGREE-II Questions (domain in brackets)						
		The guideline objective is specifically described (D1)	The guideline development group includes individuals from all relevant professional groups (D2)	The views & preferences of the target population have been sought (D2)	There is an explicit link between the recommendations and the supporting evidence (D3)	Key recommendations are easily identifiable (D4)	The guideline presents monitoring and/or auditing criteria (D5)	Competing interests of the guideline development group members have been recorded & addressed [illegible]
Principles for reintroducing health services - COVID-19	(44)	7	5	1	4	7	1	1
COVID-19. Effects on health from non-COVID-19 conditions and moving forward to deliver healthcare for all	(46)	6	4	3	3	7	1	1
Preparing for COVID-19 surges and winter	(47)	7	4	3	3	7	1	1
Reset, restore and recovery: staff support	(45)	7	4	1	1	7	1	1
Health Protection: Public and professional responsibilities	(48)	7	4	1	1	7	1	1
Reset, restore and recovery: medical education and training	(49)	7	4	3	2	7	1	1
Reset, restore and recovery: equality	(50)	7	4	1	2	7	1	1
Second phase of NHS response to COVID-19'	(5)	7	5	1	3	7	4	1
Operating framework for urgent and planned services within hospitals: all emergency patients to be tested on admission and	(52)	2	1	1	1	7	2	1

peer review only

elective patients to isolate for 14 days prior to admission								
Second phase of NHS response to COVID-19 for cancer services	(51)	1	3	1	2	5	1	1
WRES briefing for board and COVID-19 emergency preparedness, resilience and response (EPRR) membership in the NHS	(53)	4	1	1	3	5	1	1
COVID-19: Guidance for the remobilisation of services within health and care settings, infection prevention and control recommendations	(54)	5	7	1	4	5	1	1
Delivering a paediatric elective surgery service during the COVID-19 pandemic	(55)	7	7	7	7	5	3	1
COVID-19: guidance for planning paediatric staffing and rotas	(56)	7	1	1	1	6	1	1
COVID-19 & Us: views from RCPCH & Us	(57)	7	5	7	7	7	1	1
Ethics framework for use in acute paediatric settings during COVID-19 pandemic	(58)	7	7	1	5	7	3	1
National guidance for the recovery of elective surgery in children	(59)	7	7	5	7	7	4	1
Reset, Restore, Recover - RCPCH principles for recovery	(60)	7	1	1	3	7	1	1
Antenatal Care for women without suspected or confirmed COVID-19 and living in a symptom free household	(62)	5	1	1	5	7	1	1

For peer review only - http://bmjopen.bmj.com/site/about/guidelines.xhtml

RCM Briefing on Re-introduction of visitors to Maternity Units across the UK during the COVID-19 pandemic	(63)	4	1	1	3	3	1	1
RCM Clinical Briefing Sheet: guidance for midwifery services on ‘freebirth’ or ‘unassisted childbirth’ during the COVID-19 pandemic	(64)	5	1	1	4	3	1	1
Guidance for the provision of midwife-led settings and home birth in the evolving coronavirus (COVID-19) pandemic	(65)	6	6	2	5	4	2	2
Equality essentials: Appropriate risk assessment during the current pandemic	(66)	5	3	3	3	5	2	2
COVID-19 impact on Black, Asian and Minority ethnic (BAME) women	(67)	6	4	2	5	4	2	1
Principles for the testing and triage of women seeking maternity care in hospital settings during the COVID-19 pandemic: a supplementary framework for maternity healthcare professionals	(68)	6	3	2	5	5	3	1
Guidance for antenatal and postnatal services in the evolving coronavirus (COVID-19) pandemic	(69)	6	7	2	5	5	3	1
Antenatal care for women with current suspected or confirmed COVID-19 or with a member of	(70)	6	5	2	6	6	2	1

For peer review only - http://bmjopen.bmj.com/site/about/guidelines.xhtml

their household with suspected or confirmed COVID-19								
Domestic abuse: identifying, caring for and supporting women at risk of/victims of domestic abuse during COVID-19	(71)	6	3	3	4	4	2	2
Bereavement Care in Maternity Services During COVID-19 pandemic	(72)	6	4	6	7	3	1	1
Postnatal Care for women with suspected or confirmed COVID-19	(73)	5	7	5	6	4	1	1
Virtual Consultations	(74)	7	5	5	7	6	4	1
Restarting planned surgery in the context of the COVID-19 pandemic	(75)	6	7	1	1	7	1	1
Delivering midwifery intrapartum care where local COVID-19 escalation protocols are required to be enacted	(76)	7	5	1	6	5	1	1
Supporting pregnant women using maternity services during the coronavirus pandemic: Actions for NHS providers	(77)	2	1	2	2	3	1	1
Important – for action – Operational priorities for winter and 2021/22	(78)	5	1	1	1	5	1	1
National Clinical Prioritisation Programme (Including Evidence Based Interventions): Frequently asked questions	(79)	7	1	1	2	7	1	1
Digital by default or digital divide? Virtual healthcare	(80)	7	7	7	1	7	1	1

For peer review only - http://bmjopen.bmj.com/site/about/guidelines.xhtml

consultations with young people 10-25 years								
Restoring children's health services, COVID-19 and winter planning - position statement	(81)	1	1	1	3	4	1	1
Anaesthesia and critical care: Guidance for clinical directors on preparation for a possible second surge in COVID-19	(2)	1	1	1	1	2	1	1
Coronavirus (COVID-19) in pregnancy: information for healthcare professionals	(82)	6	2	3	4	5	2	2
Joint RCOG & RCM statement: planning for winter 2020/21 - reducing the impact of COVID-19 on maternity services in the UK	(83)	7	7	1	7	7	2	1
Midwives call for common sense on maternity visiting guidance	(84)	7	6	1	7	7	2	1

For peer review only

For peer review only - http://bmjopen.bmj.com/site/about/guidelines.xhtml

Table 4 summarises the qualitative thematic synthesis of all 48 sources, highlighting the frequency of coding to each sub-domain, and scores for the operationalisation of ethical principles.

For peer review only

TABLE 4: Thematic analysis of sources

**THEMATIC ANALYSIS**		
**Principles**	**Sub-domains**	**References**
**Respect**	Involvement	(44, 46, 47, 49, 52, 53, 57-60, 63-65, 67-74, 77, 79-82, 84, 85, 87-90)
	Respecting choices about personalised care	(44, 58-60, 64, 65, 67, 69, 70, 72, 74, 77, 78, 80, 82, 83, 87, 88)
	Collaborative working / engagement	(2, 5, 44, 46, 47, 52-56, 59, 60, 62-65, 69, 71-73, 75, 76, 78, 81, 83-85, 90)
**Recognising harms & balancing against benefits (physical, psychological, social & economic) - proportionality**	Recover operation of healthcare	(2, 5, 44-47, 50, 52, 55-58, 60-63, 65, 68-73, 75, 77-79, 81-84, 87, 89, 90)
	Safety of NHS staff	(2, 5, 44-46, 48-50, 52-56, 59-61, 63, 65-71, 73-78, 81-86, 88, 90)
	Embrace new ways of working	(5, 44, 47, 53-55, 57, 59, 60, 62, 64, 65, 67-72, 74, 77, 78, 80-82, 85, 86, 89)
	Enhance crisis responsiveness	(2, 5, 44, 45, 47, 58, 59, 61, 84)
	Accelerate preventative programmes	(2, 5, 46, 47, 67, 71, 81, 83, 85)
	Responsiveness	(2, 53, 56-60, 62, 64, 65, 68-73, 75-77, 80-82, 84, 88)
	Patient safety	(2, 46, 48, 52, 55, 58, 59, 61, 62, 64, 65, 67-71, 77, 79-84, 86-89)
**Reciprocity**	Mutual exchange	(47, 48, 54, 56, 59, 63, 67, 70, 77, 84)
	Protect those at risk of COVID-19	(2, 5, 44, 46-48, 50, 52-57, 59, 62, 63, 65, 66, 68-70, 73, 75, 82, 86)
**Fairness**	Inclusivity in service recovery	(2, 44, 46, 50, 57, 62, 65, 67, 69, 71-75, 77, 80-85, 90)
	Patient prioritisation	(2, 5, 44, 46, 50, 52, 58, 59, 61, 67, 75, 78, 79, 81, 88)
	Reduce health inequalities	(50, 53, 57, 60, 62, 66, 67, 69, 71, 73, 74, 77, 78, 80-83, 85, 89)
	Everyone matters equally	(2, 53, 58, 59, 61, 63, 64, 67, 68, 71, 75, 77, 80-82, 84, 85, 88, 89)
**Accountability**	Transparency	(5, 46, 47, 50, 52-54, 57-60, 63-65, 71, 74, 77-81, 83, 84, 88)
	Finance	(5, 78, 85)
	Sustainability	(81, 83)
**JUSTIFICATION OF PRINCIPLES**		
**1**	Principle(s) inferred or mentioned, but not clearly applied	(5, 44-48, 50, 52, 57, 59, 60, 66, 67, 70, 73, 76-79, 82, 84, 90)
**2**	Application of principle(s) described	(49, 53-56, 61-64, 68, 69, 71, 72, 74, 80, 83, 85, 87, 89)
**3**	Application of principle(s) discussed in-depth, including balancing against other principle(s)	(2, 58, 65, 75, 81, 86, 88)

For peer review only - http://bmjopen.bmj.com/site/about/guidelines.xhtml

All sources explicitly referenced or applied the principle of recognising harms and balancing these against possible benefits. The sub-domain of *safety of NHS staff* was most frequently coded, with *recovering the operation of healthcare* and *embracing new ways of working* explicitly identified slightly less frequently. Staff safety was understood broadly, encompassing PPE, testing and isolation protocols, the importance of staff wellbeing (including leave), and the importance of ongoing staff training (2, 46, 49, 56, 61). Concerns about staff training and progression became more prominent as the pandemic continued to cause disruption (2, 45) New ways of working frequently identified telemedicine, an approach that had been effectively applied in remote community maternity care prior to the pandemic (74). Integrating telemedicine was recommended in the context of trusting relationships built through in-person care (69) which involved individualised assessments of patients' characteristics and life circumstances (74), such as the need for interpretation services (62), and confidentiality concerns (57). Both maternity and paediatric sources reflected potential risks with virtual care in relation to *"unvoiced concerns"* (82), recommending a low threshold for in-person consultations (80). In resetting health services, it was anticipated that routine care would resume in a non-linear way (75); therefore, continuing adaptation to the evolving situation would be required (2, 62), including establishing new *"post-Covid assessment Services"* (78). To support this, risk management tools and service level models were proposed (2) that accounted for impacts upon key areas, such as human resources (59, 62), or sample risk assessments with recommended phases; for example, for reintroducing visitors and sample visiting guidelines (63, 77). Caution against resuming planned healthcare and routine visiting too quickly was advised due to the time and effort required to reorient people and equipment to routine roles, and the additional demands of safety and infection control (61, 84). Once re-established, the need to protect routine services from the potential impact of subsequent waves of COVID-19 in the paediatric context was emphasised to avoid further risks to child health as a result of delayed care (81).

Respect was a frequently explicitly considered principle, encompassing keeping people informed and respecting personal decisions about care, including acknowledging patients' right to express views on matters affecting them both directly and through organisations such as the Maternity Voices Partnership (77). Examples of such involvement included using patients' experiences of lockdown to inform plans for maintaining routine care alongside managing the coronavirus (57). Paediatric sources were notable for high levels of involvement (57, 80), with one including young people's definition of the concept of reset, encompassing *"contact, connections, and interactions with patients"* whilst accounting for individual needs and circumstances (81). The use of active public health messaging or outreach to involve patients was also identified (46, 59, 81, 85), and was added to the coding framework as a sub-domain of respect.

Collaborative working was explicitly referenced, recognising the co-dependency of elements of the health service*: "turning on the tap at one end will not necessarily release the flow at the other — there are multiple taps which need to be released in a sequential fashion"* (46). Embedding collaboration across hospitals and Trusts was called for through local, regional and national coordination, the redeployment of staff across specialities, the accelerated qualification of students, and the return of retired staff who had supported human resource capacity during the first wave of COVID-19 (5, 46). Over time, the impact of redeployment on the capacity to provide routine services was considered, including the need for some staff to be protected: *"Maternity staff cannot be replaced by other staff groups due to their specialist skill set and protecting this workforce from unnecessary risk is therefore crucial to ensure that maternity care can be sustained"* (83), and protecting routine child health services from adult COVID-19 escalation processes (81).

For peer review only - http://bmjopen.bmj.com/site/about/guidelines.xhtml

Inclusivity in service delivery was emphasised under the principle of fairness. Barriers to maternity care such as English language abilities, immigration status, and individualised factors - including risk of domestic abuse or history of human trafficking - were identified (67, 71, 82). This sub-domain was frequently considered alongside explicit recognition that everyone matters and should be considered equally in policies. For example: *"…it is important to consider the needs of surgical patients on an equal footing with those receiving care for COVID-19 and other medical diseases"* (75). Sources identified in the updated searches introduced processes for patient prioritisation for elective care (79) and the concept of *"timely and safe discharge"* to maximise the capacity to respond to ongoing waves of COVID-19 infections (78). Conducting Equality Impact Assessments to ensure rapid adjustments of policies and procedures to address inequalities and meet public duties was also noted (5, 63).

Under the principle of reciprocity, the sub-domain of everyone taking actions to protect healthcare workers and patients was explicitly emphasised. Notably, this recognised the increased risks and burdens faced by healthcare staff and those at increased risk of COVID-19 infection and poor outcomes, such as members of BAME communities (47, 50, 53, 82). Finally, accountability was implicitly reflected in the sub-domain of transparency, with explicit reference to documenting decisions (58, 64, 74, 79) and engaging in monitoring, evaluation (59), and research (5, 47); and calls for continuing data collection and patient involvement to inform policy- and decision-making (80). Transparency in governance structures and decision-making processes were also underscored (3), thereby ensuring adherence to the UK Equalities Act 2010. Sustainability of both NHS resources (such as staffing) and environmental sustainability (notably in relation to disposable PPE) were added to the coding framework as a sub-domain emerging from the updated searches (81, 83).

The analysis led to iterative inductive evolution of the coding framework, adding sub-categories identified in italics in table 5, which form the ethical framework emerging from this review.

For peer review only - http://bmjopen.bmj.com/site/about/guidelines.xhtml

1 **TABLE 5: Reset phase ethical framework inductively developed through the review (adapted from**
2 **the UK Government's Pandemic Flu Policy Ethical Framework (17))**

**Ethical principle (from Pandemic Flu Ethical Framework)**	**Sub-domain**
**Respect**	Involvement (i.e. right to express views on matters affecting them, engaging those affected by decisions, *active communication / outreach including public health messaging*)
	Respecting choices about personalised care (best interests of person as a whole *including decisions in best interests of children and young people*)
	Collaborative working / engagement (organisational coordination *including redeployment*; NHS volunteer scheme, clinical teams, CCGs, local authorities, *Nightingale & independent hospitals*; co-production with voluntary sector, patient orgs, *equality, diversity and inclusion of the workforce* etc.)
**Recognising harms & balancing against benefits (physical, psychological, social & economic) - proportionality**	Recover operation of healthcare (including addressing backlog of care needs, resuming home visits for vulnerable / shielding where appropriate; *resources (staffing, spaces and equipment*)
	Safety of NHS staff (physical, psychological, systemic inequalities, flexible working, *meeting staff training needs*)
	Embrace new ways of working (e.g. telemedicine, home visits, *COVID-19 testing protocols, pathways for low- and high-risk care*)
	Enhance crisis responsiveness (second wave)
	Accelerate preventative programmes (obesity reduction, seasonal flu, outreach to marginalised groups, *antenatal and postnatal care*)
	Responsiveness (adapt plans to new circumstances / information)
	Patient safety (individualised risk protocols and support person / visiting protocols)
**Reciprocity**	Concept of mutual exchange: take responsibility for own behaviour, reduce others expose others to risks
	Protect those at risk of COVID-19 (physically, socially, BAME etc)
**Fairness**	Inclusivity in service recovery (e.g. barriers or access needs, support those with unequal access to care)
	Patient prioritisation (to address backlog i.e. clinical urgent / longest waiting, *option of continuing to wait & postpone treatment, "reason to reside" criteria for timely and safe discharge*)
	Reduce health inequalities (social inequalities & social determinants of health)
	Everyone matters equally & weighted equally in policies & any disproportionate impact on one particular group is accounted for
**Accountability**	Transparency (i.e. document decisions, clarity of who is responsible for decisions, governance arrangements, assess against milestones, sharing information to help others)
	Finance
	Sustainability (of NHS services [e.g. staffing]; environmental sustainability)

3

For peer review only - http://bmjopen.bmj.com/site/about/guidelines.xhtml

Scoring sources for their practical usefulness to healthcare professionals highlights that nearly half explicitly identified key ethical principles but failed to offer advice about how they might be used in decision-making (22 scored one). Broad statements about core principles were often made, such as respect for patients and minimising harms which were frequently mentioned in relation to infection prevention and control. Nineteen sources scored two for clearly identifying ethical principles and suggesting how they might be applied; for example, by identifying decision-making support tools (e.g. (63)). Seven sources scored three for their focused, practical suggestions regarding the application of the identified ethical principles, often balancing them against one another. For example, the ethical framework for acute paediatric settings (58) balanced treatment prioritisation against resource constraints, identified decision-making tools, and engaged with case scenarios to illustrate ethical tensions, such as the disruptions to care pathways for children with complex needs. It is notable that there was no clear correlation between the quality appraisals against the AGREE-II tool and depth of ethical engagement.

Publication scheme case study

We present initial findings from one NHS Trust publication scheme review (see supplementary file 4). As with the wider review findings, the Trust board's focus was on patient, staff, and visitor safety, including broad concern with the effects of the Trust's decision-making on service delivery during the reset period. An example from a maternity service was the creation of a safe space for disclosure of domestic violence by making a small, but important, adjustment to Trust Standard Operating Procedures by adding questions to ask when a pregnant person's partner was not present. This example reflects an awareness of patients' increased exposure to domestic violence as a result of lockdown, demonstrating the benefit of paying attention to ethical considerations including inequality and patient safety in a specific decision-making context.

DISCUSSION

Our pragmatic rapid review identified the ethical principles referenced in published academic and grey literature and decision-making guidance informing the resetting of NHS paediatric surgery and maternity services. A key review outcome is a reset phase ethical framework inductively developed based upon the sources reviewed (Table 5). Our results indicate high levels of congruence in the key ethical considerations and areas of ethical tension underpinning the resetting of both maternity and paediatric services. In this discussion, we focus on two areas of ethical distinctiveness in the reset: the ways that relationality was invoked, and the emphasis on equity. We also consider the practical usefulness of the included sources for healthcare professionals applying to concrete situations (91), and outline how the reset ethical framework developed through this review might be operationalised.

Relationality was reflected in numerous ways, anchored in the individual and organisational mutual dependencies and responsibilities that have been starkly highlighted by the coronavirus pandemic. The ethical importance of attending to the adverse impact of the coronavirus on caring and dependent relationships, seeking to minimise disruption to these as much as possible to meet the needs of patients and family or carers, whilst simultaneously attending to public safety is one example. In our review, the relational context of decision-making was prominent, reflecting family and caring relationships inherent to our areas of focus: birthing partners in maternity care, and parents or carers in paediatric services (58, 72, 77, 84). Explicit steps to minimise harms and maximise staff and patient safety were grounded in risk assessment and infection prevention and control protocols that relied upon reciprocal responsibilities. Reciprocity was also explicitly identified in the additional protections for those at risk of adverse outcomes from COVID-19 due to systematic inequalities and intersectionalities (21, 82). The importance of balancing infection prevention and control actions to reduce COVID-19 transmission with other risks to healthcare was explicitly recognised; notably

For peer review only - http://bmjopen.bmj.com/site/about/guidelines.xhtml

acknowledging the potential emotional impacts for patients attending appointments or giving birth alone. Psychological safety was reflected in explicit calls to attend to the emotional impacts of delivering care during the pandemic and to minimise the risk of staff burnout. Finally, relationality was implicit in inter-organisational collaboration locally, regionally and nationally to coordinate continuity of care, emphasising co-dependencies of different areas of the health service (92). A distinctive focus on health equity was explicit in sources balancing the needs of those with COVID-19 with those requiring routine healthcare. Health equity was also implicitly reflected in calls for pro-active outreach to overcome health inequalities and ensure care was accessed when needed, including public health measures such as immunisation campaigns attending to potential inequalities of access.

Our assessment of the level of engagement with ethical principles found them to be 'ethics-lite'. Whilst key principles were referenced, sometimes only in passing, many sources failed to operationalise them. We define operationalisation as applying ethical principles to specific situations, considering how predictable ethical dilemmas might be managed, or offering suggestions as to how, in practice, ethical principles might be balanced against one another. This is especially important when the ethical approach moves between individual-focussed clinical care and wider public health measures which is recognised to produce a *"jarring and unwelcome"* (p.871) shift in ethical framing that clinicians must negotiate (88). In recognising this, we do not call for prescriptive guidance for every circumstance; rather, that guidance should inform and constrain the judgements of those applying them (91). To achieve this *how* they ought to be operationalised must be clear. Guidance lacking this dimension leave healthcare professionals without a coherent ethical framework to support decision-making (27), which can result in moral distress (93). Moreover, *"Research in psychology has demonstrated that when people are working in stressful situations under pressure of time, with access to extensive yet conflicting information from multiple sources, and when outcomes are uncertain, they tend to make more decisions based on intuition, gut feelings, or heuristics (rules of thumb) rather than on rational thinking (Kahneman, 2011)*" (85, p.2). This exactly describes the COVID-19 context, with emerging evidence and uncertain outcomes, rapid adjustments to healthcare policies and practices – both for the acute and the reset phase - and uncertainties around personal risk. In such situations, consistently interpreting and applying broad-brush ethical guidance to practice becomes impossible. A clear ethical framework to underpin decision-making is therefore required (91, 94).

Our reset ethical framework, inductively developed through this review, offers a useful starting point. Additional research is required to confirm or further refine its congruence with the decision-making processes of individual Trusts and healthcare providers - embedded within their regional and systemic relationships, and to areas of healthcare beyond paediatric surgery and maternity services. This forms part of our ongoing research activities. Recognising the importance of our review finding that ethical frameworks should be operationalisable, we briefly explain how our reset ethical framework could be applied in practice. The Pandemic Flu Ethical Framework emphasises *equal concern and respect* as the underpinning principle (95), which is echoed in our review where *fairness*, chiefly that *everyone matters equally and is weighted equally*, has emerged as an underpinning principle. However, our review demonstrates that the NHS operational context in the reset is ethically distinct. The underpinning principle of fairness must be balanced across considerations such as the impact of delayed care; constraints of infection prevention and control measures; broad mutual inter-dependencies between healthcare providers, patients and the public; and uncertain COVID-19 risks – exacerbated by inequalities and intersectionalities - for healthcare providers and patients. These considerations foreground complex, layered configurations of interdependencies and relationships embedded within healthcare provision in the reset. Ethical frameworks may assist decision-makers to navigate this challenging decision-making context. Consequently, in contrast to the UK Chief

For peer review only - http://bmjopen.bmj.com/site/about/guidelines.xhtml

Medical Officers advice not to produce updated ethical guidance for the coronavirus pandemic (96), our review indicates that the ethically distinctive COVID-19 healthcare operational context *urgently* requires a tailored approach (97). We agree with the Scottish Government (98) that such a framework should be operationalised to support organisational and individual-level decision-making at national, regional and local levels; for example, through Trust specification (see e.g. 99) and with the pragmatic advice and consultation of Clinical Ethics Committees, and, where relevant, patient involvement groups.

Appraising sources against the AGREE-II tool identified a lack of monitoring and auditing systems for rapidly adjusted policies and practice guidance, which is concerning given the reported impacts on some areas of patient care. It also showed a lack of public involvement beyond, at best, patient representatives (73), and a lack of transparency around potential competing interests in guidance development. The Government's Phase two letter provided Trusts the short timeline of 21 weeks to design their service reset (5). Engagement processes, already time consuming, had to be adapted to online formats. It is, therefore, not surprising that public involvement was lacking. However, in March 2020 NHS England restated the statutory, and ethical, duty to maintain public involvement in decisions about service provision (100), suggesting that this should have taken place. Public involvement is fundamental to public trust in the collective actions of the NHS and the standards of professional ethical practice of individual health care providers (101-103). This is essential to meet the NHS Constitution's guiding principle, that "*the NHS is accountable to the public, communities and patients that it serves*" (104). As such, public and patient involvement provides an important moral foundation for difficult ethical decisions in the reset phase and beyond (105).

Our review maintained methodological rigour by including a systematic search strategy where possible, and double screening and double coding 25% of sources. Team discussions to develop the coding framework and reflect on emerging findings were also ongoing throughout. We adopted an inclusive approach to grey literature and academic sources, ensuring the relevance of our review to healthcare policy and practice. This was complemented by the publication scheme review, which indicated the application of guidelines to situated Trust-level decision-making. However, methodological limitations remain, chiefly that the rapidity of the review rapidity necessarily limited its scope and depth (42), and may not have identified all relevant sources. Time constraints prevented a multiple appraisal of policy sources as recommended by the AGREE-II tool (43). Where double coding arose as a result of a source being revised and included in updated searches, some discrepancies arose in AGREE-II appraisals, which were managed by awarding the highest scores. Time constraints also meant that only CR analysed the publication scheme data. A key methodological challenge in this review was the tension in developing the coding framework from two sources that met the review inclusion criteria. We believe this was acceptable given the inductive and iterative thematic synthesis approach, which led to the inductive development of a revised framework that reflects the distinctive considerations facing decision-makers and clinicians during the reset phase. Finally, the breadth of our review question made the adoption of approaches designed for normative reviews challenging, and resulted in the use of a scoring system that accommodated our review scope.

This review has sought to render explicit the ethical values underpinning decision-making specific to the reset phase, yielding important learning for healthcare policy makers and Trust decision-makers. Our findings suggest that some key ethical and legal duties – such as involvement – have been immediate casualties of the time-pressured decision-making context. We accept that there may be significant logistical barriers to achieving meaningful engagement, and that compromises during a crisis may be required (17). However, we recommend that guidance is transparent about any lack of involvement and the reasons for this, whilst seeking to re-establish meaningful engagement as quickly

For peer review only - http://bmjopen.bmj.com/site/about/guidelines.xhtml

as possible. We are encouraged that updated searches identified increased involvement of patients, notably informing the resumption of paediatric services (81) and promoting the role of patient representative organisations such as the Maternity Voices Partnership (77). We also recommend that those developing policy and practice guidance pay attention to their practical application. This will ensure that any normative decision-making is operationalisable in the context in which healthcare professionals are working.

CONCLUSION

This review adds to the rapidly evolving evidence on England's health systems' response to the coronavirus pandemic, focussing on the normative foundations underpinning the resetting of NHS health services in maternity and paediatric surgery services, alongside a continuing response to the demands of COVID-19. It is important that the government and professional bodies continue to engage with the difficult ethical decisions this requires, and we recommend increased public involvement in this process to build solidarity in supporting the required responses. Our review has found that to date, guidance developed for this period are ethics-lite and fail to provide an operationalisable ethical framework for decision-makers and healthcare professionals. Addressing this is an important priority as the NHS in England moves further into the reset period, where difficult ethical decisions about *how* health services resets will continue to be necessary. We are supporting this process by publishing our proposed reset ethics framework here. This has been inductively developed based upon the sources included in this review. We continue to refine this framework through our ongoing empirical and conceptual research.

AUTHORS CONTRIBUTIONS STATEMENT

LF, HD, AC, SF and PB designed the rapid review concept, question, and protocol. AC, LF, HD, SF, PB and CR were involved in various stages of conducting the review, as specified in the paper. All authors were involved in regular team meetings to discuss and reflect upon review conduct and emerging findings. AC led the writing of the paper, with all authors providing review and feedback, and approving the final version for publication.

COMPETING INTERESTS STATEMENT

The authors declare they have no competing interests.

FUNDING STATEMENT

This rapid review is the first phase of the *Everyday and Pandemic Ethics* project (https://www.liverpool.ac.uk/population-health-sciences/departments/health-services-research/key-projects/resetethics/) funded by the UKRI AHRC Covid-19 rapid response call [AH/V00820X/1]

DATA SHARING STATEMENT

Additional data available upon reasonable request to the corresponding author.

ACKNOWLEDGEMENTS

The authors thank Dr Rui Hill, University of Liverpool for his helpful guidance on early stages of developing the review protocol, suggestions for review software, and support with writing up the review search strategy. We also thank Dr Diego Silva, Melbourne University, for his feedback on an earlier draft of this paper as discussant at an Australasian Association of Bioethics in Health and Law workshop in December 2020; and the helpful reflections of other members of the workshop session. Finally, our thanks to Dr Carol Gray [CG], University of Liverpool, who contributed to the data analysis, quality assessment, and data interpretation of the sources included in the updated March 2021 searches.

For peer review only - http://bmjopen.bmj.com/site/about/guidelines.xhtml

MAIN MANSUCRIPT WORD COUNT: 5,373 words.

REFERENCES

1. Academy of Medical Royal Colleges. NHS Health and Social Care Committee: Delivering core NHS and care services during the pandemic. London, UK: Academy of Medical Royal Colleges; 2020. https://www.aomrc.org.uk/wp-content/uploads/2020/07/200630_NHS_health_social_care_committee_submission.pdf. (Access date: 03.11.2020).
2. Intensive Care Medicine, Intensive Care Society, Association of Anaestetists*, et al.* Anaesthesia and critical care: guidance for Clinical Directors on preparation for a possible second surge in COVID-19. London, UK; 2020. https://icmanaesthesiacovid-19.org/anaesthesia-and-critical-care-second-surge-guidance. (Access date: 05.11.2020).
3. National Health Service. Implementing phase 3 of the NHS response to the Covid-19 pandemic. 2020. https://www.england.nhs.uk/wp-content/uploads/2020/08/C0716_Implementing-phase-3-v1.1.pdf. (Access date: 29.09.2020).
4. Gardner T, Fraser C, Peytrignet S. Elective care in England: Assessing the impact of COVID-19 and where next. The Health Foundation; 2020. https://www.health.org.uk/publications/long-reads/elective-care-in-england-assessing-the-impact-of-covid-19-and-where-next. (Access date: 25.11.2020).
5. Stevens S, Pritchard A. Second phase of NHS response to COVID-19. 2020. https://www.england.nhs.uk/coronavirus/wp-content/uploads/sites/52/2020/04/second-phase-of-nhs-response-to-covid-19-letter-to-chief-execs-29-april-2020.pdf. (Access date:
6. NHS Confederation. Health and Social Care Select Committee inquiry on delivering core NHS and care services during the pandemic and beyond: written evidence from the NHS Confederation. 2020. https://www.nhsconfed.org/-/media/Confederation/Files/Public-Affairs/NHS-Confederation-submission-to-inquiry-on-Delivering-Core-NHS-and-Care-Services-during-the-Pandemic.pdf. (Access date: 03.11.2020).
7. Nuffield Council on Bioethics. Spotlight briefing: Ten questions on the next phase of the UK's Covid-19 response. London: Nuffield Council on Bioethics, . 2020. https://www.nuffieldbioethics.org/assets/pdfs/Ethical-considerations-in-the-next-phase-of-the-COVID-19-response.pdf. (Access date: 17.11.2020).
8. Archard D, Whitall H. Statement: the need for national guidance on resource allocation decisions in the COVID-19 pandemic. London: Nuffield Council on Bioethics, . 2021. https://www.nuffieldbioethics.org/news/statement-the-need-for-national-guidance-on-resource-allocation-decisions-in-the-covid-19-pandemic. (Access date: 14.01.2021).
9. Parker M. Ethical and value judgements involved in developing policy for lifting physical distancing measures. 2020. London. UK Government. https://assets.publishing.service.gov.uk/government/uploads/system/uploads/attachment_data/file/890173/s0263-ethics-emerging-from-lockdown-290420-sage30.pdf. (Access date: 05.08.2020).
10. Archard D. "Following the science" in the COVID-19 pandemic. London: Nuffield Council on Bioethics, . 2020. [cited 2020 05.05.2020]. Available from: https://www.nuffieldbioethics.org/blog/following-the-science-in-the-covid-19-pandemic. (Access date: 05.05.2020).
11. McDonald HI, Tessier E, White JM*, et al.* Early impact of the coronavirus disease (COVID-19) pandemic and physical distancing measures on routine childhood vaccinations in England, January to April 2020. Eurosurveillance. 2020;25(19):2000848. https://doi.org/10.2807/1560-7917.ES.2020.25.19.2000848.
12. First 1001 Days Movement. Our call on Government to keep babies safe [press release]. 2020. https://parentinfantfoundation.org.uk/our-call-on-government-to-keep-babies-safe/. (Access date: 10.09.2020).

For peer review only - http://bmjopen.bmj.com/site/about/guidelines.xhtml

13. Anonymous. Reflections on Covid restrictions from a pregnant midwife. Make Births Better2020. [25.11.2020]. Available from: https://www.makebirthbetter.org/blog/reflections-on-covid-restrictions-from-a-pregnant-midwife. (Access date: 25.11.2020).
14. Association of Paediatric Anaesthetists of Great Britain and Ireland A. APAGBI position statement regarding the delivery and recovery of Children's surgery during the coronavirus pandemic [press release]. 2020. https://www.apagbi.org.uk/sites/default/files/inline-files/APA%20statement%2015.05.20%20Final.pdf. (Access date: 25.11.2020).
15. UNICEF. COVID-19 and children 2020 [Available from: https://data.unicef.org/covid-19-and-children/. (Access date: 09.01.2021).
16. Bogiatzopoulou A, Mayberry H, Hawcutt DB*, et al.* COVID-19 in children: what did we learn from the first wave? Paediatrics and Child Health. 2020;30(12):438-43. 10.1016/j.paed.2020.09.005.
17. UK Government. Pandemic Flu. 2017. London, UK. UK Government. https://www.gov.uk/guidance/pandemic-flu. (Access date: 24.06.2020).
18. Baines P, Draper H, Chiumento A*, et al.* COVID-19 and beyond: the ethical challenges of resetting health services during and after public health emergencies. Journal of Medical Ethics. 2020;46(11):715-6. 10.1136/medethics-2020-106965.
19. Berlinger N, Wynia M, Powell T*, et al.* Ethical Framework for Health Care Institutions Responding to Novel Coronavirus SARS-CoV-2 (COVID-19): Guidelines for Institutional Ethics Services Responding to COVID-19. The Hastings Centre; 2020. https://www.thehastingscenter.org/wp-content/uploads/HastingsCenterCovidFramework2020.pdf. (Access date: 25.11.2020).
20. Dove ES, Kelly SE, Lucivero F*, et al.* Beyond individualism: Is there a place for relational autonomy in clinical practice and research? Clinical Ethics. 2017;12(3):150-65. 10.1177/1477750917704156.
21. Horton R. Offline: COVID-19 is not a pandemic. The Lancet. 2020;396(10255):874. 10.1016/S0140-6736(20)32000-6.
22. Public Health England. Disparities in the risk and outcomes of COVID-19. London: Public Health England; 2020. https://assets.publishing.service.gov.uk/government/uploads/system/uploads/attachment_data/file/908434/Disparities_in_the_risk_and_outcomes_of_COVID_August_2020_update.pdf. (Access date: 17.12.2020).
23. Office for National Statistics. Coronavirus and the social impacts on disabled people in Great Britain: September 2020. London: UK Government, . 2020. https://www.ons.gov.uk/peoplepopulationandcommunity/healthandsocialcare/disability/articles/coronavirusandthesocialimpactsondisabledpeopleingreatbritain/september2020. (Access date: 18.01.2021).
24. Public Health England. Beyond the data: understanding the impact of COVID-19 on BAME groups. London: Public Health England; 2020. https://assets.publishing.service.gov.uk/government/uploads/system/uploads/attachment_data/file/892376/COVID_stakeholder_engagement_synthesis_beyond_the_data.pdf. (Access date: 17.12.2020).
25. West-Oram P. Solidarity is for other people: identifying derelictions of solidarity in responses to COVID-19. Journal of Medical Ethics. 2020. 10.1136/medethics-2020-106522.
26. Prainsack B, Buyx A. Solidarity: reflections on an emerging concept in bioethics. Nuffield Council on Bioethics; 2011. https://www.nuffieldbioethics.org/assets/pdfs/Solidarity-report.pdf. (Access date: 03.12.2020).
27. Coggon J, Regmi S. Covid-19: Government guidance on emergency rationing of critical care is needed to support professional decision making [Internet]. BMJ Opinion2020. [24.10.2020]. Available from: https://blogs.bmj.com/bmj/2020/04/24/covid-19-government-guidance-on-emergency-rationing-of-critical-care-is-needed-to-support-professional-decision-making/. (Access date: 24.10.2020).

For peer review only - http://bmjopen.bmj.com/site/about/guidelines.xhtml

28. Khangura S, Konnyu K, Cushman R, *et al.* Evidence summaries: the evolution of a rapid review approach. Systematic Reviews. 2012;1(1):10. 10.1186/2046-4053-1-10.
29. Thomas J, Harden A. Methods for the thematic synthesis of qualitative research in systematic reviews. BMC Medical Research Methodology. 2008;8:1-10. 10.1186/1471-2288-8-45.
30. Tong A, Flemming K, McInnes E, *et al.* Enhancing transparency in reporting the synthesis of qualitative research: ENTREQ. BMC Medical Research Methodology. 2012;12(1):181-. 10.1186/1471-2288-12-181.
31. EPPI-Centre. Covid-19: a living systematic map of the evidence UCL: Institute of Education; 2020 [updated 08.01.2021. Available from: http://eppi.ioe.ac.uk/cms/Projects/DepartmentofHealthandSocialCare/Publishedreviews/COVID-19Livingsystematicmapoftheevidence/tabid/3765/Default.aspx. (Access date: 07.10.2020).
32. The Health Foundation. COVID-19 policy tracker: The Health Foundation; 2020 [Available from: https://www.health.org.uk/news-and-comment/charts-and-infographics/covid-19-policy-tracker. (Access date: 08.09.2020).
33. The Centre for Informed Consent Integrity. Covid-19: Governance, ethics, evidence, policy practice: Global Foundation; 2020 [Available from: https://ge2p2global-centerforinformedconsentintegrity.org/category/covid-19/. (Access date: 12.10.2020).
34. NICE. Speciality guides: NICE; 2020 [Available from: https://www.nice.org.uk/covid-19/specialty-guides. (Access date: 02.10.2020).
35. UK Government. Freedom of Information Act. Sect. ss 19 and 20 (2000).
36. Information Commissioner's Office. Model publication scheme: Freedom of Information Act. 2015. https://ico.org.uk/media/for-organisations/documents/1153/model-publication-scheme.pdf. (Access date: 12.11.2020).
37. Ouzzani M, Hammady H, Fedorowicz Z, *et al.* Rayyan—a web and mobile app for systematic reviews. Systematic Reviews. 2016;5(1):210. 10.1186/s13643-016-0384-4.
38. Stevens S, Pritchard A. Third Phase of NHS Response to Covid-19. 2020. https://www.england.nhs.uk/coronavirus/wp-content/uploads/sites/52/2020/07/20200731-Phase-3-letter-final-1.pdf. (Access date: 15.09.2020).
39. National Voices. Five principles for the next phase of the Covid-19 response. 2020. https://www.nationalvoices.org.uk/sites/default/files/public/publications/5_principles_statement_091020.pdf. (Access date: 17.08.2020).
40. Strech D, Sofaer N. How to write a systematic review of reasons. Journal of Medical Ethics. 2012;38(2):121-6. 10.1136/medethics-2011-100096.
41. Brouwers M, Kho ME, Browman GP, *et al.* AGREE II: Advancing guideline development, reporting and evaluation in healthcare. Canadian Medical Association Journal. 2010;182(18):E839-E42. 10.1503/cmaj.090449.
42. Tricco AC, Garritty CM, Boulos L, *et al.* Rapid review methods more challenging during COVID-19: commentary with a focus on 8 knowledge synthesis steps. J Clin Epidemiol. 2020;126:177-83. 10.1016/j.jclinepi.2020.06.029.
43. The AGREE Next Steps Consortium. Appraisal of Guidelines for Research & Evaluation II: AGREE II Instrument. 2017. https://www.agreetrust.org/wp-content/uploads/2017/12/AGREE-II-Users-Manual-and-23-item-Instrument-2009-Update-2017.pdf. (Access date: 04.08.2020).
44. Academy of Medical Royal Colleges. Principles for reintroducing health services. COVID-19. 2020. https://www.aomrc.org.uk/wp-content/uploads/2020/05/COVID-19_Principles_for_reintroducing_healthcare_services_0520.pdf (Access date: 24.09.2020).
45. Academy of Medical Royal Colleges. Reset, restore and recovery: staff support. 2020. https://www.aomrc.org.uk/wp-content/uploads/2020/06/200622_RRR_-Staff_support.pdf (Access date: 24.09.2020).
46. Academy of Medical Royal Colleges. COVID-19. Effects on health from non-COVID-19 conditions and moving forward to deliver healthcare for all. 2020. https://www.aomrc.org.uk/wp-

For peer review only - http://bmjopen.bmj.com/site/about/guidelines.xhtml

content/uploads/2020/05/200515_COVID-19_moving_forward_to_deliver_healthcare.pdf. (Access date: 24.09.2020).
47. Academy of Medical Royal Colleges. Preparing for COVID-19 surges and winter. 2020. https://www.aomrc.org.uk/wp-content/uploads/2020/07/200717_Preparing_for_covid-19_surges_winter.pdf. (Access date: 24.09.2020).
48. Academy of Medical Royal Colleges. Health protection: public and professional responsibilities. 2020. https://www.aomrc.org.uk/wp-content/uploads/2020/06/200611_Health_protection_public_professional_responsibilities.pdf (Access date:
49. Academy of Medical Royal Colleges. Reset, restore and recovery: medical education and training. 2020. https://www.aomrc.org.uk/wp-content/uploads/2020/06/200622_RRR_Medical_Education_Training.pdf. (Access date: 24.09.2020).
50. Academy of Medical Royal Colleges. Reset, restore and recovery: equalities. 2020. https://www.aomrc.org.uk/wp-content/uploads/2020/06/200622_RRR_Equalities.pdf. (Access date: 24.09.2020).
51. Palmer C, Johnson P. Second phase of NHS response to COVID-19 for cancer services. 2020. London, UK. https://www.england.nhs.uk/coronavirus/wp-content/uploads/sites/52/2020/06/C0511-second-phase-of-nhs-response-to-covid-19-for-cancer-services-letter.pdf. (Access date:
52. National Health Service. Operating framework for urgent and planned services in hospital settings during COVID-19. 2020. https://covidlawlab.org/wp-content/uploads/2020/06/Operating-framework-for-urgent-and-planned-services-within-hospitals.pdf. (Access date: 29.09.2020).
53. National Health Service. WRES briefing for board and COVID-19 emergency preparedness, resilience and response (EPRR) membership in the NHS. 2020. https://www.england.nhs.uk/coronavirus/publication/wres-briefing-for-board-and-covid-19-emergency-preparedness-resilience-and-response-eprr-membership-in-the-nhs/. (Access date: 29.09.2020).
54. Public Health England. COVID-19: Guidance for the remobilisation of services within health and care settings, infection prevention and control recommendations 2020. https://assets.publishing.service.gov.uk/government/uploads/system/uploads/attachment_data/file/910885/COVID-19_Infection_prevention_and_control_guidance_FINAL_PDF_20082020.pdf. (Access date:
55. National Institute for Health and Care Excellence. Delivering paediatric elective surgery service during the COVID-19 pandemic. 2020. [18.09.2020]. Available from: https://www.nice.org.uk/sharedlearning/delivering-a-paediatric-elective-surgery-service-during-the-covid-19-pandemic. (Access date: 18.09.2020).
56. Jay N. COVID-19 - guidance for planning paediatric staffing and rotas. 2020. https://www.rcpch.ac.uk/resources/covid-19-guidance-planning-paediatric-staffing-rotas. (Access date: 29.09.2020).
57. Children and Young People Engagement Team. COVID-19 & Us - views from RCPCH & Us. 2020. https://www.rcpch.ac.uk/resources/covid-19-us-views-rcpch-us. (Access date: 25.09.2020).
58. Wilkinson D, Linney, M. Ethics framework for use in acute paediatric settings during COVID-19 pandemic. 2020. https://www.rcpch.ac.uk/resources/ethics-framework-use-acute-paediatric-settings-during-covid-19-pandemic. (Access date:
59. Royal College of Paediatrics and Child Health. National guidance for the recovery of elective surgery in children. RCPCH; 2020. https://www.rcpch.ac.uk/resources/national-guidance-recovery-elective-surgery-children#summary-of-recommendations. (Access date: 25.09.2020).
60. Royal College of Paediatrics and Child Health. Reset, Restore, Recover - RCPCH principles for recovery. 2020. https://www.rcpch.ac.uk/resources/reset-restore-recover-rcpch-principles-recovery. (Access date: 25.09.2020).

For peer review only - http://bmjopen.bmj.com/site/about/guidelines.xhtml

61. Fabbrani E, ., Mahajan R, . It is right to restart services, but we must do so in a safe way. Royal College of Anaesthetists2020. [30.09.2020]. Available from: https://www.rcoa.ac.uk/blog/it-right-restart-services-we-must-do-so-safe-way. (Access date: 30.09.2020).
62. Royal College of Midwives. Antenatal Care for women without suspected or confirmed COVID-19 and living in a symptom free household. 2020. https://www.rcm.org.uk/media/4180/clinical-guidance-briefing-one-antenatal-care-for-women-without-symptomsfinalv4-1.pdf. (Access date: 29.09.2020).
63. Royal College of Midwives. RCM Briefing on Re-introduction of visitors to Maternity Units across the UK during the COVID-19 pandemic. 2020. https://www.rcm.org.uk/media/4161/rcm-briefing-on-reintroduction-of-visitors-to-maternity-units-in-the-covid-pandemic-003.pdf. (Access date: 29.09.2020).
64. Royal College of Midwives. RCM Clinical Briefing Sheet: ‘freebirth’ or ‘unassisted childbirth’ during the COVID-19 pandemic. 2020. https://www.rcm.org.uk/media/3923/freebirth_draft_30-april-v2.pdf. (Access date: 30.09.2020).
65. Brigante L, Jokinen, M., Ross-Davie, M., Harlev-Lam, B., Morris, E., O’Brien, P., Jardine, J,. Relph, S., Powell, A. Guidance for provision of midwife-led settings and home birth in the evolving coronavirus (COVID-19) pandemic. 2020. https://www.rcm.org.uk/media/4099/2020-05-22-guidance-for-provision-of-midwife-led-settings-and-home-birth-in-the-evolving-coronavirus-covid-19-pandemic.pdf (Access date: 30.04.2021).
66. Royal College of Midwives. Equality essentials: Appropriate risk assessment during the current pandemic. 2020. https://www.rcm.org.uk/media/3939/risk-assessment-wraparound-guidance-a3-may-2020.pdf (Access date: 30.09.2020).
67. Royal College of Midwives. COVID-19 impact on Black, Asian and Minority ethnic (BAME) women. 2020. https://www.rcm.org.uk/media/4164/covid-19-impact-on-black-asian-and-minority-ethnic-bame-women.pdf. (Access date: 29.09.2020).
68. Royal College of Obstretricians and Gynaecologists. Principles for the testing and triage of women seeking maternity care in hospital settings during the COVID-19 pandemic: a supplimentary framework for maternity healthcare professionals. London, UK; 2020. https://www.rcog.org.uk/globalassets/documents/guidelines/2020-08-10-principles-for-the-testing-and-triage-of-women-seeking-maternity-care-in-hospital-settings-during-the-covid-19-pandemic.pdf. (Access date: 29.09.2020).
69. Ross-Davie M, Lambert, J., Brigante, L., Livingstone, C., Crowe, S., Pandya, P., Morris, E., O'Brien, P., Jardine, J., Relph, S., Goodyear, G. Guidance for antenatal and postnatal services in the evolving coronavirus (COVID-19) pandemic. 2020. https://www.rcm.org.uk/media/4132/2020-06-18-guidance-for-antenatal-and-postnatal-services-in-the-evolving-coronavirus-covid-19-pandemic.pdf. (Access date: 30.09.2020).
70. Royal College of Midwives. Antenatal care for women with current suspected or confirmed COVID-19 or with a member of their household with suspected or confirmed COVID-19. 2020. https://www.rcm.org.uk/media/4172/professional-briefingpaper-two-antenatal-care-for-women-with-suspected-or-confirmed-covid19-v31-240720.pdf. (Access date: 29.09.2020).
71. Royal College of Midwives. Domestic Abuse: identifying, caring for and supporting women at risk of/victims of domestic abuse during Covid-19. 2020. https://www.rcm.org.uk/media/4438/domestic-abuse-covid-short-guidance-on-template-final-v22.pdf. (Access date: 29.09.2020).
72. Royal College of Midwives. Bereavement Care in Maternity Services During COVID-19 pandemic. 2020. https://www.rcm.org.uk/media/4162/maternity-bereavement-care-during-covid-19-v5-150720.pdf. (Access date: 30.09.2020).
73. Royal college of Midwives. Postnatal Care for women with suspected or confirmed COVID-19 2020. https://www.rcm.org.uk/media/4207/briefing-no-9-postnatal_clinical_advice_with-covid_140820.pdf. (Access date: 30.09.2020).

For peer review only

For peer review only - http://bmjopen.bmj.com/site/about/guidelines.xhtml

74. Royal College of Midwives. Virtual Consultations. 2020. https://www.rcm.org.uk/media/4192/virtual-consultations-v20-24-july-2020-review-24-august-2020-1.pdf. (Access date: 30.09.2020).

75. Royal College of Anaethetists. Restarting planned surgery in the context of the COVID-19 pandemic. 2020. https://static1.squarespace.com/static/5e6613a1dc75b87df82b78e1/t/5eac2a173d65cd27933fca88/1588341272367/Restarting-Planned-Surgery.pdf. (Access date: 28.09.2020).

76. National Health Service. Delivering midwifery intrapartum care where local COVID-19 escalation protocols are required to be enacted. 2020. Report No.: 001559. https://www.nice.org.uk/Media/Default/About/COVID-19/Specialty-guides/Delivering-midwifery-intrapartum-care-where-local-COVID-19-escalation-protocols-are-required-to-be-enact.pdf. (Access date: 30.09.2020).

77. National Health Service. Supporting pregnant women using maternity services during the coronavirus pandemic: actions for NHS providers. 2020. UK. https://www.england.nhs.uk/coronavirus/wp-content/uploads/sites/52/2020/12/C0961-Supporting-pregnant-women-using-maternity-services-during-the-coronavirus-pandemic-actions-for-NHS-provi.pdf. (Access date: 23.03.2020).

78. Pritchard A, Kelly J. Important - for action - Operational priorities for winter and 2021/22. 2020. UK. https://www.england.nhs.uk/coronavirus/wp-content/uploads/sites/52/2020/12/important-for-action-operational-priorities-winter-and-2021-22-sent-23-december-2020.pdf. (Access date: 16.03.2020).

79. Academy of Medical Royal Colleges. National Clinical Prioritsation Programme (including evidence based interventions): Frequently asked questions. 2020. https://www.aomrc.org.uk/wp-content/uploads/2020/11/National_Clinical_Validation_Programme_FAQ_1120.pdf. (Access date: 18.03.2020).

80. Royal College of Paediatrics and Child Health. Digital by default or digital divide? Virtual healthcare consultations with young people 10-25 years. 2020. (Access date: 18.03.2020).

81. Royal College of Paediatrics and Child Health. Restoring children's health services: COVID-19 and winter planning - position statement. 2020. https://www.rcpch.ac.uk/sites/default/files/generated-pdf/document/Restoring-children%25E2%2580%2599s-health-services%252C-COVID-19-and-winter-planning---position-statement-.pdf. (Access date: 16.03.2020).

82. Royal College of Midwives. Coronavirus (COVID-19) infection in pregancy. 2020. https://www.rcm.org.uk/media/4390/2020-10-14-coronavirus-covid-19-infection-in-pregnancy-v12.pdf. (Access date: 18.03.2020).

83. Royal College of Obstretricians and Gynaecologists, Royal College of Midwives. Joint RCOG & RCM statement: planning for winter 2020/21 - reducing the impact of COVID-19 on maternity services in the UK. 2020. https://www.rcog.org.uk/globalassets/documents/guidelines/2020-10-08-rcog_rcm_winter_secondwave_statement.pdf. (Access date: 18.03.2020).

84. Royal College of Midwives. Midwives call for common sense of maternity visiting guidance [press release]. 2020. https://www.rcm.org.uk/media-releases/2020/december/midwives-call-for-common-sense-on-maternity-visiting-guidance/. (Access date: 18.03.2020).

85. Kasaven LS, Saso S, Barcroft J*, et al.* Implications for the future of Obstetrics and Gynaecology following the COVID-19 pandemic: a commentary. BJOG : an international journal of obstetrics and gynaecology. 2020;127(11):1318-23. 10.1111/1471-0528.16431.

86. Renfrew MJ, Cheyne H, Craig J*, et al.* Sustaining quality midwifery care in a pandemic and beyond. Midwifery. 2020;88:102759. 10.1016/j.midw.2020.102759.

87. Sokol D, Dattani R. How should surgeons obtain consent during the covid-19 pandemic? BMJ. 2020;369:m2539. 10.1136/bmj.m2539.

88. Chervenak FA, McCullough LB, Grünebaum A*, et al.* Professionally responsible advocacy for women and children first during the COVID-19 pandemic: guidance from World Association of

For peer review only - http://bmjopen.bmj.com/site/about/guidelines.xhtml

Perinatal Medicine and International Academy of Perinatal Medicine. Journal of perinatal medicine. 2020;48(9):867-73. doi:10.1515/jpm-2020-0329.
89. Reingold RB, Barbosa I, Mishori R. Respectful maternity care in the context of COVID-19: A human rights perspective. International journal of gynaecology and obstetrics: the official organ of the International Federation of Gynaecology and Obstetrics. 2020. 10.1002/ijgo.13376.
90. National Institute for Health and Care Excellence. COVID-19 rapid guideline: arranging planned care in hospitals and diagnostic services 2020. Report No.: NG179. https://www.nice.org.uk/guidance/ng179/resources/covid19-rapid-guideline-arranging-planned-care-in-hospitals-and-diagnostic-services-pdf-66141969613765. (Access date: 18.09.2020).
91. Huxtable R. Bin it or pin it? Which professional ethical guidance on managing COVID-19 should I follow? BMC Medical Ethics. 2020;21(1):60. 10.1186/s12910-020-00491-5.
92. NHS Confederation. A road map to reset our shared future. 2020. https://www.nhsconfed.org/-/media/Confederation/Files/Publications/Documents/A-road-map-to-reset-our-shared-future.pdf. (Access date: 25.11.2020).
93. Viens AM, McGowan CR, Vass VM. Moral distress among healthcare workers: ethics support is a critical part of the puzzle. BMJ Blogs2020. [cited 22nd September 2020 16.11.2020]. Available from: https://blogs.bmj.com/bmj/2020/06/23/moral-distress-among-healthcare-workers-ethics-support-is-a-crucial-part-of-the-puzzle/. (Access date: 16.11.2020).
94. Fritz Z, Huxtable R, Ives J, *et al.* Ethical road map through the covid-19 pandemic. BMJ. 2020;369:m2033. 10.1136/bmj.m2033.
95. UK Government. Our plan to rebuild: The UK Government's COVID-19 recovery strategy. 2020. London, UK. https://www.gov.uk/government/publications/our-plan-to-rebuild-the-uk-governments-covid-19-recovery-strategy/our-plan-to-rebuild-the-uk-governments-covid-19-recovery-strategy#our-approach-a-phased-recovery. (Access date: 03.11.2020).
96. Department of Health and Social Care. Meeting note. 2020. London. UK Government. https://app.box.com/s/jnwfj507tzuho5fjx181hwgmeh868vgh/file/677938102935. (Access date: 12.01.2021).
97. Huxtable R. COVID-19: where is the national ethical guidance? BMC Medical Ethics. 2020;21(1):32. 10.1186/s12910-020-00478-2.
98. Scottish Government. COVID-19 guidance: ethical advice and support framework. 2020. 1-21. https://www.gov.scot/publications/coronavirus-covid-19-ethical-advice-and-support-framework/. (Access date: 12.01.2021).
99. Together for Devon. Devon Ethics Paper for Organisational Boards. Devon; 2020. https://www.togetherfordevon.uk/download/devon-ethics-paper-for-organisational-boards/. (Access date: 12.01.2021).
100. NHS England & NHS Improvement. Good practice for working with people and communities during the COVID-19 outbreak. 2020. https://www.england.nhs.uk/participation/news/. (Access date: 25.11.2020).
101. Bok S. Lying: moral choice in public and private life. New York: Pantheon.; 1978.
102. Kerasidou A. Trust me, I'm a researcher!: The role of trust in biomedical research. Medicine, Health Care & Philosophy. 2017;20(1):43-50. 10.1007/s11019-016-9721-6.
103. O'Neill O. Autonomy and trust in bioethics. Cambridge: Cambridge University Press [electronic book]; 2002. Available from: http://ebookcentral.proquest.com/lib/liverpool/detail.action?docID=202188.
104. UK Government. The NHS Constitution: the NHS belongs to us all. 2015. London, UK. https://assets.publishing.service.gov.uk/government/uploads/system/uploads/attachment_data/file/480482/NHS_Constitution_WEB.pdf. (Access date: 16.11.2020).
105. Norheim OF, Abi-Rached JM, Bright LK, *et al.* Difficult trade-offs in response to COVID-19: the case for open and inclusive decision making. Nature Medicine. 2021;27(1):10-3. 10.1038/s41591-020-01204-6.

For peer review only - http://bmjopen.bmj.com/site/about/guidelines.xhtml

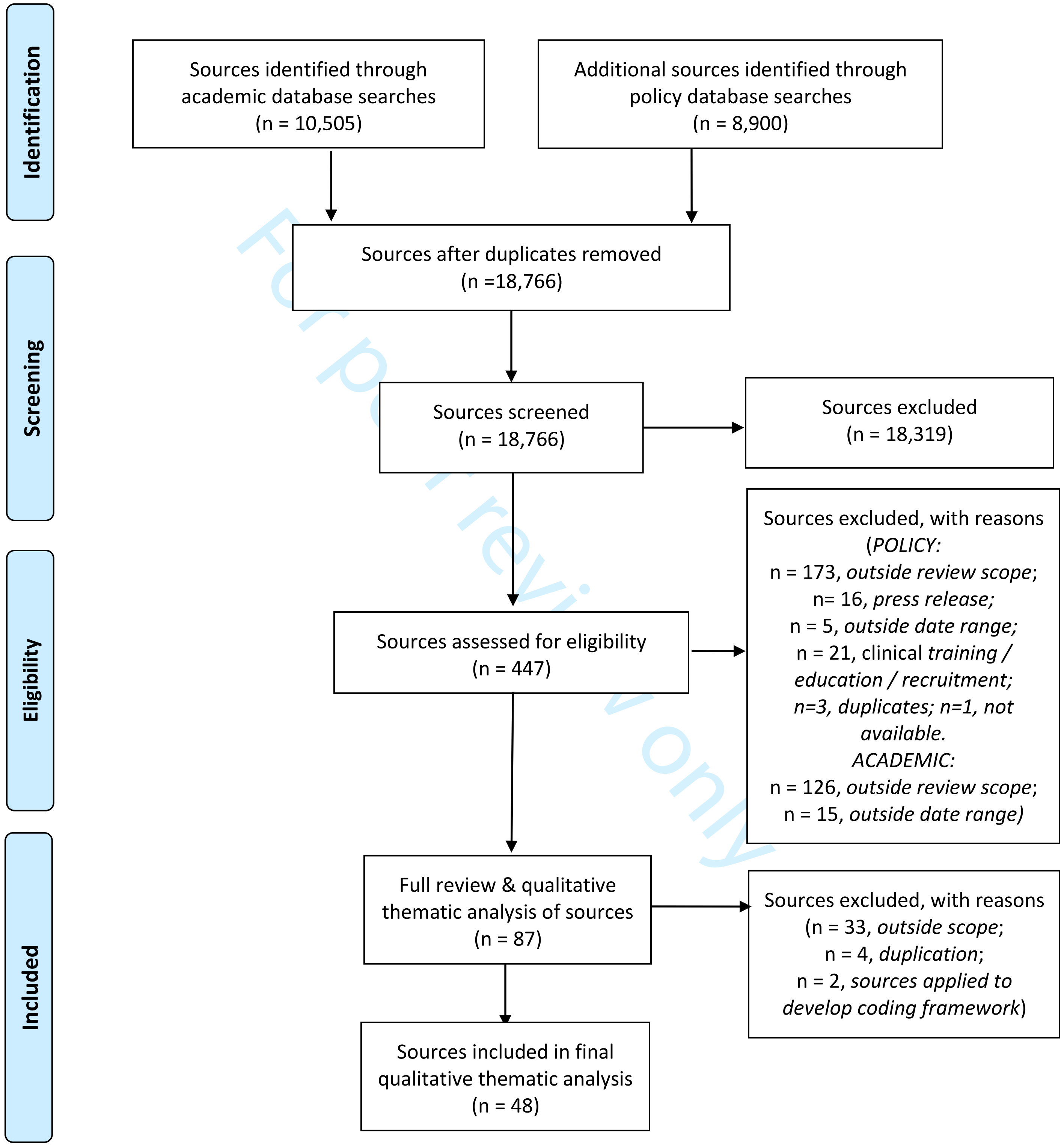

From: Moher D, Liberati A, Tetzlaff J, Altman DG, The PRISMA Group (2009). *P*referred *R*eporting *I*tems for *S*ystematic Reviews and *M*eta-*A*nalyses: The PRISMA Statement. PLoS Med 6(7): e1000097. doi:10.1371/journal.pmed1000097

For more information, visit www.prisma-statement.org.

For peer review only - http://bmjopen.bmj.com/site/about/guidelines.xhtml

Which ethical values underpin England's National Health Service reset of paediatric and maternity services following Covid-19: a rapid review.

SUPPLIMENTARY FILES

FILE 1: RAPID REVIEW PROTOCOL

Background and review rationale:

The response to Covid 19 (C19) will have far-reaching consequences for the NHS. The *Everyday and pandemic ethics* project will explore how the ethical issues created by this response have been approached by providers of non-C19 services. Notably we will explore how decisions on service prioritisation and reconfiguration have been made in the "reset" phase that has followed the first acute phase of the C19 pandemic. We define this "reset" phase as commencing from April 29th 2020, as NHS services were instructed on that date to prepare to recommence the delivery of non-covid surgical services (https://www.england.nhs.uk/coronavirus/wp-content/uploads/sites/52/2020/04/second-phase-of-nhs-response-to-covid-19-letter-to-chief-execs-29-april-2020.pdf). The "resetting" of NHS services encompasses the following:

- The resumption of service delivery incorporating revised procedures and practices to control the spread of C19 (e.g. the wearing of face coverings);
- Preparation for, and management of, second "waves" or recurrent spikes of C19, at both the national and local levels;
- The opportunities to reconfigure health services, for example accelerating the use of tele-medicine.

The focus on the reset phase emphasises the unique factors affecting ethical decision-making as services are re-established following the acute phase of the C19 pandemic.

We will focus on ethical decision-making in two non-C19 areas: maternity and paediatrics. We have chosen these areas because they have been significantly affected by the C19 response due to resource allocation away from these areas, with professional and patient organisations highlighting problematic effects on both areas (Association of Paediatric Anaestetists of Great Britain and Ireland, 2020; First 1001 Days Movement, 2020; McDonald et al., 2020). Specifically, the review will focus on "maternity services" (pre-natal, intrapartum, and post-partum care); and the resumption of paediatric surgery (encompassing critical / intensive care admissions, surgery, hospital discharge, and aftercare, referred to as "paediatric critical care and surgery services") during the C19 reset phase.

The objective of this review is to provide an initial understanding of the ethical values explicitly or implicitly engaged to inform decision-making about maternity services, and the resumption of paediatric critical care and surgery during the reset phases following the C19 pandemic in England. We adopt a pragmatic approach in order to make the best available use of existing evidence relating to this topic. The evidence will include diverse sources such as Government and Hospital trust policies, statements and decision support tools; reports and statements from professional bodies and charitable organisations; and evidence reviews and commentaries in academic journals. The approach aims to be broad and inclusive by combining searches of bibliographic databases with grey literature, hand searching, snowballing references of included sources, and engaging key topic stakeholders in an effort to verify completeness of sources. These approaches aim to ensure flexibility in identifying relevant sources both systematically and in the most efficient and pragmatic manner.

We will report key characteristics of all sources, and will appraise sources against a coding framework adapted from the Ethical Framework embedded in the Government's Pandemic Flu policy (UK Government, 2017). This framework is intended to guide all UK NHS decision-making during the rapid

For peer review only

readjustment of services due to a pandemic. Recognising that the reset phase requires different decision-making to the acute phase, we have adapted the framework by drawing upon two interlinked national documents (a letter on “Third phase of NHS response to Covid”, 31st July 2020 (Stevens & Pritchard, 2020); and the National Voices “Five principles for the next phase of the Covid-19 response”, published June 2020 (National Voices, 2020)). These adaptations aim to reflect the particular ethical considerations relevant to the “reset” phase. We recognise that this adaptation creates a tension between the rapid review methodology and findings, which we discuss alongside the revised framework below. In our analysis we will draw upon the systematic review of reasons approach (Strech & Sofaer, 2012) to facilitate explicit consideration of ethical values being applied to inform decision-making in non-C19 maternity services, and paediatric critical care and surgery services during the C19 reset phases in England.

This rapid evidence review forms the first stage of a larger project, providing a snapshot of ethical decision-making in maternity and paediatric care to inform subsequent stages of the *Everyday and Pandemic Ethics* study. Review findings will be available as immediate recommendations for ethical best practice – for example by examining the transparency of written policies against standards in the 2016 Pandemic Flu Policy - for paediatric and maternity services delivery during the C19 reset phases.

Objective

The objective of this review is to answer the question: what ethical values guide decision-making in non-C19 paediatric critical care and surgery and maternity services during the C19 reset phases in England? Achieving this objective will entail exploring a range of decision-making factors, such how are involved in decision-making, what decisions have been made, and how decisions are justified, identifying implicit and explicit ethical values.

Methodology

To ensure a rigorous review methodology, we have drawn upon the ENTREQ guidelines for qualitative research synthesis (Tong, Flemming, McInnes, Oliver, & Craig, 2012) and the systematic review of reasons approach developed for normative review questions (Strech & Sofaer, 2012). Integrating these approaches address the critique that literature reviews exploring normative considerations often fail to clearly report the methodological approach taken (Mertz, Strech, & Kahrass, 2017).

Inclusion and exclusion criteria

Inclusion criteria

This review will consider sources developed to guide non-C19 paediatric critical care and surgery services and maternity services during the reset phases of C19; or that discuss the application of ethical values to paediatric critical care and surgery services and maternity services during the reset phases of C19.

The review will include sources relating to England, including national policies (that include England), and policies from Trusts and individual hospitals across England, including our case study sites (in North West England and the Midlands). We will be restricted to sources written in the English language, and published after 29th April 2020.

Exclusion criteria

Sources published prior 29th April 2020, that discuss healthcare delivery broadly; or that discuss maternity or paediatric critical care or surgery services during the acute phase of the C19 pandemic in England (defined as the start of lockdown on 23rd March until the 29th April 2020) will be excluded.

Data sources

The review will include the following data sources:

For peer review only

- National policies guiding the implementation of non-C19 maternity services, and/or paediatric critical care and surgery services; and/or providing an ethical framework or decision-making tools for healthcare reorganisation of these services during the C19 reset phases;
- Local trust and hospital policies guiding the implementation of non-C19 maternity and paediatric critical care and surgery services; and/or providing an ethical framework or decision-making tools for healthcare reorganisation in these services during the C19 reset phases;
- Guidelines and statements from Royal Medical Colleges relating to the implementation of non-C19 maternity and paediatric critical care and surgery services and/or providing an ethical framework or decision-making tools for healthcare reorganisation in these services during the C19 reset phases;
- Working papers and committee reports discussing the re-orientation of non-C19 maternity and paediatric critical care and surgery services during the C19 reset phases;
- Evidence reviews and primary qualitative and quantitative research on the re-orientation of non-C19 maternity and paediatric critical care and surgery services during the C19 reset phases;
- Peer-reviewed commentaries and grey-literature discussing experiences of non-C19 maternity, and paediatric critical care and surgery services during the C19 reset phases.

All sources will be obtained from online platforms, or via e-mail for Freedom of Information requests and stakeholder contributions.

Electronic search strategy

We will conduct searches in September 2020, with an additional search prior to the publication of the review to check for sources published in the interim. We will search the following academic bibliographic databases: PubMed and PubMeds Covid-19 database LitCOVID (https://www.ncbi.nlm.nih.gov/research/coronavirus/). We will also search clearing houses of C19 related research including the EPPI Centre living map of Covid-19 evidence (http://eppi.ioe.ac.uk/cms/Projects/DepartmentofHealthandSocialCare/Publishedreviews/COVID-19Livingsystematicmapoftheevidence/tabid/3765/Default.aspx), COVID END (https://www.mcmasterforum.org/networks/covid-end), evidence aid (https://evidenceaid.org/evidence/coronavirus-covid-19/ - which includes reviews being conducted by the Campbell Collaboration), and the Cochrane Collaboration.

For academic bibliographic databases we will search using the following terms:

1. (Covid OR Covid-19 OR coronavirus* OR SARS-CoV-2 OR Severe Acute Respiratory Syndrome OR pandemic) AND
2. (Matern*) OR (pre-natal OR inter-partum OR post-natal OR perinatal) OR (labour OR pregnan*) OR (obstetrics) OR (birth*) OR (Midwife*) AND
3. (paediatric OR pediatric) AND (critical OR intensive OR acute) OR (operati* OR theatre*) OR (child*) OR (surg*) AND
4. (doctor) OR (nurs*) AND
5. (service*) OR (design OR deliver*) OR (allocat* OR priorit*) OR (care) OR (policy OR guideline*)

Searchers will be conducted step-wise, first conducting searches relating to Maternity service combining rows 1,2, 4 and 5 above; and secondly for Paediatric critical care and surgery, combining rows 1,3, 4 and 5 above.

To complement academic databases, and recognising the scope of the research question, we will also search grey literature sources including the websites of NHS Trusts (including our case study sites),

For peer review only - http://bmjopen.bmj.com/site/about/guidelines.xhtml

the UK Government (gov.uk), and websites of professional bodies (e.g. Academy of Royal Colleagues and the Royal College of Paediatrics / Midwifery and NICE). We will also search clearinghouses of C19 related grey literature such as policy documents, for example the Health Foundation C19 Policy Tracker (https://www.health.org.uk/news-and-comment/charts-and-infographics/covid-19-policy-tracker).

Study screening methods

We will review all identified sources and any duplicates removed. Two members of the research team (AC, PB, CR, SF and LF) will double screen all identified results. Screening will be based on title and abstract / summary (where available). Where these are not available or no definitive decision can be made about whether a source meets the review inclusion criteria based on title and abstract/summary screening, additional full text review will be undertaken. To operationalise the inclusion criteria we applied the following scoring system:

0. Not included
1. Included: Identifies the approach taken to decision making (e.g. discusses a decision-making tool or framework)
2. Included: Identifies what decision has been made
3. Included: Identifies a justification for the decision taken

Where a source meets more than one of the inclusion criteria, all will be identified. Disagreements in double screening will be resolved through discussion with a third member of the review team (HD) not involved in initial screening to reach a consensus decision about inclusion or exclusion.

We will document all searches and screening assessments in a flow chart, with an accompanying narrative explanation, including explicit reasons for study exclusion.

Using the Freedom of Information Act 2000

The Freedom of Information Act 2000 (FOI) imposes two main duties on public authorities: one to proactively provide information, and the other to respond to requests for information. A model 'publication scheme' has been produced which public authorities are obliged to follow in making relevant information available. The model publication scheme sets out various classes of information, which are tailored to different authorities by a 'definition document' for each type of organisation. The classes of information are as follows:

- Who we are and what we do
- What we spend and how we spend it
- What our priorities are and how we are doing
- How we make decisions
- Our policies and procedures
- Lists and registers
- The services we offer

To aid access to NHS Trust information we will review Trusts' Freedom of Information Act Publication schemes and submit freedom of information (FOI) requests. Our publication scheme reviews and FOI requests will target our case study sites, as well as additional NHS Trusts with Clinical Ethics Committees as listed on the UKs Clinical Ethics Network. Both the reviews and the FOI requests will explicitly focus on sources (e.g. meeting minutes, policies, or decision-making tools) guiding maternity services and paediatric critical care and surgery services developed for the reset period. FOI requests will be submitted to individual hospitals and NHS Trusts, as well as at regional and national decision-making levels. To mirror database searches, we will repeat the publication scheme reviews and the FOI requests prior to publication of the review for the inclusion of additional sources.

For peer review only

After the initial searches, publication scheme reviews and results from FOI requests, we will share results with Trust and project stakeholders to conduct a completeness check and request additional missing sources be identified for screening and potential inclusion. We will furthermore search citations of included sources for snowball sampling.

Appraisal of sources

Given the reviews focus on normative values, we will apply the PROGRESS Plus tool[1] to identify the extent to which sources consider characteristics recognised to affect health equity (https://methods.cochrane.org/equity/projects/evidence-equity/progress-plus). This tool covers factors including place of residence, race/ethnicity/culture/language, occupation, gender/sex, religion, education, socioeconomic status, and social capital (O'Neill et al., 2014); as well as "plus" factors such as age and disability, relational features (such as single parent household), and time-dependent relationships (e.g. receiving in-patient care). Assessing sources against these will identify the extent to which sources are systematically considering various aspects of health equity.

In addition, for peer reviewed literature we will apply the relevant CASP checklist[2] (https://casp-uk.net/casp-tools-checklists/), and for policy sources the AGREE-II tool developed for assessing healthcare practice guidelines (Brouwers et al., 2010).

Data extraction and management

We will report the following characteristics of included sources:

- Publication type (e.g. policy, report, professional body guideline, peer reviewed article, commentary piece, decision-support tool, etc);
- Month and year of publication;
- Population (maternity or paediatric services);
- Source scope (national, regional, trust, hospital, etc);
- Where relevant for primary research we will also report: the primary research question, methodology, number of participants, and analysis approach.

Sources will be analysed against a coding framework. This coding framework has been developed by modifying the Ethical Framework embedded in the Government's Pandemic Flu policy (UK Government, 2017). The Ethical Framework in the Pandemic Flu Policy is guided by the fundamental principle of equal concern and respect, accompanied by 8 embedded principles designed to be applied as a checklist to help ensure that the full-range of ethical issues are considered in decision-making processes. It is the only framework explicitly intended to guide all UK NHS decision-making during the rapid readjustment of services due to a pandemic. However, recognising that the reset phase requires a different decision-making to the acute phase, we adapted the framework by drawing upon two interlinked national documents: (1) a letter from the NHS Chief Executive and Chief Operating Officer on "Third phase of NHS response to Covid", dated 31st July 2020 (Stevens & Pritchard, 2020), and (2) the National Voices "Five principles for the next phase of the Covid-19 response" published in June 2020 (National Voices, 2020). Our coding framework retains the Pandemic Flu 8 embedded principles, but adjusts their specification according to how they are operationalised in these two documents. We recognise this adaptation creates a methodological tension in our review as our coding framework is based upon a Framework adapted according to ethical documents relevant to the review scope and purpose. We believe this approach is justifiable given the lack of an overarching framework tailored

[1] This aspect of the review was not conducted due to time constraints.
[2] No peer reviewed studies reporting original data were included in the review, therefore this tool was not applied.

to the reset phase, and the need for a coding framework for the review that reflects the ethical specificities of this phase.

Extracting information from sources in relation to each of these adapted principles will identify whether the source engages with the normative values identified as important when making decisions during the C19 reset phase. The principles (retained from the national pandemic flu policy) and adapted sub-domains are as follows:

Ethical principle (from Pandemic Flu Ethical Framework)	Adapted sub-domain (based on NHS letter and National Voices Five Principles)
**Respect**	Involvement (i.e. right to express views on matters affecting them, engaging those affected by decisions)
	Respecting choices about personalised care (best interests of person as a whole)
	Collaborative working / engagement (organisational coordination; NHS volunteer scheme, clinical teams, CCGs, local authorities; co-production with voluntary sector, patient orgs etc)
**Recognising harms & balancing against benefits (physical, psychological, social & economic) - proportionality**	Recover operation of healthcare (inc. addressing backlog of care needs, resuming home visits for vulnerable / shielding where appropriate)
	Safety of NHS staff (physical, psychological, systemic inequalities, flexible working)
	Embrace new ways of working (e.g. telemedicine, home visits etc)
	Enhance crisis responsiveness (second wave)
	Accelerate preventative programmes (obesity reduction, seasonal flu, outreach to marginalised groups)
	Responsiveness (adapt plans to new circumstances / information)
**Reciprocity**	Concept of mutual exchange: take responsibility for own behaviour, reduce others expose others to risks
	Protect those at risk of C19 (physically, socially, BAME etc)
**Fairness**	Inclusivity in service recovery (e.g. barriers or access needs, support those with unequal access to care)
	Patient prioritisation (to address backlog i.e. clinical urgent / longest waiting etc)
	Reduce health inequalities (social inequalities & social determinants of health)
	Everyone matters equally & weighted equally in policies & any disproportionate impact on one particular group is accounted for
**Accountability**	Transparency (i.e. document decisions, clarity of who is responsible for decisions, governance arrangements, assess against milestones, sharing information to help others)

Recognising that the reset phase may incorporate responding to second waves of C19 infections, for example through localised lockdowns (as provided for in the UK Governments Covid-19 Contain framework: https://www.gov.uk/government/publications/containing-and-managing-local-coronavirus-covid-19-outbreaks/covid-19-contain-framework-a-guide-for-local-decision-makers), the principles and sub-domains within this assessment framework may be inductively revised on the basis of the sources reviewed. We will report any development of the framework as an outcome of the rapid review.

For peer review only

We will apply a scoring system to assess the inclusion and application of each principle domain. This will entail a 2-stage process, first answering “yes/no” to its inclusion and, secondly, rating application of each domain on a scale of 1-3, where:

1. ethical principle(s) inferred or mentioned but not clearly applied;
2. ethical principle(s) identified and its application described; and
3. ethical principle(s) application is discussed in-depth, including balancing against other principles.

Data synthesis

To further explore the data, we will conduct further analysis of sources from our case study sites (North West England and the Midlands) to conduct a thematic synthesis (Thomas & Harden, 2008)[3]. This approach will draw upon the review of reasons where the data is explored to identify reasons for adopting particular normative positions, and the consistency of these reasons across sources and settings (maternity or paediatrics). This will help to surface the range of reasons informing decision-making processes, and experiences of these decisions by those affected.

Data synthesis will be led by AC and PB, with regular review and discussion with the wider research team to ensure rigor of the approach to analysis.

Reporting

We will report this rapid review as brief reports summarising the approach to paediatric critical care and surgery services, and maternity services, during the reset phase of the C19 pandemic. This will identify the ethical values informing paediatric critical care and surgery services, and maternity services, during the reset phase of the C19 pandemic, and highlighting case study examples that explore the reasons for adopting a particular normative position. The report will be disseminated in the form of a short brief, shared with our stakeholder group comprised of representatives of National bodies, case study Trusts and Hospitals, and other relevant parties. We will also disseminate the review findings via social media (e.g. Twitter) and our project website (https://www.liverpool.ac.uk/population-health-sciences/departments/health-services-research/key-projects/resetethics/).

We will also develop a rapid review publication reporting the full results. It will go into more depth than the brief report about the methodology, and will offer an in-depth description of the response to planning for the reset phase of maternity services and paediatric critical care and surgery services in England. We will explore examples of good practice – such as where specific sources have engaged with the full breadth of ethical considerations, or where there is transparency in descriptions of ethical engagement and decision-making processes. From this, we will make recommendations for addressing areas where the normative basis of adopting specific approaches to service planning and delivery are unclear.

REFERENCES

Association of Paediatric Anaestetists of Great Britain and Ireland, A. (2020). APAGBI position statement regarding the delivery and recovery of Children's surgery during the coronavirus pandemic [Press release]. Retrieved from https://www.apagbi.org.uk/sites/default/files/inline-files/APA%20statement%2015.05.20%20Final.pdf

Brouwers, M., Kho, M. E., Browman, G. P., Burgers, J. S., Cluzeau, F., Feder, G., . . . Consortium, T. A. N. S. (2010). AGREE II: Advancing guideline development, reporting and evaluation in

[3] This aspect of the review is ongoing and is based primarily upon the Publication Scheme review data. In our paper we report initial findings from this.

healthcare. *Canadian Medical Association Journal, 182*(18), E839-E842. doi:10.1503/cmaj.090449
First 1001 Days Movement. (2020). Our call on Government to keep babies safe [Press release]. Retrieved from https://parentinfantfoundation.org.uk/our-call-on-government-to-keep-babies-safe/
McDonald, H. I., Tessier, E., White, J. M., Woodruff, M., Knowles, C., Bates, C., . . . Edelstein, M. (2020). Early impact of the coronavirus disease (COVID-19) pandemic and physical distancing measures on routine childhood vaccinations in England, January to April 2020. *Eurosurveillance, 25*(19), 2000848. doi:https://doi.org/10.2807/1560-7917.ES.2020.25.19.2000848
Mertz, M., Strech, D., & Kahrass, H. (2017). What methods do reviews of normative ethics literature use for search, selection, analysis, and synthesis? In-depth results from a systematic review of reviews. *Systematic Reviews, 6*(1), 261. doi:10.1186/s13643-017-0661-x
National Voices. (2020). *Five principles for the next phase of the Covid-19 response*. Retrieved from https://www.nationalvoices.org.uk/sites/default/files/public/publications/5_principles_statement_091020.pdf
O'Neill, J., Tabish, H., Welch, V., Petticrew, M., Pottie, K., Clarke, M., . . . Tugwell, P. (2014). Applying an equity lens to interventions: using PROGRESS ensures consideration of socially stratifying factors to illuminate inequities in health. *Journal of Clinical Epidemiology, 67*(1), 56-64. doi:10.1016/j.jclinepi.2013.08.005
Stevens, S., & Pritchard, A. (2020). *Third Phase of NHS Response to Covid-19*. Retrieved from https://www.england.nhs.uk/coronavirus/wp-content/uploads/sites/52/2020/07/20200731-Phase-3-letter-final-1.pdf.
Strech, D., & Sofaer, N. (2012). How to write a systematic review of reasons. *Journal of Medical Ethics, 38*(2), 121-126. doi:10.1136/medethics-2011-100096
Thomas, J., & Harden, A. (2008). Methods for the thematic synthesis of qualitative research in systematic reviews. *BMC Medical Research Methodology, 8*, 1-10. doi:10.1186/1471-2288-8-45
Tong, A., Flemming, K., McInnes, E., Oliver, S., & Craig, J. (2012). Enhancing transparency in reporting the synthesis of qualitative research: ENTREQ. *BMC Medical Research Methodology, 12*(1), 181-181. doi:10.1186/1471-2288-12-181
UK Government. (2017). *Pandemic Flu*. London, UK: UK Government Retrieved from https://www.gov.uk/guidance/pandemic-flu.

For peer review only

FILE 2: PUBMED SEARCH STRATEGY

Columns 1 and 2 describe the conceptual structure of the search input into PubMed. Column 3 provides an indicative example of how PubMed translated the natural language terms for each query by generating MeSH terms and using the natural language for all fields in the PubMed record. In addition to the below, in PubMed the date filter of "last 1 year", and language filter "English" were applied.

	**Natural language search terms (with wildcard truncation where relevant)**	**Search query in PubMed**
1	Covid	"sars cov 2"[MeSH Terms] OR "sars cov 2"[All Fields] OR "covid"[All Fields] OR "covid 19"[MeSH Terms] OR "covid 19"[All Fields] OR ("covid 19"[All Fields] OR "covid 19"[MeSH Terms] OR "covid 19 vaccines"[All Fields] OR "covid 19 vaccines"[MeSH Terms] OR "covid 19 serotherapy"[All Fields] OR "covid 19 serotherapy"[Supplementary Concept] OR "covid 19 nucleic acid testing"[All Fields] OR "covid 19 nucleic acid testing"[MeSH Terms] OR "covid 19 serological testing"[All Fields] OR "covid 19 serological testing"[MeSH Terms] OR "covid 19 testing"[All Fields] OR "covid 19 testing"[MeSH Terms] OR "sars cov 2"[All Fields] OR "sars cov 2"[MeSH Terms] OR "severe acute respiratory syndrome coronavirus 2"[All Fields] OR "ncov"[All Fields] OR "2019 ncov"[All Fields] OR (("coronavirus"[MeSH Terms] OR "coronavirus"[All Fields] OR "cov"[All Fields]) AND 2019/11/01:3000/12/31[Date - Publication])) OR ("coronavirus"[MeSH Terms] OR "coronavirus"[All Fields] OR "coronaviruses"[All Fields]) OR ("sars cov 2"[MeSH Terms] OR "sars cov 2"[All Fields] OR "sars cov 2"[All Fields]) OR ("severe acute respiratory syndrome"[MeSH Terms] OR ("severe"[All Fields] AND "acute"[All Fields] AND "respiratory"[All Fields] AND "syndrome"[All Fields]) OR "severe acute respiratory syndrome"[All Fields]) OR ("pandemic s"[All Fields] OR "pandemically"[All Fields] OR "pandemicity"[All Fields] OR "pandemics"[MeSH Terms] OR "pandemics"[All Fields] OR "pandemic"[All Fields])
2	Covid-19	
3	coronavirus*	
4	SARS-CoV-2	
5	Severe Acute Respiratory Syndrome	
6	Pandemic	
7	or/1-7	
8	Matern*	"matern*"[All Fields] OR ("pre-natal"[All Fields] OR "inter-partum"[All Fields] OR "post-natal"[All Fields] OR ("perinatal"[All Fields] OR "perinatally"[All Fields] OR "perinatals"[All Fields])) OR ("labor s"[All Fields] OR "labored"[All Fields] OR "laborer"[All Fields] OR "laborer s"[All Fields] OR "laborers"[All Fields] OR "laboring"[All Fields] OR "labors"[All Fields] OR "labour"[All Fields] OR "work"[MeSH Terms] OR "work"[All Fields] OR "labor"[All Fields] OR "labor, obstetric"[MeSH Terms] OR ("labor"[All Fields] AND "obstetric"[All Fields]) OR "obstetric labor"[All Fields] OR "laboured"[All Fields] OR "labourer"[All Fields] OR "labourers"[All Fields] OR "labouring"[All Fields] OR "labours"[All Fields] OR "pregnan*"[All Fields]) OR ("obstetric"[All Fields] OR "obstetrically"[All Fields] OR "obstetrics"[MeSH Terms] OR "obstetrics"[All Fields] OR
9	pre-natal OR inter-partum OR post-natal OR perinatal	
10	labour OR pregnan*	
11	Obstetrics	
12	birth*	
13	Midwife*	
14	or/8-13	

For peer review only

		"obstetrical"[All Fields]) OR "birth*"[All Fields] OR "midwife*"[All Fields]
15	paediatric OR pediatric	"paediatrics"[All Fields] OR "pediatrics"[MeSH Terms] OR "pediatrics"[All Fields] OR "paediatric"[All Fields] OR "pediatric"[All Fields] OR "paediatrics"[All Fields] OR "pediatrics"[MeSH Terms] OR "pediatrics"[All Fields] OR "paediatric"[All Fields] OR "pediatric"[All Fields] OR "critical"[All Fields] OR "critically"[All Fields] OR "intensive"[All Fields] OR "intensives"[All Fields] OR "acute"[All Fields] OR "acutely"[All Fields] OR "acutes"[All Fields] OR "operati*"[All Fields] OR "theatre*"[All Fields] OR "child*"[All Fields] OR "surg*"[All Fields]
16	critical OR intensive OR acute	
17	operati* OR theatre*	
18	child*	
19	surg*	
20	or/15-19	
21	Doctor	"doctor s"[All Fields] OR "doctoral"[All Fields] OR "doctorally"[All Fields] OR "doctorate"[All Fields] OR "doctorates"[All Fields] OR "doctoring"[All Fields] OR "physicians"[MeSH Terms] OR "physicians"[All Fields] OR "doctor"[All Fields] OR "doctors"[All Fields] OR "nurs*"[All Fields]
22	nurs*	
23	or/21-22	
24	service*	"service*"[All Fields] OR "design"[All Fields] OR "design s"[All Fields] OR "designabilities"[All Fields] OR "designability"[All Fields] OR "designable"[All Fields] OR "designed"[All Fields] OR "designer"[All Fields] OR "designer s"[All Fields] OR "designers"[All Fields] OR "designing"[All Fields] OR "designs"[All Fields] OR "deliver*"[All Fields] OR "allocat*"[All Fields] OR "priorit*"[All Fields] OR "care"[All Fields] OR "policy"[MeSH Terms] OR "policy"[All Fields] OR "policies"[All Fields] OR "policy s"[All Fields] OR "guideline*"[All Fields]
25	design OR deliver*	
26	allocat* OR priorit*	
27	Care	
28	policy OR guideline*	
29	or/24-28	
30	7 and 14 and 23 and 29	
31	7 and 20 and 23 and 29	

For peer review only

FILE 3: PUBLICATION SCHEME SEARCH STRATEGY

The publication scheme search focused on case study hospital Trusts. The focus of the search was the '*How we make decisions'* and *'Our policies and procedures'* sections of the Trust's Publication Scheme. As with the review, sources listed in the publication scheme were excluded if either:

a. they were dated before April 29th, 2020; or
b. their focus and content was on a period prior to April 29th, 2020 (for example an annual report for a financial year to 31st March);

For sources included, a high-level review was then carried out to identify any references to policies or other documents of interest (for example supporting documents or reports prepared for board meetings). The high-level review of included documents was carried out by CR by searching sources for reference to the following terms:

- Covid, Covid-19, coronavirus, SARS-CoV-2, Severe Acute Respiratory Syndrome or pandemic; AND
- Service or care design or delivery, allocation or priority policy, guideline, guidance or framework; OR
  - For paediatric services: Paediatric/pediatric, child/children, critical care, intensive care, acute care, surgery, operation, operating theatre.
  - For maternity services: Maternity, pre-natal, inter-partum, post-natal, perinatal, labour, pregnancy, obstetrics, birth or midwife.

For any sources not accessible through the Trust's publication scheme, Freedom of Information requests were submitted.

For peer review only

FILE 4: THEMATIC ANALYSIS OF PUBLICATION SCHEME CASE STUDY

Publication scheme class	Type of document	Date	Title of document	Themes identified	Sub themes identified
How we make decisions	Board meeting: supporting paper	June 2020	Covid-19 Pandemic – Trust Infection Prevention & Control Response	Respect	collaborative and agile working, patient involvement - eg re-considering place of birth preferences in the context of pressure on emergency ambulance transfer
				Recognising harms and balancing against benefits (physical, psychological, social and economic) – proportionality	staff, patient and visitor safety; testing procedures, agile working, telemedicine, responsiveness - nb availability of abortion medicines at home (no context to this but refs statutory change)
				Reciprocity	Staff expected to take care of their own health
				Accountability	Clear presentation of decisions, rationale, longer term changes to SOP etc.
How we make decisions	Board meeting: supporting paper	June 2020	Update on Covid-19 related Equality Issues	Respect	Involvement - staff and patients to engage in commms around their care and any specific vulnerabilities identified; collaborative working with staff reps, patient groups etc
				Recognising harms etc.	Safety of staff, safety of patients (physical, social, mental wellbeing; specific disadvantages considered - eg non-english speakers; forward planning to mitigate against widening of inequalities
				Reciprocity	Mutual exchange, consideration of social, physical and BAME risk factors

				Fairness<br><br>Accountability	Reducing health inequalities, equality impact assessments (EIAs) on all decisions,<br><br>specific governance decisions, implementation detail (eg EIAs), sharing information and clarity of lines of responsibility.
How we make decisions	Board meeting: supporting paper	June 2020	Safeguarding Service Provisions during COVID: Practice-focused document setting out safeguarding practice during Covid - specific to maternity services	Respect<br><br>Recognising harms etc<br><br>Reciprocity<br>Fairness<br><br>Accountability	Organised around creating safe spaces for disclosures - eg routine question added during a scan when partner is not present;<br><br>changing ways of working to ensure awareness of abuse is highlighted in practice, focus on patient safety, collaborative working (other agencies - medical and legal),<br><br>Focus is reduction of patient risk<br><br>Everyone matters equally, reduction of social inequalities, disporportionate impact of Covid on this at risk group (NB impact of domestic abuse on staff is also noted<br><br>Built into reporting and governance procedures

Which ethical values underpin England's National Health Service reset of paediatric and maternity services following Covid-19: a rapid review.

ENTREQ Checklist (Tong et al, 2012).

Item	Guide & description	Reported on (section & page no.)
Aim	State the research question the synthesis addresses	p.2, introduction
Synthesis methodology	Identify the synthesis methodology or theoretical framework which underpins the synthesis, and describe the rationale for choice of methodology (e.g. meta-ethnography, thematic synthesis, critical interpretive synthesis, grounded theory synthesis, realist synthesis, meta-aggregation, meta-study, framework synthesis).	p.3, methodology
Approach to searching	Indicate whether the search was pre-planned (comprehensive search strategies to seek all available studies) or iterative (to seek all available concepts until theoretical saturation is achieved).	p.3-4, methodology and supplementary file 1, rapid review protocol
Inclusion criteria	Specify the inclusion/exclusion criteria (e.g. in terms of population, language, year limits, type of publication, study type).	p.3, inclusion and exclusion criteria
Data sources	Describe the information sources used (e.g. electronic databases (MEDLINE, EMBASE, CINAHL, psychINFO, Econlit), grey literature databases (digital thesis, policy reports), relevant organisational websites, experts, information specialists, generic web searches (Google Scholar), hand searching, reference lists) and when the searches were conducted; provide the rationale for using the data sources.	p. 3-4, electronic search strategy
Electronic Search strategy	Describe the literature search (e.g. provide electronic search strategies with population terms, clinical or health topic terms, experiential or social phenomena related terms, filters for qualitative research and search limits).	p.3-4, electronic search strategy
Study screening methods	Describe the process of study screening and sifting (e.g. title, abstract and full text review, number of independent reviewers who screened studies	p.4, screening
Study characteristics	Present the characteristics of the included studies (e.g. year of publication, country, population, number of participants, data collection, methodology, analysis, research questions)	p.6, results, Table 2: key characteristics of sources
Study selection results	Identify the number of studies screened and provide reasons for study exclusion (e.g.for comprehensive searching, provide numbers of studies screened and reasons for exclusion	p.6, results and Figure 1: PRISMA flow diagram

For peer review only

	indicated in a figure/flowchart; for iterative searching describe reasons for study exclusion and inclusion based on modifications t the research question and/or contribution to theory development).	
Rationale for appraisal	Describe the rationale and approach used to appraise the included studies or selected findings (e.g. assessment of conduct (validity and robustness), assessment of reporting (transparency), assessment of content and utility of the findings).	p. 4-5, data analysis
Appraisal items	State the tools, frameworks and criteria used to appraise the studies or selected findings (e.g. Existing tools: CASP, QARI, COREQ, Mays and Pope [25];reviewer developed tools; describe the domains assessed: research team, study design, data analysis and interpretations, reporting).	p. 6, data analysis
Appraisal process	Indicate whether the appraisal was conducted independently by more than one reviewer and if consensus was required.	p. 6, data analysis
Appraisal results	Present results of the quality assessment and indicate which articles, if any, were weighted/excluded based on the assessment and give the rationale.	P11, results, Table 3: Agree-II assessment of 42 policy guideline sources
Data extraction	Indicate which sections of the primary studies were analysed and how were the data extracted from the primary studies? (e.g. all text under the headings "results /conclusions" were extracted electronically and entered into a computer software).	p. 4-5, data analysis and Table 1: reset phase coding framework
Software	State the computer software used, if any.	p. 3, electronic search strategy identifies use of EndNote software; and p.4, screening identifies use of Rayyan software
Number of reviewers	Identify who was involved in coding and analysis	p. 2-3, electronic search strategy, screening, and data analysis identify authors involved in each stage
Coding	Describe the process for coding of data (e.g. line by line coding to search for concepts).	p.3, data analysis

For peer review only

Study comparison	Describe how were comparisons made within and across studies (e.g. subsequent studies were coded into pre-existing concepts, and new concepts were created when deemed necessary).	p.3, data analysis
Derivation of themes	Explain whether the process of deriving the themes or constructs was inductive or deductive.	p. 3, data analysis
Quotations	Provide quotations from the primary studies to illustrate themes/constructs, and identify whether the quotations were participant quotations or the author's interpretation.	p. 19-20, results
Synthesis output	Present rich, compelling and useful results that go beyond a summary of the primary studies (e.g. new interpretation, models of evidence, conceptual models, analytical framework, development of a new theory or construct).	p. 19-20, results; p.21, table 5: reset phase coding framework inductively developed through the rapid review), and p.22-25 discussion

For peer review only
